# Open lung ventilation with low tidal volumes, staircase recruitment maneuvers, high PEEP and decremental PEEP titration vs ARDSNet in ARDS: A systematic review and meta-analysis

**DOI:** 10.1016/j.jatmed.2025.08.001

**Published:** 2025-09-17

**Authors:** Siyao Zeng, Zhipeng Yao, Chunming Guan, Shanpeng Cui, Zhen Quan, Yue Li, Junbo Zheng, Hongliang Wang

**Affiliations:** a Harbin Medical University Graduate School, Harbin Medical University, Harbin 150000, China; b Department of Critical Care Medicine, The Second Affiliated Hospital of Harbin Medical University, Harbin 150000, China

**Keywords:** Acute respiratory distress syndrome, ARDS Network, Open lung ventilation, Recruitment maneuvers, Positive end-expiratory pressure

## Abstract

The impact of open lung ventilation (OLV) with low tidal volumes (TV), staircase recruitment maneuvers (SRMs), high positive end-expiratory pressure (PEEP), and decremental PEEP titration on acute respiratory distress syndrome (ARDS) patients is still uncertain. We aim to assess the impact of this OLV versus ARDS Network (ARDSNet) on patients with ARDS. We conducted a systematic review and meta-analysis of randomized controlled trials (RCTs). PubMed, Scopus, Web of Science, Embase, and Cochrane were searched from inception through August 11, 2025. Statistical analysis used risk ratios (RR), weighted mean differences (WMD), and 95 % confidence intervals (95 % CI). Based on seven RCTs involving 1545 participants, it was found that for ARDS patients whose lung recruitability has not been assessed, compared to ARDSNet, OLV does not reduce hospital mortality (RR: 1.03; 95 % CI: 0.94 to 1.13; *P* = 0.51), ICU mortality (RR: 1.04; 95 % CI: 0.94 to 1.15; *P* = 0.41), mortality at day 28 (RR: 1.08; 95 % CI: 0.97 to 1.21; *P* = 0.18), mortality at day 60 (RR: 0.86; 95 % CI: 0.62 to 1.19; *P* = 0.36), or mortality at 6 months (RR: 1.07; 95 % CI: 0.98 to 1.18; *P* = 0.14). No significant differences were found in hospital length of stay, length of ventilation, ventilator-free days to day 28, incidence of barotrauma or rates requiring prone position. However, compared to ARDSNet, OLV improves the oxygenation index (OI) at days 1, 3, and 7, while also increasing the incidence of some adverse events such as pneumothorax requiring drainage within 7 days, hypotension within 1 h, arrhythmia, and desaturation. In ARDS patients whose lung recruitability has not been assessed, the routine application of OLV with low TV, SRMs, high PEEP, and decremental PEEP titration does not improve clinical outcomes and may result in certain adverse events.

## Introduction

Acute Respiratory Distress Syndrome (ARDS) is a clinical condition defined by notable characteristics of non-cardiogenic pulmonary edema, diminished lung compliance, and hypoxemia.^1^ Each year, three million people worldwide are affected by ARDS. ARDS represents 10.4 % of all ICU admissions, with an in-hospital mortality rate of 40 %.^2^ Mechanical ventilation (MV) remains a key treatment, though it poses risks of ventilator-induced lung injury (VILI) due to improper ventilatory settings.^3^, ^4^, ^5^

The lung-protective ventilation strategy with low tidal volumes (TV) used by the ARDS Network (ARDSNet) has been adopted by physicians as the standard MV method for ARDS and applied worldwide for over 20 years. However, the mortality rate of ARDS remains very high.^6^, ^7^ In 1992, Lachmann first introduced the concept of open lung ventilation (OLV) for ARDS, including recruitment maneuvers (RMs) and high positive end-expiratory pressure (PEEP).^8^ OLV employs RMs to open collapsed alveoli and then applies high PEEP to maintain their openness. This can minimize atelectrauma and shear stress, which is considered beneficial for ARDS patients.^4^, ^9^

The most commonly used RMs involve applying a continuous positive airway pressure (CPAP) of 35–40 cmH_2_O for 40 s. However, this approach may lead to patient discomfort and even hemodynamic instability, and has not been shown to improve the prognosis of ARDS patients.^10^ Some researchers believe that using stepwise recruitment maneuvers (SRMs) approach can recruit most of the collapsed lung while minimizing hemodynamic damage and inflammation.^9^, ^11^ Studies have shown that SRMs can safely and effectively improve oxygenation and lung compliance in ARDS patients.^10^ If RMs are successful, adequate PEEP is required to keep the lungs open and prevent overdistension caused by excessive PEEP. Nevertheless, determining the optimal PEEP level after RMs remains challenging and controversial. Combining RMs with PEEP levels higher than traditional levels seems to be one approach to identify the appropriate PEEP level.^9^, ^12^ The optimal strategy for setting ARDS PEEP is generally considered to open the lungs with RMs and then perform decremental PEEP titration to determine the minimum PEEP needed to keep the lungs open.^13^ When RMs are combined with decremental PEEP titration, positive effects on the recruitment of non-ventilated lung regions can be seen within days after the maneuvers in early ARDS patients.^9^

Although the combination of SRMs and high PEEP is physiologically appealing, its effectiveness and safety remain controversial.^7^, ^9^, ^14^, ^15^ Recent guidelines from the European Society of Intensive Care Medicine (ESICM) and other bodies provide cautious recommendations regarding OLV, highlighting the need for more definitive evidence.^1^, ^16^, ^17^

This study hypothesizes that a systematic approach to OLV—incorporating low TV, SRMs, high PEEP, and decremental PEEP titration—improves clinical outcomes in ARDS patients compared to the standard ARDSNet approach, specifically regarding mortality. The objective of this systematic review and meta-analysis is to assess the impact of this specific OLV strategy on ARDS outcomes.

## Materials and methods

We registered this study with the PROSPERO (ID: CRD42024565422) and performed it following the Preferred Reporting Items for Systematic Reviews and Meta-Analyses (PRISMA) guidelines.

### Data sources and search strategy

The three team members jointly developed a detailed advanced database search strategy. Searches were conducted across five major English-language databases—PubMed, Scopus, Web of Science, Embase, and Cochrane—without language restrictions. The initial search covered the period from each database’s inception to January 3, 2025. During the revision process, the search was updated to include studies published up to August 11, 2025, and all relevant sections of the manuscript were revised accordingly. The search used Medical Subject Headings (MeSH) terms related to acute respiratory distress syndrome (e.g., “respiratory distress syndrome”, “acute lung injury”, “ARDS”), the intervention (e.g., “staircase recruitment manoeuvres”, “high positive end-expiratory pressure”, “decremental positive end-expiratory pressure titration”), the comparator (e.g., “ARDSNet”, “lung protective ventilation”, “low tidal volume”), and study type (randomized controlled trial).

#### Eligibility criteria

Randomized controlled trials (RCTs) were included if they met all of the following criteria:

1) Adult patients diagnosed with ARDS according to the Berlin definition;^18^
2) Intervention: OLV strategy combining low TV, SRMs, high PEEP, and decremental PEEP titration;
3) Comparator: ARDSNet protocol;
4) Reported at least one predefined primary or secondary outcome as described below.

Studies were excluded if they met any of the following criteria:

1) Non-randomized or observational studies, conference abstracts without full data, reviews, meta-analyses or secondary analysis;
2) Studies involving pediatric patients;
3) Interventions consisting solely of RMs, solely high PEEP, or other ventilation strategies not meeting the predefined OLV criteria;
4) Control groups that did not follow the ARDSNet protocol;
5) Incomplete or unpublished outcome data that could not be obtained from authors;
6) Animal experiments, protocols or guidelines.

### Definition of outcomes

The primary outcomes include hospital mortality, ICU mortality, mortality at day 28, mortality at day 60 and mortality at 6 months. Secondary outcomes include hospital length of stay, ICU length of stay, length of ventilation, and ventilator-free days to day 28. They also include the incidence of adverse events, such as the incidence of barotrauma, pneumothorax requiring drainage ≤ 7 days, hypotension within 1 h, arrhythmia, and desaturation. Additionally, the need for rescue therapies was measured, such as the rates requiring prone position and those requiring inhalation of nitric oxide (NO). Finally, the oxygenation index (OI) was assessed at day 1, day 3, and day 7.

### Data collection

Three reviewers screened the articles and extracted data, while another checked them. If there are discrepancies, another reviewer will be involved in deciding collectively. We constructed a table to capture these details from each included article: first author, patient characteristics, causes of ARDS, countries and study design. Additionally, we designed a table to record the initial OI, initial compliance, ventilation mode, and detailed interventions for both groups. For the OLV group, the interventions included TV, high PEEP for SRMs, decremental PEEP titration and optimal PEEP, number of RMs, and additional RMs after PEEP titration. For the ARDSNet group, this mainly included TV.

### Quality assessment

Two team members independently evaluated the quality of the included studies using the Cochrane Collaboration tool. These criteria included seven aspects: random sequence generation, allocation concealment, and blinding. If discrepancies occurred, a third reviewer was brought in to help reach a consensus.

### Statistical analysis

The analysis utilized RevMan software (version 5.3) and Stata software (version 14). We calculated risk ratios (RR) with corresponding 95 % confidence intervals (95 % CI) for dichotomous variables. Continuous variables were analyzed using the weighted mean difference (WMD) with a 95 % CI. If the original data (continuous variables) were reported as median (interquartile range), they were transformed into mean ± standard deviation (SD) using the formulas from the studies by Luo et al. and Wan et al. for the subsequent meta-analysis.^19^, ^20^ We performed the statistical analysis for values with different units after converting the units (e.g., converting hours to days). A fixed-effects model was employed when minimal or no heterogeneity was observed in the pooled outcomes. Egger’s test was conducted on outcomes to check for publication bias. If publication bias was detected (Egger’s test *P*-value < 0.05), the trim-and-fill method was used to correct or mitigate its effects. Sensitivity analysis was initially conducted using Stata software. If a specific study was found to influence the results significantly, it was excluded to assess the overall outcome using RevMan software. If the results changed substantially after the sensitivity analysis, the differences between this study and the others and its impact on OLV, were thoroughly investigated.

## Results

### Literature retrieval

**Fig. 1:**
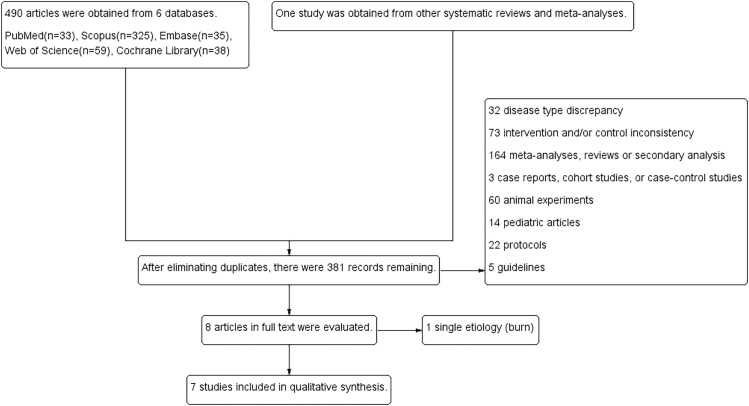
Literature screening flow chart.

A total of 490 studies were retrieved from five databases, with an additional study identified from other systematic reviews and meta-analyses. After excluding duplicates and studies not meeting the inclusion criteria, we identified seven studies for the meta-analysis (Fig. 1).^10^, ^11^, ^12^, ^21^, ^22^, ^23^, ^24^

### Characteristics of included studies

We analyzed data from 7 studies conducted between 2009 and 2019, comprising a total of 1545 subjects. These studies were conducted across 182 clinical trial centers in 20 countries. Table 1 and Table 2 provide detailed information for each study.

**Table 1:** Basic information of each study.

Study	Subjects	Gender distribution	Mean age (x ± s)	Causes of ARDS							Countries	Study design
				Pulmonary	Non-pulmonary sepsis	Acute pancreatitis	Trauma	Burn	Multiple blood transfusions	Others		
Hodgson 2019^22^	N = 114 (E: 58; C: 56)	E: M/F: 36/22 C: M/F: 30/26	E: (54.2 ± 14) C: (47.41 ± 3.78)	E: 46 C: 44	E: 8 C: 8	E: 1 C:2	E: 0 C: 1	E: 1 C: 0	E: 2 C: 0	E: 0 C: 1	Australia, Ireland, Saudi Arabia, New Zealand and the United Kingdom	Multiple center (N = 35)
Kung 2019^11^	N = 120 (E: 60; C: 60)	E: M/F: 45/15 C:M/F: 44/16	E: (66.8 ± 16.1) C: (63.7 ± 20.8)	E: 52 C: 46	E: 4 C: 8	E: 2 C: 2	NA	NA	E: 0 C: 2	E: 2 C: 2	Taiwan, Province of China	Multiple center (N = 4)
Cavalcanti 2017^24^	N = 1010 (E: 501; C: 509)	E: M/F: 313/188 C: M/F: 318/191	E: (51.3 ± 17.4) C: (50.6 ± 17.4)	E: 313 C: 313	E: 99 C: 97	NA	E: 9 C: 11	NA	E: 8 C: 3	E: 72 C: 85	Brazil, Argentina, Colombia, Italy, Poland, Portugal, Malaysia, Spain and Uruguay	Multiple center (N = 120)
Chung 2017^21^	N = 24 (E: 12; C: 12)	E: M/F: 8/4 C: M/F: 8/4	E: (67.0 ± 8.5) C: (66.7 ± 8.7)	NA	NA	NA	NA	NA	NA	NA	Taiwan, Province of China	Single center
Kacmarek 2016^23^	N = 200 (E: 99; C: 101)	E: M/F: 57/42 C: M/F: 67/34	E: (52.2 ± 15.1) C: (53.4 ± 14.5)	NA	NA	NA	NA	NA	NA	NA	USA, Spain, South Korea, Peru, Chile and Brazil	Multiple center (N = 20)
Hodgson 2011^10^	N = 20 (E: 10; C: 10)	E: M/F: 7/3 C: M/F: 6/4	E: (60 ± 5) C: (58 ± 4)	E: 5 C: 6	E: 0 C: 1	NA	E: 2 C: 0	E: 0 C: 1	NA	E: 3 C: 2	Australia	Single center
Huh 2009^12^	N = 57 (E: 30; C: 27)	E: M/F: 18/12 C: M/F: 17/10	E: (55.0 ± 3.7) C: (62.0 ± 2.2)	E: 18 C: 16	E: 7 C: 7	NA	NA	NA	E: 1 C: 2	E: 4 C: 2	South Korea	Single center

E, experimental group; C, control group; M, male; F, female; NA, not applicable.

**Table 2:** Intervention and control of each study.

Study	Initial OI	Initial compliance (mL/cmH_2_O)	Ventilation mode	OLV					ARDSNet
				TV (mL/kg)	High PEEP for SRMs	Decremental PEEP titration and optimal PEEP	Number of RMs	Additional RMs after PEEP titration	TV (mL/kg)
Hodgson 2019^22^	E: (127.7 ± 34.3) C: (129.6 ± 38.0)	E: (32.8 ± 12.7) C: (28.4 ± 11.1)	PCV (DP 15 ± 3 cmH_2_O + PEEP)	6.9 ± 1.7	PEEP 20 cmH_2_O for 2 min PEEP 30 cmH_2_O for 2 min PEEP 40 cmH_2_O for 2 min	Starting at 25 cmH_2_O, decrease by 2.5 cmH_2_O every 3 min until SpO_2_ drops by 2 % or more, or to a minimum of 15 cmH_2_O if no desaturation occurs. Perform a brief RM to raise PEEP to 2.5 cmH_2_O above the desaturation level.	Daily, for up to the first 5 days.	In case of hypoxemia. Brief RMs could be performed throughout the day in case of hypoxemia.	7.2 ± 1.8
Kung 2019^11^	E: (133.4 ± 47.0) C: (129.7 ± 42.0)	NA	PCV (DP 15 cmH_2_O + PEEP)	8.7 ± 2.1	PEEP 20 cmH_2_O for 3 breaths PEEP 23 cmH_2_O for 3 breaths PEEP 26 cmH_2_O for 3 breaths PEEP 29 cmH_2_O for 3 breaths PEEP 32 cmH_2_O for 3 breaths PEEP 35 cmH_2_O for 2 min	Lowered PEEP to 25 cmH_2_O, then decreased by 1 cmH_2_O every 3 breaths until maximum Cdyn (derecruitment point) was found. Once identified, PEEP was increased to 35 cmH_2_O for 2 min to reopen the lung, then set to 2 cmH_2_O above the derecruitment point. If not found, PEEP followed the original ARDSNet protocol.	Every 8 h until FiO_2_ is ≤ 0.4 and PEEP is ≤ 10 for at least 8 h.	After every ventilator disconnection.	8.4 ± 1.8
Cavalcanti 2017^24^	E: (119.5 ± 43.5) C: (117.2 ± 41.9)	E: (29.2 ± 12.4) C: (30.3 ± 14.4)	PCV (DP 15 cmH_2_O + PEEP)	5.8 ± 1.1	Protocol 1: PEEP 25 cmH_2_O for 1 min PEEP 35 cmH_2_O for 1 min PEEP 45 cmH_2_O for 2 min Protocol 2: PEEP 25 cmH_2_O for 1 min PEEP 30 cmH_2_O for 1 min PEEP 35 cmH_2_O for 1 min	Starting at 23 cmH_2_O, decrease by 3 cmH_2_O every 4 min (protocol 1) or 3 min (protocol 2) until reaching a minimum of 11 cmH_2_O. Measure respiratory-system static compliance after each step. The optimal PEEP is the PEEP with the best compliance plus 2 cmH_2_O.	NA	Protocol 1: Additional 2 min RMs at PEEP 45 cmH_2_O. Protocol 2: Additional RM at PEEP 35 cmH_2_O.	5.8 ± 1.0
Chung 2017^21^	E: (136 ± 43) C: (131 ± 47)	E: (31.7 ± 7.0) C: (41.5 ± 7.8)	PCV (DP 15 cmH_2_O + PEEP)	5.7 ± 0.5	PEEP 10 cmH_2_O for 40 s PEEP 15 cmH_2_O for 40 s PEEP 20 cmH_2_O for 40 s PEEP 25 cmH_2_O for 40 s PEEP 30 cmH_2_O for 40 s PEEP 35 cmH_2_O for 40 s PEEP 40 cmH_2_O for 40 s	PEEP was set to 25 cmH_2_O and reduced by 5 cmH_2_O increments, with each level held for 5 min until the final PEEP. The adjustment stopped when lung recruitment dropped by more than 2 % from the maximum recruitment level.	Only once on day 1.	NA	5.8 ± 0.4
Kacmarek 2016^23^	E: (121 ± 37) C: (114 ± 33)	E: 28.0 (22.5–36.7) C: 29.0 (21.8–38.9)	PCV (max Ppeak 50–60 cmH_2_O)	7.4 ± 1.8	PEEP 25 cmH_2_O for 1 min PEEP 30 cmH_2_O for 2 min	After 3 min of stabilization on VC and when compliance is stable, measure dynamic compliance. Then, decrease PEEP by 2 cmH_2_O steps, recording compliance after each stabilization. Continue until identifying the PEEP level with maximum compliance. Once found, recruit the lung again and set PEEP to the maximum compliance level plus 3 cmH_2_O.	After 5 days, RMs should be discontinued if patients do not show improvement (>10 % increase in compliance or >20 % increase in PaO_2_).	Desaturation or decreased compliance lasting over 10 min due to disconnection, suctioning, or turning.	7.1 ± 1.5
Hodgson 2011^10^	E: (155 ± 8) C: (149 ± 12)	E: (45.8 ± 5.4) C: (37.3 ± 5.4)	PCV (DP 15 cmH_2_O + PEEP)	NA	PEEP 20 cmH_2_O for 2 min PEEP 30 cmH_2_O for 2 min PEEP 40 cmH_2_O for 2 min	Reduce PEEP to 25, then 22.5, then 20, then 17.5, or to a minimum of 15 cmH_2_O every 3 min until SaO_2_ decreases by ≥ 1 % from the maximum. Increase PEEP to 40 cmH_2_O for 1 min, then set it to 2.5 cmH_2_O above the derecruitment point.	Once a day, until preparation for weaning.	PEEP was briefly increased to 40 cmH_2_O for 1 min if oxygen desaturation ≤ 90 % occurred or after ventilator disconnection.	NA
Huh 2009^12^	E: (115.0 ± 8.5) C: (110.8 ± 6.3)	E: (24.3 ± 7.6) C: (25.7 ± 8.1)	VCV (max Ppeak 55 cmH_2_O)	7.9 ± 1.9	2 cycles of: PEEP 15 cmH_2_O for 30 s PEEP 20 cmH_2_O for 30 s PEEP 25 cmH_2_O for 30 s	Increase PEEP by 1 cmH_2_O every 30 s from 20 cmH_2_O until a > 2 % drop in SaO_2_ and decreased static compliance are observed. This PEEP level is the alveolar collapsing pressure. The optimal PEEP after RMs is set 2 cmH_2_O above this pressure.	Once a day for 7 days.	If ventilator disconnection or if FiO_2_ requirement was increasing again.	8.0 ± 1.4

ARDSNet: acute respiratory distress syndrome Network. C, control group; Cdyn, dynamic compliance; DP, delta pressure; E, experimental group; NA, not applicable; OI, oxygenation index; PCV, pressure control ventilation; PEEP, positive end-expiratory pressure; RMs, recruitment maneuvers; SaO2, oxygen saturation; SRMs, stepwise recruitment maneuvers; TV, tidal volumes; VC, volume contro; VCV, volume control ventilation. Initial compliance values are presented as mean ± SD, except for Kacmarek 2016, where data are presented as median (IQR).

### Quality assessment

**Fig. 2:**
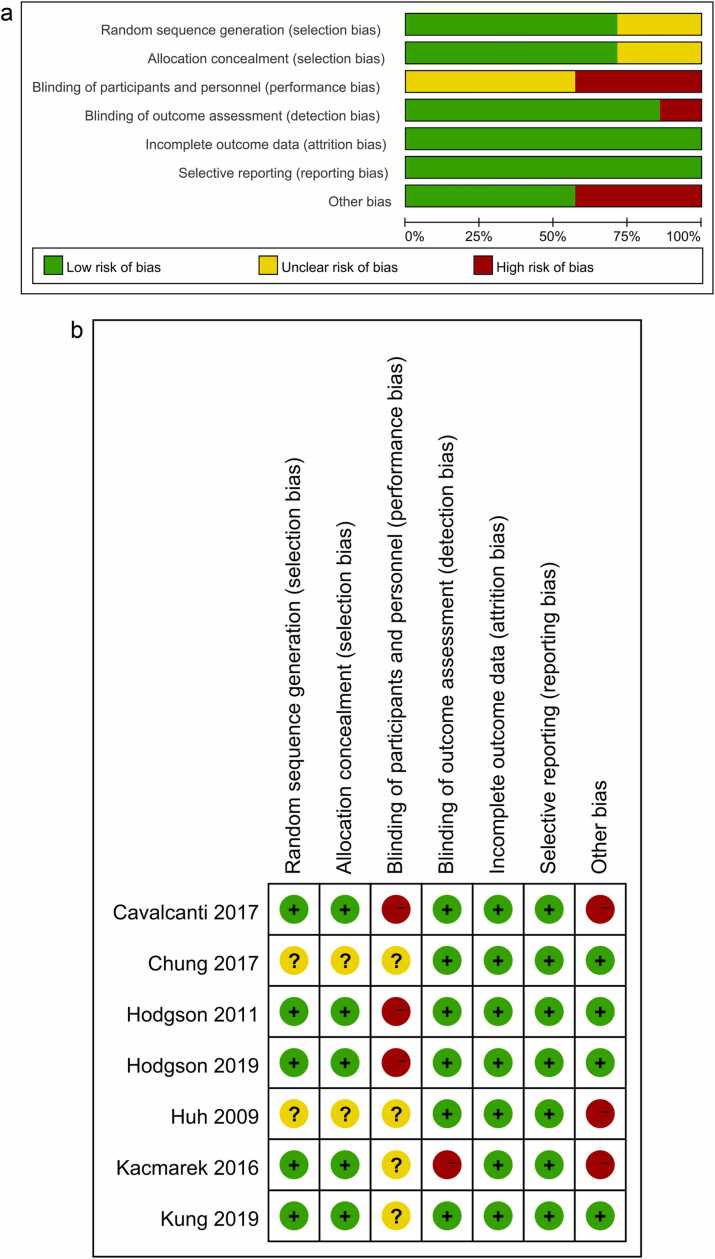
(a) Risk of bias graph. (b) Risk of bias summary.

Five studies reported random sequence generation and allocation concealment. Three studies were rated as high risk for blinding participants and personnel, and one was rated as high risk for blinding outcome assessment. All studies were rated low risk for incomplete outcome data and selective reporting. Regarding other biases, three studies were rated as high risk because they were terminated early for various reasons (Fig. 2a,b).

### Primary outcomes

#### Hospital mortality

**Fig. 3:**
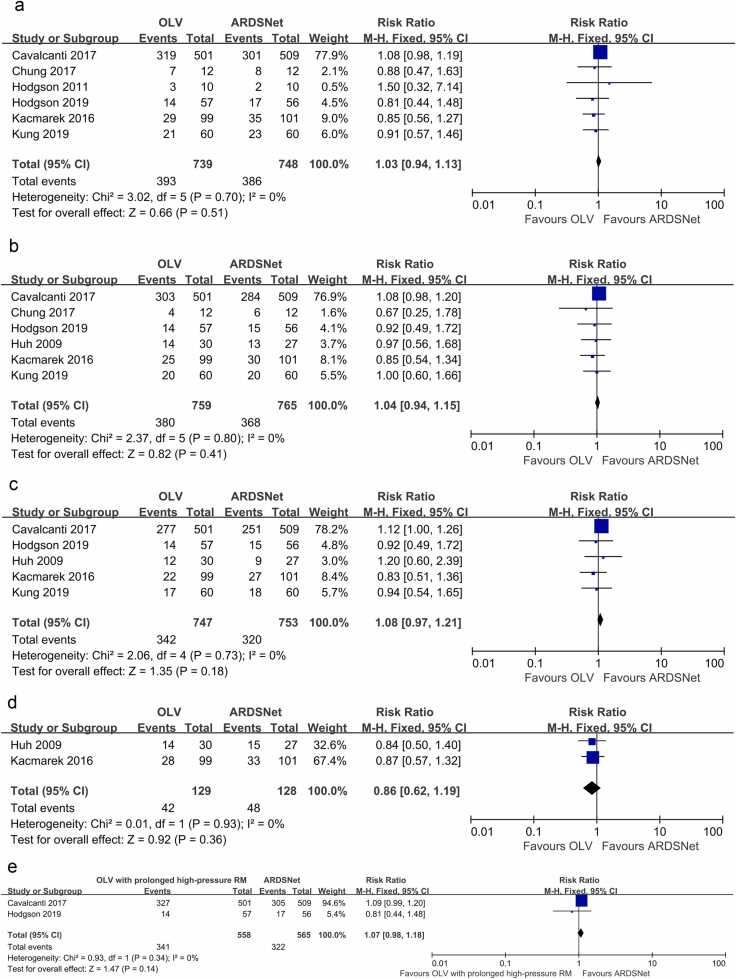
(a) Hospital mortality (forest-plot). (b) ICU mortality (forest-plot). (c) Mortality at day 28 (forest-plot). (d) Mortality at day 60 (forest-plot). (e) Mortality at 6 months (forest-plot).

The analysis included six studies (N = 1487): *I*^2^ = 0 %; RR: 1.03; 95 % CI: 0.94 to 1.13; *P* = 0.51, indicating that there was no difference in hospital mortality between the OLV group and the ARDSNet group (Fig. 3a). Sensitivity analysis indicated that the study by Cavalcanti 2017 had a significant impact on the results (Fig. S3a). After excluding Cavalcanti 2017, the results were *I*² = 0 %; RR: 0.87; 95 % CI: 0.68 to 1.12; *P* = 0.30, which remained statistically nonsignificant, indicating that the findings are relatively robust (Fig. S3b).

#### ICU mortality

The analysis included six studies (N = 1524): *I*^2^ = 0 %; RR: 1.04; 95 % CI: 0.94 to 1.15; *P* = 0.41, showing that there was no difference in ICU mortality between the OLV group and the ARDSNet group (Fig. 3b). Sensitivity analysis showed that the study by Cavalcanti 2017 had a significant impact on the results (Fig. S4a). After removing Cavalcanti 2017, the results adjusted to *I*² = 0 %; RR: 0.90; 95 % CI: 0.70 to 1.17; *P* = 0.44, which also had no statistical significance, indicating that the results are fairly stable (Fig. S4b).

#### Mortality at day 28

The analysis included five studies (N = 1500): *I*^2^ = 0 %; RR: 1.08; 95 % CI: 0.97 to 1.21; *P* = 0.18, suggesting that there was no difference in mortality at day 28 between the OLV group and the ARDSNet group (Fig. 3c). Sensitivity analysis suggested that the study by Cavalcanti 2017 had a significant impact on the results (Fig. S5a). Once Cavalcanti 2017 was excluded, the results were adjusted to *I*² = 0 %; RR: 0.93; 95 % CI: 0.70 to 1.24; *P* = 0.62, which continued to show no statistical significance, suggesting that the results are relatively stable (Fig. S5b).

#### Mortality at day 60

The analysis included two studies (N = 257): *I*^2^ = 0 %; RR: 0.86; 95 % CI: 0.62 to 1.19; *P* = 0.36, demonstrating that there was no difference in mortality at day 60 between the OLV group and the ARDSNet group (Fig. 3d).

#### Mortality at 6 months

The analysis included two studies (N = 1123): *I*^2^ = 0 %; RR: 1.07; 95 % CI: 0.98 to 1.18; *P* = 0.14, indicating that there was no difference in mortality at day 6 months between the OLV group and the ARDSNet group (Fig. 3e).

### Secondary outcomes

#### Hospital length of stay

The analysis included five studies (N = 1463): *I*^2^ = 0 %; WMD: 0.09; 95 % CI: −2.65 to 2.83; *P* = 0.95. These results indicate no difference in hospital length of stay between the OLV group and the ARDSNet group (Fig. S6a). Sensitivity analysis indicated that the study by Cavalcanti 2017 and Kacmarek 2016 significantly impacted the results (Fig. S6b). After excluding Cavalcanti 2017 and Kacmarek 2016, the results changed to *I*² = 0 %; WMD: 0.82; 95 % CI: −2.98 to 4.62; *P* = 0.67 and *I*² = 0 %; WMD: −0.85; 95 % CI: −3.94 to 2.23; *P* = 0.59 (Fig. S6c,d). These results are also not statistically significant, indicating that the results are relatively robust.

#### ICU length of stay

The analysis included seven studies (N = 1544): *I*^2^ = 72 %; WMD: 0.28; 95 % CI: −1.25 to 1.82; *P* = 0.72. These results show no difference in ICU length of stay between the OLV group and the ARDSNet group (Fig. S7a). Sensitivity analysis indicated that the study by Chung 2017 and Huh 2009 significantly impacted the results (Fig. S7b). After excluding Chung 2017 and Huh 2009, the results changed to *I*² = 46 %; WMD: 0.28; 95 % CI: −1.25 to 1.82; *P* = 0.72 and *I*² = 44 %; WMD: −2.20; 95 % CI: −3.86 to −0.55; *P* = 0.009 (Fig. S7c,d). After two sensitivity analyses, the significance of the results was inconsistent, showing that the results are not robust.

#### Length of ventilation

The analysis included five studies (N = 334): *I*^2^ = 92 %; WMD: −1.31; 95 % CI: −6.33 to 3.71; *P* = 0.61. These results suggest no difference in length of ventilation between the OLV group and the ARDSNet group (Fig. S8a). Sensitivity analysis indicated that the study by Chung 2017 and Huh 2009 significantly impacted the results (Fig. S8b). After excluding Chung 2017 and Huh 2009, the results changed to *I*² = 85 %; WMD: 0.46; 95 % CI: −3.71 to 4.63; *P* = 0.83 and *I*² = 63 %; WMD: −3.08; 95 % CI: −6.53 to 0.37; *P* = 0.08 (Fig. S8c,d), which did not reach statistical significance, indicating that the results are relatively stable.

#### Ventilator-free days to day 28

The analysis included three studies (N = 1243): *I*^2^ = 0 %; WMD: −0.94; 95 % CI: −1.93 to 0.05; *P* = 0.06. These findings demonstrate no difference in ventilator-free days by day 28 between the OLV group and the ARDSNet group (Fig. S9a). Sensitivity analysis indicated that the study by Cavalcanti 2017 had a significant impact on the results (Fig. S9b). After excluding Cavalcanti 2017, the results were *I*² = 0 %; WMD: 1.38; 95 % CI: −2.54 to 5.30; *P* = 0.49, which showed no significant difference, suggesting that the results remain reliable (Fig. S9c).

### Adverse events

#### Incidence of barotrauma

The analysis included five studies (N = 1500): *I*^2^ = 67 %; RR: 1.04; 95 % CI: 0.49 to 2.20; *P* = 0.91. These findings indicate no difference in incidence of barotrauma between the OLV group and the ARDSNet group (Fig. S10a). Sensitivity analysis indicated that the study by Cavalcanti 2017 had a significant influence on the results (Fig. S10b). After excluding Cavalcanti 2017, the results changed to *I*² = 0 %; RR: 0.77; 95 % CI: 0.49 to 1.21; *P* = 0.25, which also had no statistical significance, indicating that the findings demonstrate robustness (Fig. S10c).

#### Incidence of pneumothorax requiring drainage ≤ 7 days

The analysis included two studies (N = 1123): *I*² = 0 %; RR: 2.40; 95 % CI: 1.06 to 5.42; *P* = 0.04, showing that the OLV group significantly increased the incidence of pneumothorax requiring drainage ≤ 7 days compared to the ARDSNet group (Fig. S11).

#### Incidence of hypotension within 1 h

The analysis included two studies (N = 1123): *I*² = 0 %; RR: 1.24; 95 % CI: 1.04 to 1.49; *P* = 0.02, suggesting that the OLV group significantly increased the incidence of hypotension within 1 h compared to the ARDSNet group (Fig. S12).

#### Incidence of arrhythmia

The analysis included three studies (N = 434): *I*^2^ = 17 %; RR: 2.19; 95 % CI: 1.29 to 3.71; *P* = 0.004, demonstrating that the OLV group significantly increased the incidence of arrhythmia compared to the ARDSNet group (Fig. S13a). Sensitivity analysis indicated that the study by Kacmarek 2016 significantly influenced the results (Fig. S13b). After excluding Kacmarek 2016, the results changed to *I*² = 29 %; RR: 3.04; 95 % CI: 1.42 to 6.52; *P* = 0.004, which is not markedly different from the previous results, showing that the results remain reliable (Fig. S13c).

#### Incidence of desaturation

The analysis included three studies (N = 340): *I*^2^ = 31 %; RR: 1.90; 95 % CI: 1.22 to 2.97; *P* = 0.005, indicating that the OLV group significantly increased the incidence of desaturation compared to the ARDSNet group (Fig. S14a). Sensitivity analysis indicated that the study by Kacmarek 2016 significantly impacted the results (Fig. S14b). After excluding Kacmarek 2016, the results changed to *I*² = 0 %; RR: 9.00; 95 % CI: 1.19 to 68.07; *P* = 0.03. The incidence of desaturation significantly increased, indicating the results are not very stable (Fig. S14c). Although the results are not robust, the trend of incidence of desaturation is rising.

#### Rates requiring prone position

The analysis included three studies (N = 371): *I*^2^ = 42 %; RR: 0.75; 95 % CI: 0.47 to 1.19; *P* = 0.22. These findings show no difference in rates requiring prone position between the OLV group and the ARDSNet group (Fig. S15a). Sensitivity analysis indicated that the study by Huh 2009 markedly affected the findings (Fig. S15b). After excluding Huh 2009, the results changed to *I*² = 0 %; RR: 0.47; 95 % CI: 0.21 to 1.05; *P* = 0.07, which also had no statistical significance, indicating that the results are relatively robust (Fig. S15c).

#### Rates requiring inhalation of NO

The analysis included three studies (N = 191): *I*^2^ = 65 %; RR: 0.63; 95 % CI: 0.23 to 1.71; *P* = 0.37, suggesting that there was no difference in rates requiring inhalation of NO between the OLV group and the ARDSNet group (Fig. S16a). Sensitivity analysis using Stata indicated that the study by Hodgson 2019 and Huh 2009 exerted a significant effect on the results (Fig. S16b). After excluding Hodgson 2019 and Huh 2009, the results changed to *I*^2^ = 0 %; RR: 1.03; 95 % CI: 0.62 to 1.72; *P* = 0.91 and *I*^2^ = 0 %; RR: 0.36; 95 % CI: 0.16 to 0.83; *P* = 0.02 (Fig. S16c,d). After two sensitivity analyses, the significance of the results was inconsistent, indicating that the results are not robust.

#### OI at day 1

The analysis included three studies (N = 434): *I*^2^ = 79 %; WMD: 53.08; 95 % CI: 26.52 to 79.64; *P* < 0.01, demonstrating that the OLV group significantly increased the OI at day 1 compared to the ARDSNet group (Fig. S17a). Sensitivity analysis indicated that the study by Kung 2019 played a critical role in shaping the results (Fig. S17b). After excluding Kung 2019, the results changed to *I*² = 0 %; WMD: 65.91; 95 % CI: 51.92 to 79.90; *P* < 0.01, which is not markedly different from the previous results, indicating that the results are relatively robust (Fig. S17c).

#### OI at day 3

The analysis included four studies (N = 442): *I*^2^ = 33 %; WMD: 52.70; 95 % CI: 40.86 to 64.53; *P* < 0.01, indicating that the OLV group significantly increased the OI at day 3 compared to the ARDSNet group (Fig. S18a). Sensitivity analysis indicated that the study by Kacmarek 2016 and Kung 2019 significantly impacted the results (Fig. S18b). After excluding Kacmarek 2016 and Kung 2019, the results changed to *I*² = 19 %; WMD: 46.27; 95 % CI: 31.49 to 61.05; *P* < 0.01 and *I*² = 0 %; WMD: 58.29; 95 % CI: 44.65 to 71.93; *P* < 0.01 (Fig. S18c,d), which also had no statistical significance, showing that the results are relatively stable.

#### OI at day 7

The analysis included three studies (N = 340): *I*^2^ = 0 %; WMD: 25.28; 95 % CI: 8.42 to 42.14; *P* = 0.003, indicating that the OLV group significantly increased the OI at day 7 compared to the ARDSNet group (Fig. S19a). No study significantly impacted the results when conducting sensitivity analysis using Stata (Fig. S19b).

### Publication bias

Egger’s test was performed for all outcomes, revealing publication bias in one outcome (ICU mortality). Applying the trim-and-fill method yielded results similar to the original findings (RR: 1.06; 95 % CI: 0.96 to 1.16; *P* = 0.27), suggesting that the meta-analysis results are robust.

Detailed information for all outcomes can be found in Table 3.

**Table 3:** The summary of outcomes.

**Sequence number**	**Outcomes**	**Data analysis**								**Robust (after sensitivity analysis)**	***P*****-value of Egger's test**	**Publication bias**
		**Included studies**	**Subjects**	**RR**	**WMD**	**95 % CI**	***I***^**2**^	**Effect model**	***P*****-value**			
a1	Hospital mortality	6	1487	1.03	NA	0.94, 1.13	0 %	Fixed	0.51	NA	0.154	×
a1*	Hospital mortality (Delete Cavalcanti 2017 ^24^)	5	477	0.87	NA	0.68, 1.12	0 %	Fixed	0.30	✓	NA	NA
a2	ICU mortality	6	1524	1.04	NA	0.94, 1.15	0 %	Fixed	0.41	NA	0.015	✓
a2*	ICU mortality (Delete Cavalcanti 2017 ^24^)	5	514	0.90	NA	0.70, 1.17	0 %	Fixed	0.44	✓	NA	NA
a3	Mortality at day 28	5	1500	1.08	NA	0.97, 1.21	0 %	Fixed	0.18	NA	0.193	×
a3*	Mortality at day 28 (Delete Cavalcanti 2017 ^24^)	4	490	0.93	NA	0.70, 1.24	0 %	Fixed	0.62	✓	NA	NA
a4	Mortality at day 60	2	257	0.86	NA	0.62, 1.19	0 %	Fixed	0.36	NA	NA	NA
a5	Mortality at 6 months	2	1123	1.07	NA	0.98, 1.18	0 %	Fixed	0.14	NA	NA	NA
b1	Hospital length of stay	5	1463	NA	0.09	−2.65, 2.83	0 %	Fixed	0.95	NA	0.654	×
b1*	Hospital length of stay (Delete Cavalcanti 2017 ^24^)	4	453	NA	0.82	−2.98, 4.62	0 %	Fixed	0.67	✓	NA	NA
b1**	Hospital length of stay (Delete Kacmarek 2016 ^23^)	4	1263	NA	−0.85	−3.94, 2.23	0 %	Fixed	0.59	✓	NA	NA
b2	ICU length of stay	7	1544	NA	−1.40	−4.24, 1.43	72 %	Random	0.33	NA	0.225	×
b2*	ICU length of stay (Delete Chung 2017 ^21^)	6	1520	NA	0.28	−1.25, 1.82	46 %	Fixed	0.72	×	NA	NA
b2**	ICU length of stay (Delete Huh 2009 ^12^)	6	1487	NA	−2.20	−3.86, −0.55	44 %	Fixed	0.009		NA	NA
c1	Length of ventilation	5	334	NA	−1.31	−6.33, 3.71	92 %	Random	0.61	NA	0.090	×
c1*	Length of ventilation (Delete Chung 2017 ^21^)	4	310	NA	0.46	−3.71, 4.63	85 %	Random	0.83	✓	NA	NA
c1**	Length of ventilation (Delete Huh 2009 ^12^)	4	277	NA	−3.08	−6.53, 0.37	63 %	Random	0.08	✓	NA	NA
c2	Ventilator-free days to day 28	3	1243	NA	−0.94	−1.93, 0.05	0 %	Fixed	0.06	NA	0.294	×
c2*	Ventilator-free days to day 28 (Delete Cavalcanti 2017 ^24^)	2	233	NA	1.38	−2.54, 5.30	0 %	Fixed	0.49	✓	NA	NA
d1	Incidence of barotrauma	5	1500	1.04	NA	0.49, 2.20	67 %	Random	0.91	NA	0.567	×
d1*	Incidence of barotrauma (Delete Cavalcanti 2017 ^24^)	4	490	0.77	NA	0.49, 1.21	0 %	Fixed	0.25	✓	NA	NA
d2	Incidence of pneumothorax requiring drainage ≤ 7 days	2	1123	2.40	NA	1.06, 5.42	0 %	Fixed	0.04	NA	NA	NA
d3	Incidence of r hypotension within 1 h	2	1123	1.24	NA	1.04, 1.49	0 %	Fixed	0.02	NA	NA	NA
d4	Incidence of arrhythmia	3	434	2.19	NA	1.29, 3.71	17 %	Fixed	0.004	NA	0.296	×
d4*	Incidence of arrhythmia (Delete Kacmarek 2016 ^23^)	2	234	3.04	NA	1.42, 6.52	29 %	Fixed	0.004	✓	NA	NA
d5	Incidence of desaturation	3	340	1.90	NA	1.22, 2.97	31 %	Fixed	0.005	NA	0.081	×
d5*	Incidence of desaturation (Delete Kacmarek 2016 ^23^)	2	140	9.00	NA	1.19, 68.07	0 %	Fixed	0.03	×	NA	NA
d6	Rates requiring prone position	3	371	0.75	NA	0.47, 1.19	42 %	Fixed	0.22	NA	0.162	×
d6*	Rates requiring prone position (Delete Huh 2009 ^12^)	2	314	0.47	NA	0.21, 1.05	0 %	Fixed	0.07	✓	NA	NA
d7	Rates requiring inhalation of NO	3	191	0.63	NA	0.23, 1.71	65 %	Random	0.37	NA	0.576	×
d7*	Rates requiring inhalation of NO (Delete Hodgson 2019 ^22^)	2	77	1.03	NA	0.62, 1.72	0 %	Fixed	0.91	×	NA	NA
d7**	Rates requiring inhalation of NO (Delete Huh 2009 ^12^)	2	134	0.36	NA	0.16, 0.83	0 %	Fixed	0.02		NA	NA
e1	OI at day 1	3	434	NA	53.08	26.52, 79.64	79 %	Random	< 0.01	NA	0.703	×
e1*	OI at day 1 (Delete Kung 2019)	2	314	NA	65.91	51.92, 79.90	0 %	Fixed	< 0.01	✓	NA	NA
e2	OI at day 3	4	442	NA	52.70	40.86, 64.53	33 %	Fixed	< 0.01	NA	0.325	×
e2*	OI at day 3 (Delete Kacmarek 2016 ^23^)	3	242	NA	46.27	31.49, 61.05	19 %	Fixed	< 0.01	✓	NA	NA
e2**	OI at day 3 (Delete Kung 2019)	3	330	NA	58.29	44.65, 71.93	0 %	Fixed	< 0.01	✓	NA	NA
e3	OI at day 7	3	340	NA	25.28	8.42, 42.14	0 %	Fixed	0.003	✓	0.571	×

ICU, intensive care unit; NA, not applicable; NO, nitric oxide; OI, oxygenation index; RR, risk ratios; WMD, weighted mean difference.

## Discussion

Our systematic review and meta-analysis results indicate that compared to ARDSNet, OLV with low TV, SRMs, high PEEP, and decremental PEEP titration does not reduce various mortality rates in ARDS patients. Second, this particular OLV strategy does not appear to reduce hospital length of stay, length of ventilation, or ventilator-free days to day 28. Additionally, regarding adverse events, this OLV does not increase the incidence of barotrauma but does increase the incidence of pneumothorax requiring drainage ≤ 7 days, hypotension within 1 h, arrhythmia and desaturation. Regarding the need for rescue therapies, this OLV does not reduce the rates requiring the prone position. Finally, this OLV improves the OI at day 1, 3, and 7 in ARDS patients. However, we cannot determine the effect of OLV on ICU length of stay and rates of requiring inhalation of NO.

Sensitivity analyses were conducted by sequentially excluding studies for each outcome, revealing that three secondary outcomes were not robust, whereas the majority remained stable. The studies excluded in the sensitivity analysis were mostly from Cavalcanti 2017 (the Alveolar Recruitment for ARDS trial, ART), which has been highly controversial among experts in recent years.^24^ The ART trial was a large clinical study conducted in 120 ICUs across nine countries, including 104 in Brazil, involving 1010 participants. It is the largest RCT included in our systematic review and meta-analysis. The main conclusions were that in ARDS patients, OLV significantly increased mortality at day 28, mortality at 6 months, incidence of barotrauma, and incidence of pneumothorax requiring drainage ≤ 7 days compared to ARDSNet. In the ART trial, only the incidence of pneumothorax requiring drainage ≤ 7 days higher, in agreement with another study, whereas other outcomes reflecting the disadvantages of OLV for ARDS patients (such as increased mortality) contradicted findings from other studies.^11^, ^12^, ^22^, ^23^, ^24^ The ESICM 2023 ARDS guidelines classifies the ART trial as a high-quality study.^1^ The guidelines do not recommend prolonged high-pressure RMs, primarily based on the ART trial, as it led to many adverse outcomes.^1^, ^24^ However, since its publication in 2017, ART has faced criticism from some experts due to flaws in its study design, methods, and data analysis, as well as considerable deficiencies in the healthcare system of its main implementing country, Brazil.^25^, ^26^, ^27^ The increased mortality in the OLV group of the ART trial may be explained by the following factors: 1) The absence of exclusion criteria to determine patients' prior conditions, potentially including those with significant organ failure or comorbidities (such as chronic liver failure, metastatic disease, etc.), who often have high mortality rates; 2) Whether the medical staff at the clinical trial centers had the skills required to implement and effectively execute such a complex clinical protocol; 3) Differences in the level of nursing care across the clinical trial centers; 4) Deficiencies in the design of RMs and ventilation methods in the OLV group; 5) Approximately 63 % of the patients in the OLV group had direct lung injury (e.g., pneumonia).^25^, ^26^, ^27^ A study using machine learning for cluster analysis of the ART trial population showed that ARDS patients with pneumonia as the primary cause had higher mortality rates when using OLV. This direct lung injury is often asymmetric and heterogeneous, making RMs more challenging.^28^ Moreover, the ART trial did not consider whether patients were responsive to RMs when randomly assigning them to the OLV or ARDSNet groups. Many patients in ART likely had low recruitability and were exposed to unnecessarily high pressures.^24^, ^25^, ^26^, ^27^, ^28^ Therefore, the use of the ART trial as the primary evidence against prolonged high-pressure RMs in the ESICM 2023 ARDS guidelines may have some limitations. The sensitivity analysis revealed that excluding the ART trial, which had a substantial impact on the results, did not lead to significant changes in the outcomes, demonstrating the robustness of the findings. Consequently, although the ART trial-the largest included study-individually indicated that OLV was detrimental to ARDS patients compared with ARDSNet, the final results of our meta-analysis demonstrated that the OLV did not increase mortality or barotrauma, although it was associated with a higher incidence of other adverse events. Additionally, the sensitivity analysis showed that certain results were significantly influenced by some studies. The likely reason for this impact is clinical heterogeneity, including differences in interventions such as ventilation mode, timing of RMs, decremental PEEP titration and optimal PEEP, number of RMs, and additional RMs after PEEP titration.

Our meta-analysis includes studies that mostly align with the meta-analysis in the supplementary materials of the ESICM 2023 ARDS guidelines. The meta-analysis conducted in the guideline searched only PubMed and, according to its classification criteria (prolonged high-pressure RMs and brief high-pressure RMs),excluded certain studies, such as those by Amato et al., Constantin et al., Khan et al., and Yang et al.^29^, ^30^, ^31^, ^32^ Incorporating these studies could influence the final results of the meta-analysis. Nonetheless, these studies did not meet our predefined inclusion criteria. Additionally, the guideline’s meta-analysis did not perform statistical analyses using interquartile ranges for outcomes such as hospital length of stay, ICU length of stay, and length of ventilation. Furthermore, our meta-analysis includes results that were not analyzed in the guideline’s meta-analysis, such as the incidence of other adverse events, the need for rescue therapies, and the OI on days 1, 3, and 7. These combined results provide a more comprehensive reflection of the impact of OLV on ARDS patients. In our meta-analysis, we did not differentiate between prolonged high-pressure RMs and brief high-pressure RMs. Currently, there are no clinical trials or animal studies indicating a significant difference between prolonged high-pressure RMs and brief high-pressure RMs, especially with a 60-second threshold. A strict distinction between these categories may be of limited value.^33^ Therefore, we focused on the overall impact of OLV on ARDS.

In 2017, Lu et al. published the first meta-analysis comparing OLV with other ventilation strategies. Their paper claimed that OLV could reduce hospital mortality, 28-day mortality, and ICU mortality compared to other strategies. However, their analysis included numerous studies where the interventions were solely RMs or high PEEP, which cannot be strictly classified as OLV. Additionally, their control groups were not exclusively ARDSNet. Moreover, their study did not include the ART trial or the two studies from 2019, which may render their results inaccurate.^11^, ^22^, ^24^, ^34^ Consequently, their conclusions may be unconvincing. Another meta-analysis from 2021 showed that OLV did not improve 28–30 days mortality rates. However, this analysis included studies where the interventions were either solely RMs or solely high PEEP, and even studies that did not involve RMs or high PEEP, which cannot be regarded as true OLV.^35^ In recent years, numerous systematic reviews and meta-analyses have compared RMs and non-RMs for ARDS, some of which incorporated high TV, high PEEP or PEEP titration, which differ substantially from the OLV protocol assessed in our meta-analysis. Most of these studies concluded that RMs do not reduce mortality in ARDS patients compared to non-RMs.^36^, ^37^, ^38^, ^39^, ^40^, ^41^, ^42^, ^43^ To our knowledge, this systematic review and meta-analysis is the first to compare the effects of OLV with low TV, SRMs, high PEEP, and decremental PEEP titration—with ARDSNet on ARDS patients.

The “baby lung” model describes the pathophysiology of ARDS, where uneven lung compliance leads to overinflation of healthy regions while damaged areas remain collapsed. During mechanical ventilation, air preferentially flows to open regions, increasing the risk of overinflation. This model highlights the vulnerability and heterogeneity of ARDS lungs and the importance of assessing lung recruitability.^4^, ^44^ The core of OLV is RMs, which reopen collapsed alveoli to improve gas exchange. Before applying RMs, it is crucial to assess lung recruitability to identify patients who may benefit while minimizing the risk of overinflation in those with limited recruitability.^9^, ^45^ In ARDS, lung morphology (focal or diffuse) impacts recruitability more than etiology. Focal ARDS has poorer recruitability, making RMs risky due to overdistension and increased shunting, potentially worsening the condition. In contrast, patients with diffuse ARDS generally exhibit greater lung recruitability, rendering RMs more effective.^9^, ^46^ Patients with focal ARDS may benefit from lower PEEP to avoid hemodynamic issues, while those with diffuse ARDS may require higher PEEP to reduce atelectasis.^47^ Lung recruitability varies significantly in ARDS, with CT data showing that about 24 % of patients have nearly non-recruitable lung tissue. This highlights the need for individualized assessment before applying RMs and high PEEP. In non-recruitable patients, increasing PEEP may lead to overdistension and harmful effects. Therefore, evaluating recruitability before OLV is crucial to avoid adverse outcomes.^4^, ^48^ The OLV studies in this review did not assess lung recruitability before randomizing patients to OLV or ARDSNet groups. Although safer SRMs and decremental PEEP titration were used, the lack of recruitability assessment may have impacted the effectiveness and safety of OLV strategies.

Our systematic review has several limitations. Significant heterogeneity in patient populations, ARDS severity, and ventilation protocols complicates comparisons and influence outcomes. Sensitivity analysis reduced statistical heterogeneity but did not fully address clinical diversity. Additionally, two studies had very small sample sizes (*N* < 30), and data for some secondary outcomes were limited. Moreover, we did not differentiate between prolonged and brief high-pressure RMs, which may have introduced classification bias. Nonetheless, we acknowledge that potential differences by RM duration cannot be entirely excluded and should be explored in future research. Finally, the lack of lung recruitability assessment may have affected the effectiveness and safety of OLV.

Individual variability in lung recruitability among ARDS patients significantly impacts the effectiveness of OLV. Performing RMs and setting high PEEP without assessing recruitability can cause adverse effects. Therefore, assessing lung recruitability before OLV is crucial. Ongoing trials are exploring individualized OLV approaches guided by recruitability assessment.^49^, ^50^ Future research should focus on evaluating recruitability and tailoring ventilator settings accordingly to improve safety and effectiveness. Well-designed RCTs are needed to provide stronger evidence for optimizing OLV treatment and guiding clinical practice to improve patient outcomes.

## Conclusions

For ARDS patients whose lung recruitability has not been assessed, the systematic use of OLV—including low TV, SRMs, high PEEP, and decremental PEEP titration—fails to improve clinical outcomes and results in certain adverse events. These findings suggest that routine application of this OLV strategy in unselected ARDS populations should be approached with caution. Future research should focus on identifying patient subgroups most likely to benefit—particularly those with high lung recruitability—and on optimizing recruitment and PEEP titration protocols to balance potential benefits with the risk of harm.

## CRediT authorship contribution statement

**Chunming Guan:** Visualization, Software, Formal analysis, Data curation. **Zhipeng Yao:** Visualization, Software, Formal analysis, Data curation. **Siyao Zeng:** Writing – review & editing, Writing – original draft, Visualization, Software, Methodology, Investigation, Formal analysis, Conceptualization. **Hongliang Wang:** Writing – review & editing, Validation, Supervision, Methodology, Investigation, Funding acquisition. **Junbo Zheng:** Writing – review & editing, Validation, Supervision, Methodology, Investigation, Conceptualization. **Yue Li:** Writing – review & editing, Validation, Supervision, Funding acquisition, Data curation. **Zhen Quan:** Software. **Shanpeng Cui:** Visualization, Software, Formal analysis, Data curation. All authors have read and agreed to the published version of the manuscript.

## Consent for publication

Not applicable.

## Ethical statement

Not applicable.

## Funding

The National Key Research and Development Program of China (Nos. 2021YFC2501800), 10.13039/501100001809National Natural Science Foundation of China (No. 82472184), The Outstanding Youth Project of Heilongjiang Natural Science Foundation (Nos. JQ2021H002), Key R&D Plan Project in Heilongjiang Province (No. GY2023ZB0075), Harbin Medical University Foundation Youth Project (No. PYQN2023–9), 10.13039/100007452Wu Jieping Medical Foundation (No. 320.6750.2021–4–60), Research Project of Heilongjiang Provincial Health Commission (No. 20241717010028).

## Declaration of competing interest

The authors declare that they have no known competing financial interests or personal relationships that could have appeared to influence the work reported in this paper.

## Data Availability

The datasets generated and analyzed during the current study are available from the corresponding author upon reasonable request.
