# Supplementary material for: Open lung ventilation with low tidal volumes, staircase recruitment maneuvers, high PEEP and decremental PEEP titration vs ARDSNet in ARDS: A systematic review and meta-analysis
Source: J Anesth Transl Med. 2025 Sep 17;4(3):148–60. doi: 10.1016/j.jatmed.2025.08.001 (PMC13001771; doi:10.1016/j.jatmed.2025.08.001)
Supplement: Supplementary file 1 — Supplementary material [file mmc1.zip › Supplementary information/Supplementary information/Supplementary content.docx]

**Supporting information**

**Supplementary content**

Supplementary Material 1. Search strategy

Supplementary Material 2. PRISMA_2020_checklist

Fig. S3a. Hospital mortality (sensitivity analysis)

Fig. S3b. Hospital mortality (delete Cavalcanti 2017) (forest-plot)

Fig. S4a. ICU mortality (sensitivity analysis)

Fig. S4b. ICU mortality (delete Cavalcanti 2017) (forest-plot)

Fig. S5a. Mortality at day 28 (sensitivity analysis)

Fig. S5b. Mortality at day 28 (delete Cavalcanti 2017) (forest-plot)

Fig. S6a. Hospital length of stay (forest-plot)

Fig. S6b. Hospital length of stay (sensitivity analysis)

Fig. S6c. Hospital length of stay (delete Cavalcanti 2017) (forest-plot)

Fig. S6d. Hospital length of stay (delete Kacmarek 2016) (forest-plot)

Fig. S7a. ICU length of stay (forest-plot)

Fig. S7b. ICU length of stay (sensitivity analysis)

Fig. S7c. ICU length of stay (delete Chung 2017) (forest-plot)

Fig. S7d. ICU length of stay (delete Huh 2009) (forest-plot)

Fig. S8a. Length of ventilation (forest-plot)

Fig. S8b. Length of ventilation (sensitivity analysis)

Fig. S8c. Length of ventilation (delete Chung 2017) (forest-plot)

Fig. S8d. Length of ventilation (delete Huh 2009) (forest-plot)

Fig. S9a. Ventilator-free days to day 28 (forest-plot)

Fig. S9b. Ventilator-free days to day 28 (sensitivity analysis)

Fig. S9c. Ventilator-free days to day 28 (delete Cavalcanti 2017) (forest-plot)

Fig. S10a. Incidence of barotrauma (forest-plot)

Fig. S10b. Incidence of barotrauma (sensitivity analysis)

Fig. S10c. Incidence of barotrauma (delete Cavalcanti 2017) (forest-plot)

Fig. S11. Incidence of pneumothorax requiring drainage ≤ 7 days (forest-plot)

Fig. S12. Incidence of r hypotension within 1 hour (forest-plot)

Fig. S13a. Incidence of arrhythmia (forest-plot)

Fig. S13b. Incidence of arrhythmia (sensitivity analysis)

Fig. S13c. Incidence of arrhythmia (delete Kacmarek 2016) (forest-plot)

Fig. S14a. Incidence of desaturation (forest-plot)

Fig. S14b. Incidence of desaturation (sensitivity analysis)

Fig. S14c. Incidence of desaturation (delete Kacmarek 2016) (forest-plot)

Fig. S15a. Rates requiring prone position (forest-plot)

Fig. S15b. Rates requiring prone position (sensitivity analysis)

Fig. S15c. Rates requiring prone position (delete Huh 2009) (forest-plot)

Fig. S16a. Rates requiring inhalation of NO (forest-plot)

Fig. S16b. Rates requiring inhalation of NO (sensitivity analysis)

Fig. S16c. Rates requiring inhalation of NO (delete Hodgson 2019) (forest-plot)

Fig. S16d. Rates requiring inhalation of NO (delete Huh 2009) (forest-plot)

Fig. S17a. OI at day 1 (forest-plot)

Fig. S17b. OI at day 1 (sensitivity analysis)

Fig. S17c. OI at day 1 (delete Kung 2019) (forest-plot)

Fig. S18a. OI at day 3 (forest-plot)

Fig. S18b. OI at day 3 (sensitivity analysis)

Fig. S18c. OI at day 3 (delete Kacmarek 2016) (forest-plot)

Fig. S18d. OI at day 3 (delete Kung 2019) (forest-plot)

Fig. S19a. OI at day 7 (forest-plot)

Fig. S19b. OI at day 7 (sensitivity analysis)
