# Supplementary figures and images for: Open lung ventilation with low tidal volumes, staircase recruitment maneuvers, high PEEP and decremental PEEP titration vs ARDSNet in ARDS: A systematic review and meta-analysis
Source: J Anesth Transl Med. 2025 Sep 17;4(3):148–60. doi: 10.1016/j.jatmed.2025.08.001 (PMC13001771; doi:10.1016/j.jatmed.2025.08.001)

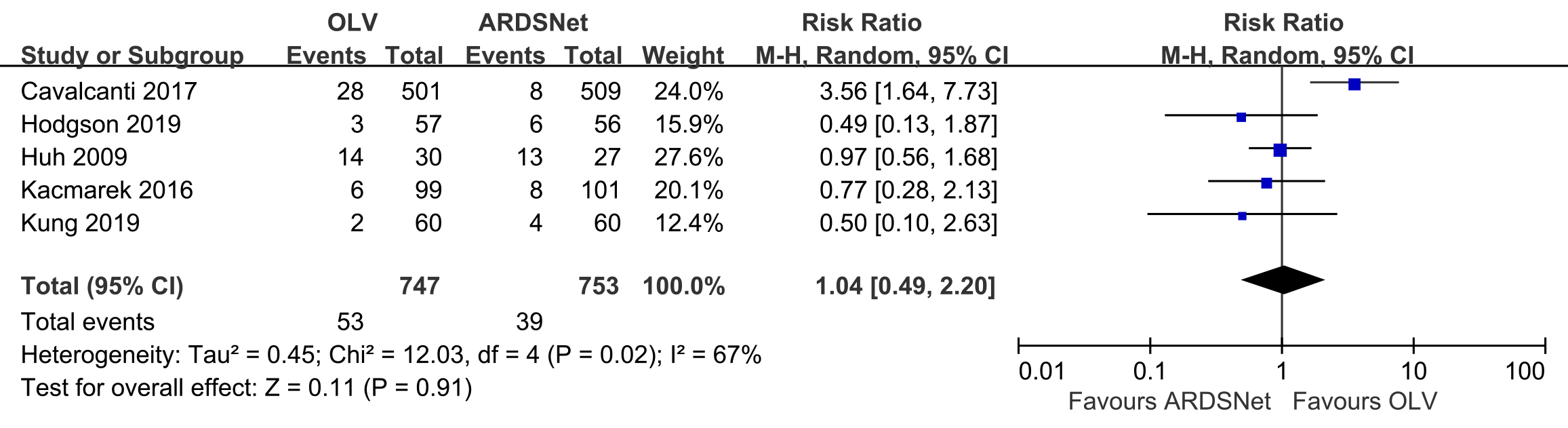

Supplement: Supplementary file 1 — Supplementary material [file mmc1.zip › Supplementary information/Supplementary information/Figure. S10a.tif]

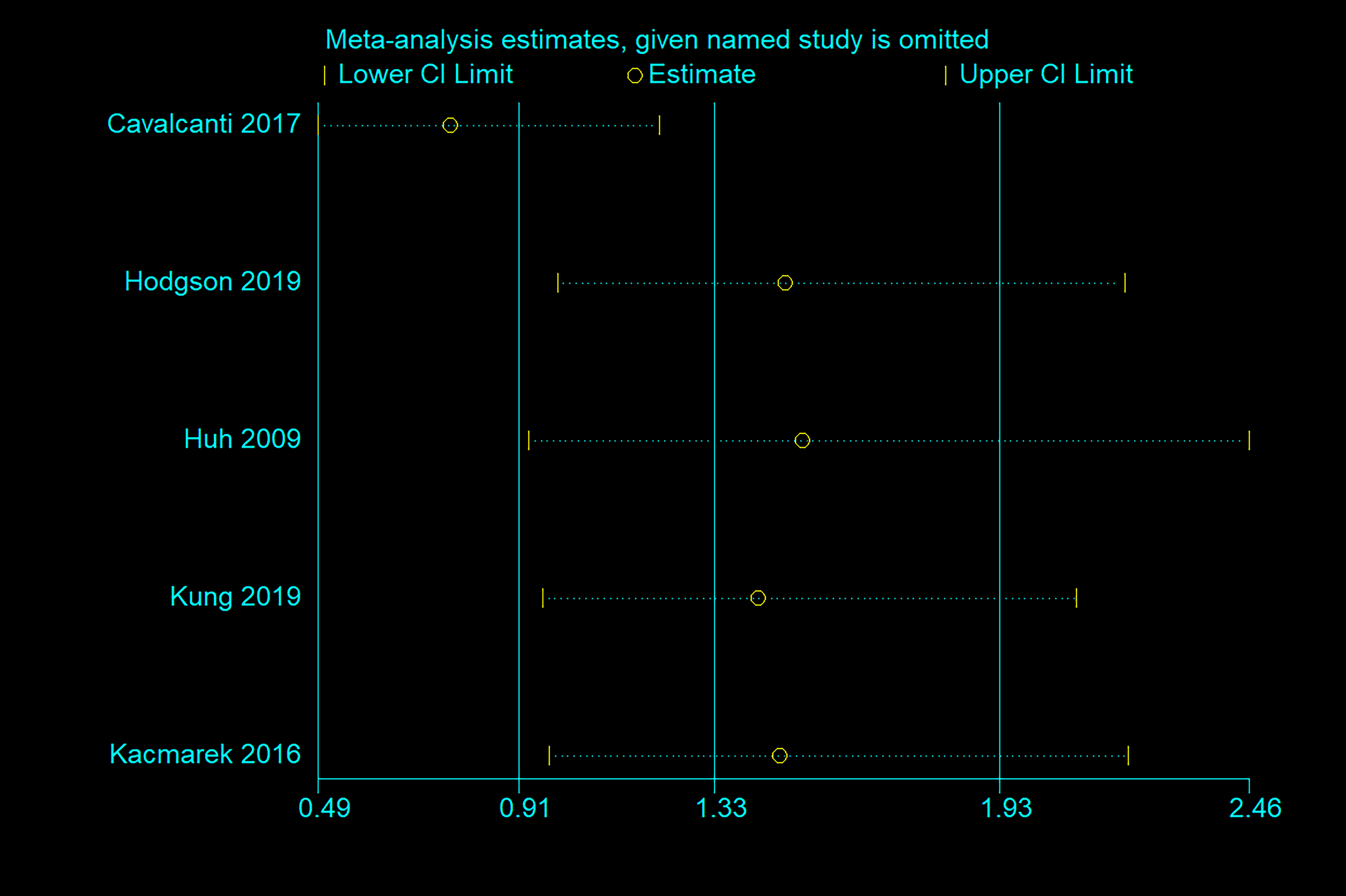

Supplement: Supplementary file 1 — Supplementary material [file mmc1.zip › Supplementary information/Supplementary information/Figure. S10b.tif]

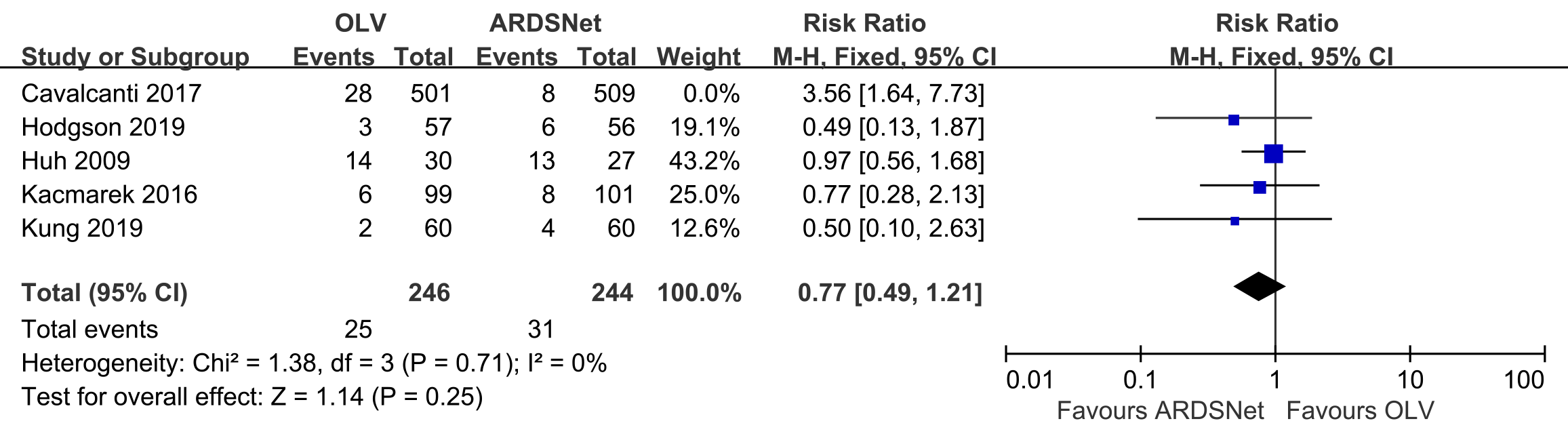

Supplement: Supplementary file 1 — Supplementary material [file mmc1.zip › Supplementary information/Supplementary information/Figure. S10c.tif]

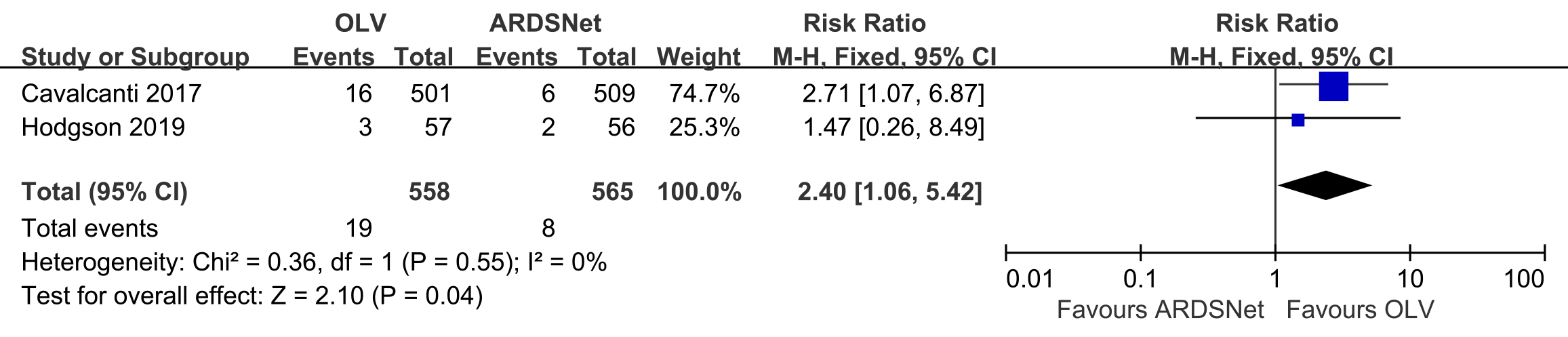

Supplement: Supplementary file 1 — Supplementary material [file mmc1.zip › Supplementary information/Supplementary information/Figure. S11.tif]

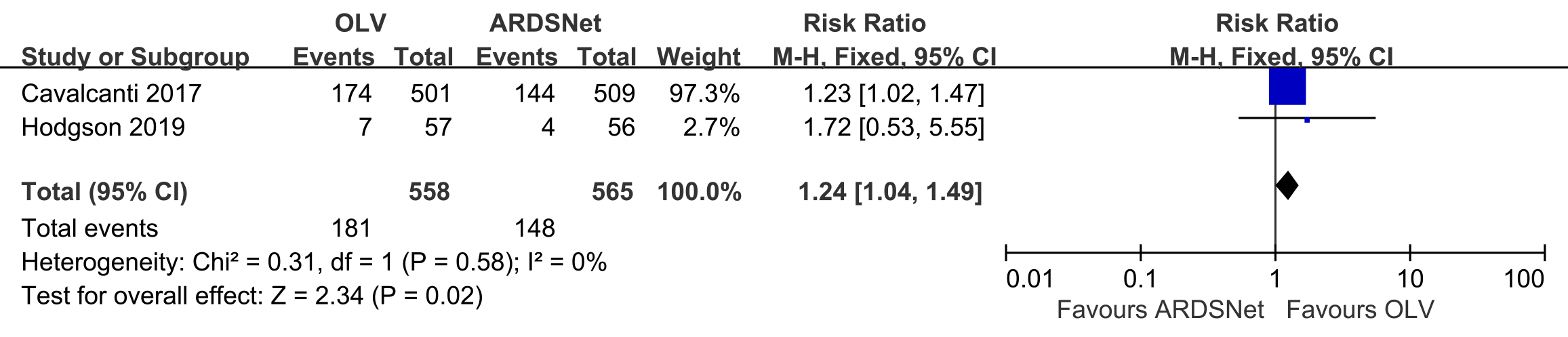

Supplement: Supplementary file 1 — Supplementary material [file mmc1.zip › Supplementary information/Supplementary information/Figure. S12.tif]

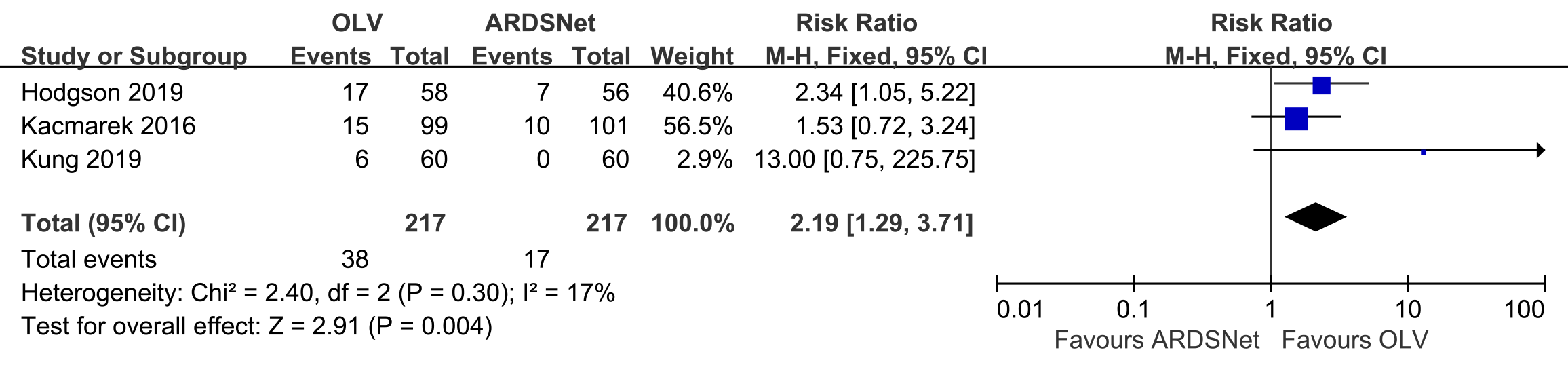

Supplement: Supplementary file 1 — Supplementary material [file mmc1.zip › Supplementary information/Supplementary information/Figure. S13a.tif]

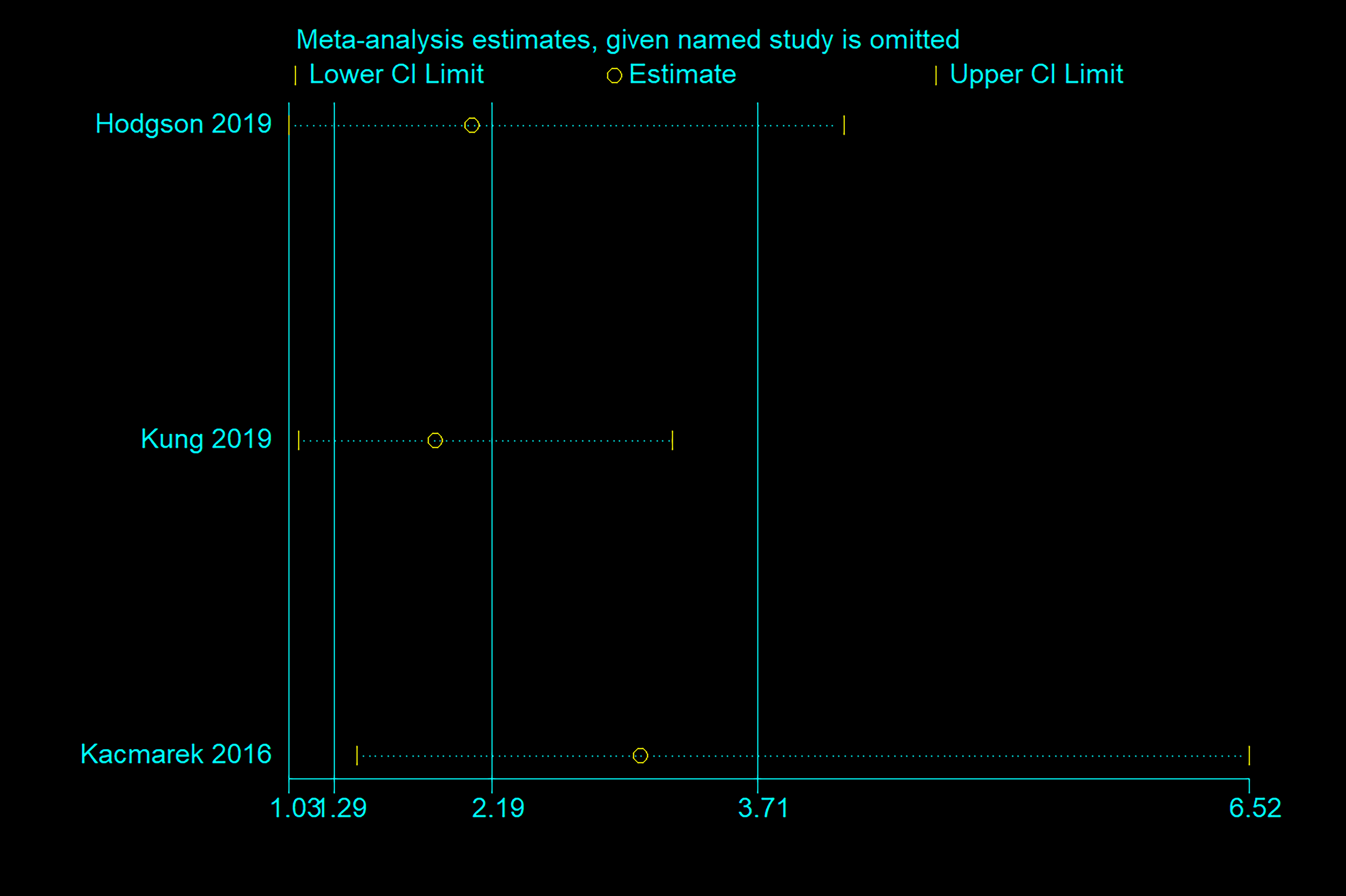

Supplement: Supplementary file 1 — Supplementary material [file mmc1.zip › Supplementary information/Supplementary information/Figure. S13b.tif]

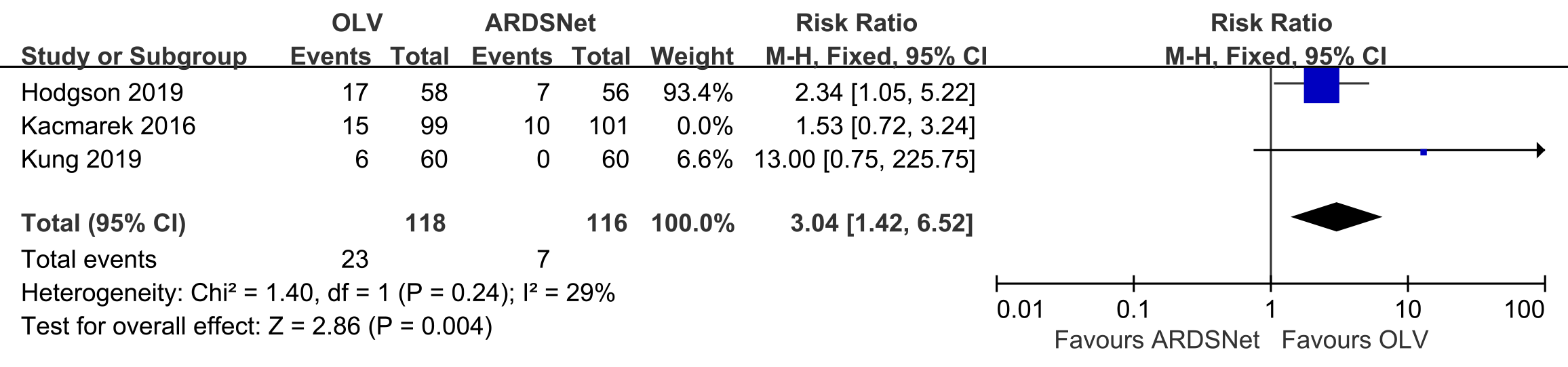

Supplement: Supplementary file 1 — Supplementary material [file mmc1.zip › Supplementary information/Supplementary information/Figure. S13c.tif]

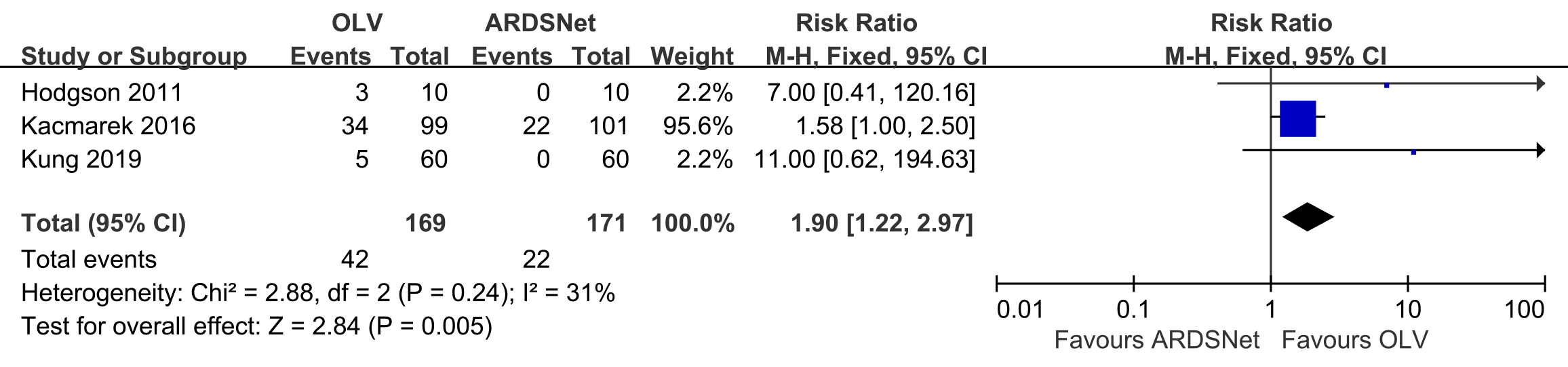

Supplement: Supplementary file 1 — Supplementary material [file mmc1.zip › Supplementary information/Supplementary information/Figure. S14a.tif]

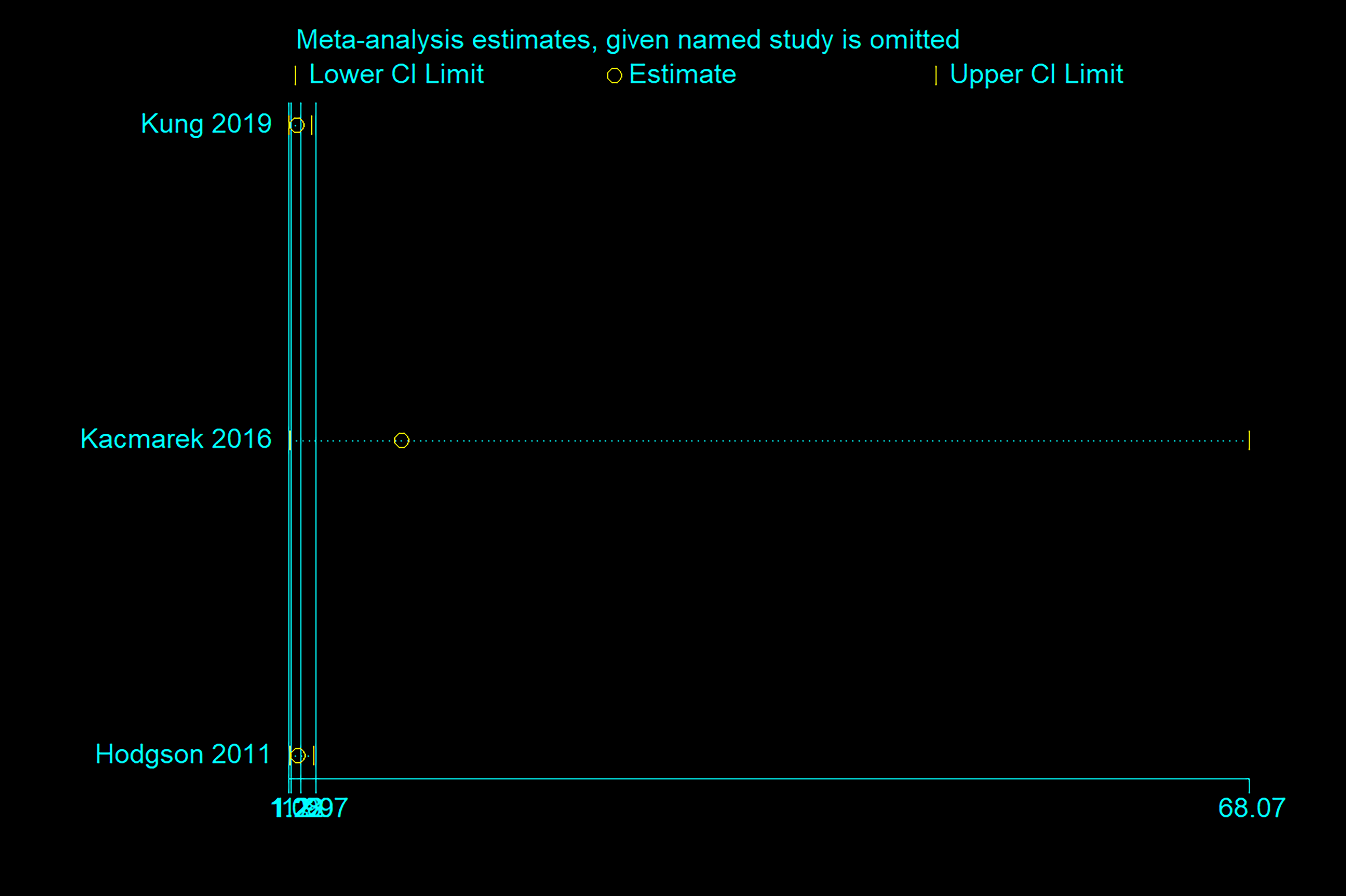

Supplement: Supplementary file 1 — Supplementary material [file mmc1.zip › Supplementary information/Supplementary information/Figure. S14b.tif]

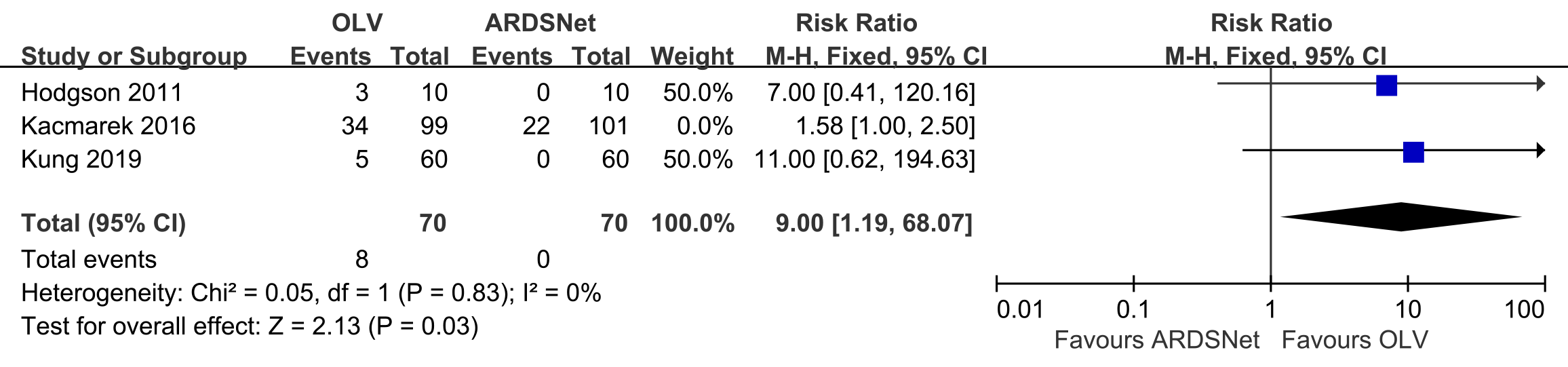

Supplement: Supplementary file 1 — Supplementary material [file mmc1.zip › Supplementary information/Supplementary information/Figure. S14c.tif]

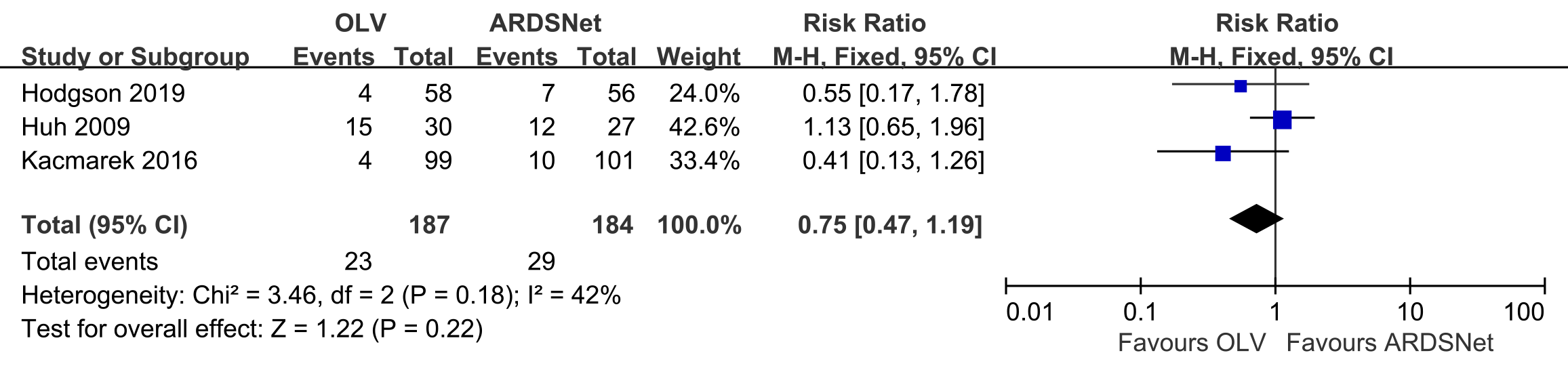

Supplement: Supplementary file 1 — Supplementary material [file mmc1.zip › Supplementary information/Supplementary information/Figure. S15a.tif]

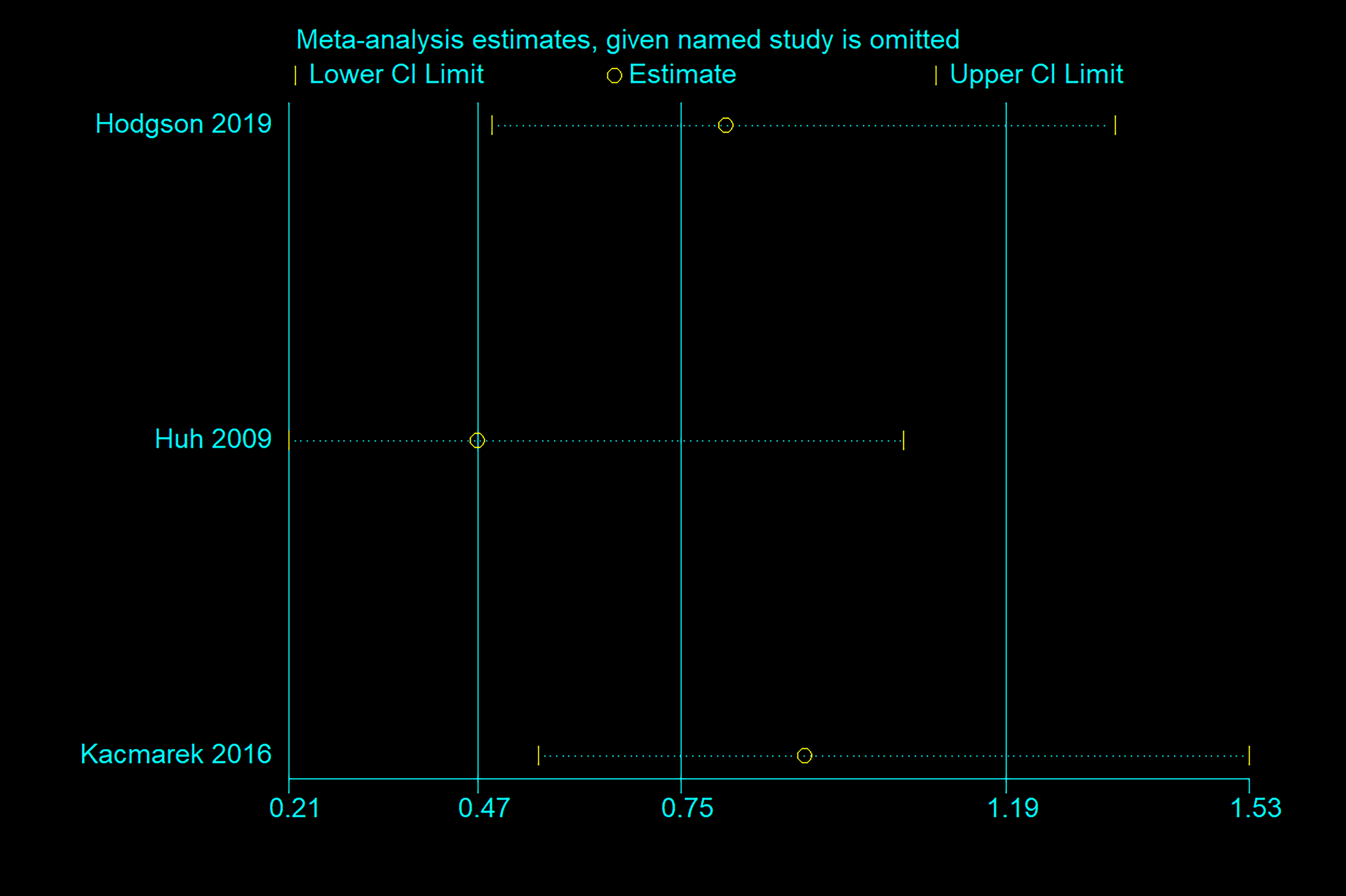

Supplement: Supplementary file 1 — Supplementary material [file mmc1.zip › Supplementary information/Supplementary information/Figure. S15b.tif]

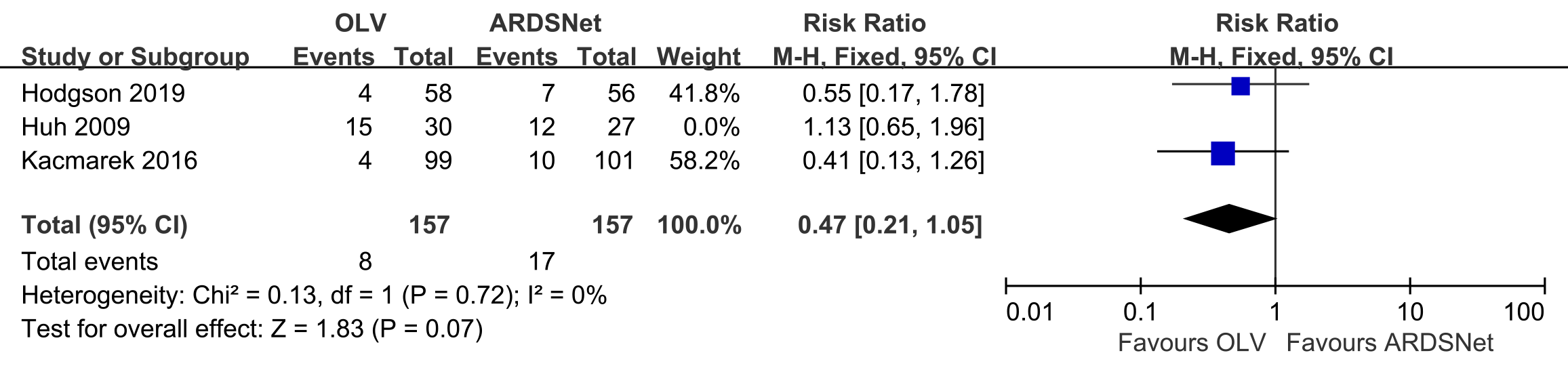

Supplement: Supplementary file 1 — Supplementary material [file mmc1.zip › Supplementary information/Supplementary information/Figure. S15c.tif]

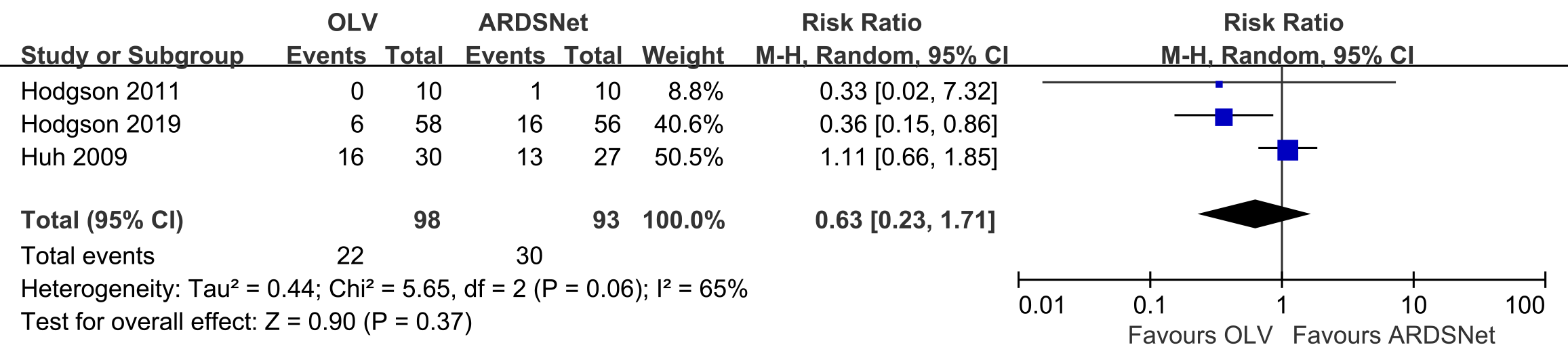

Supplement: Supplementary file 1 — Supplementary material [file mmc1.zip › Supplementary information/Supplementary information/Figure. S16a.tif]

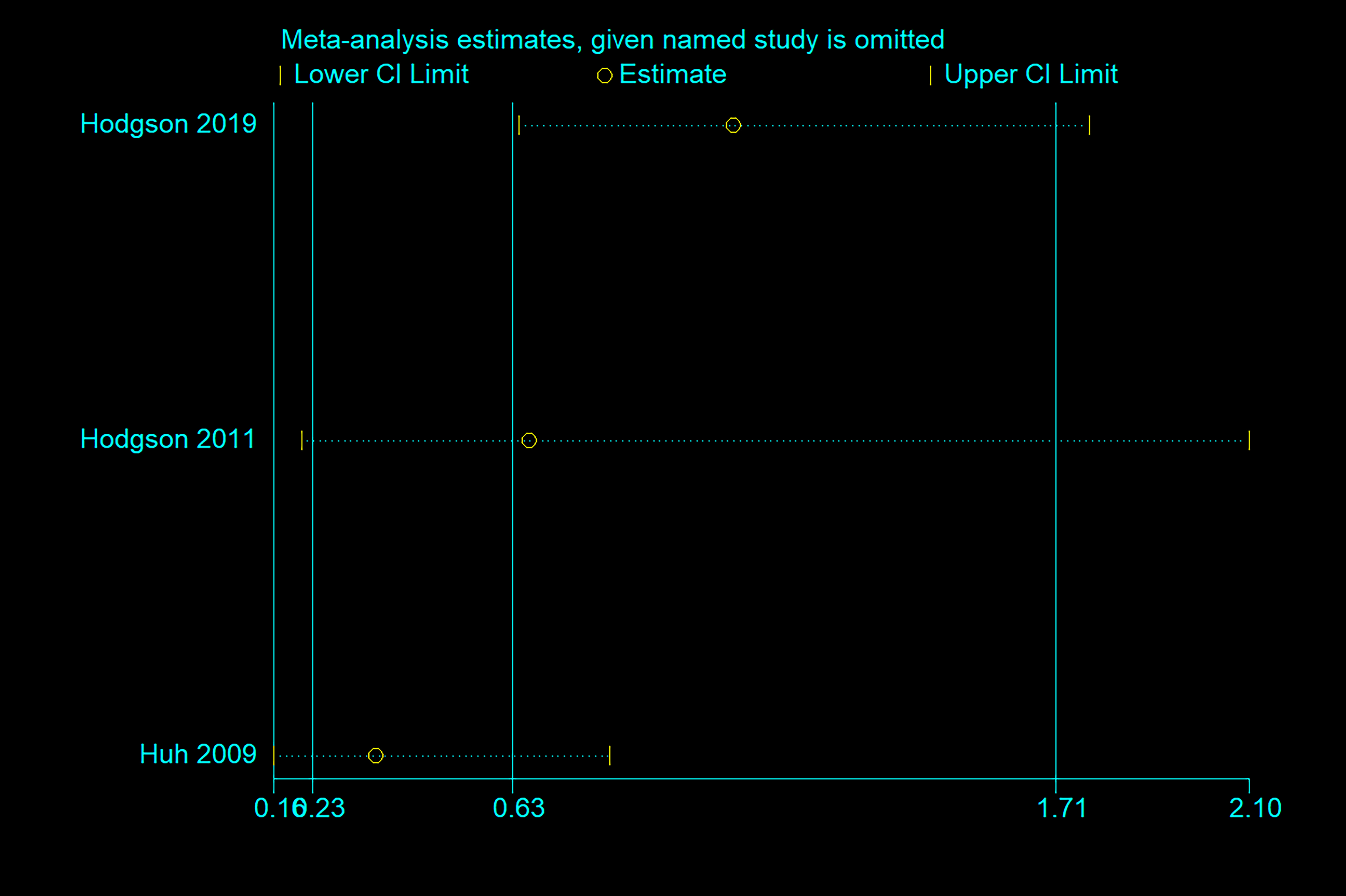

Supplement: Supplementary file 1 — Supplementary material [file mmc1.zip › Supplementary information/Supplementary information/Figure. S16b.tif]

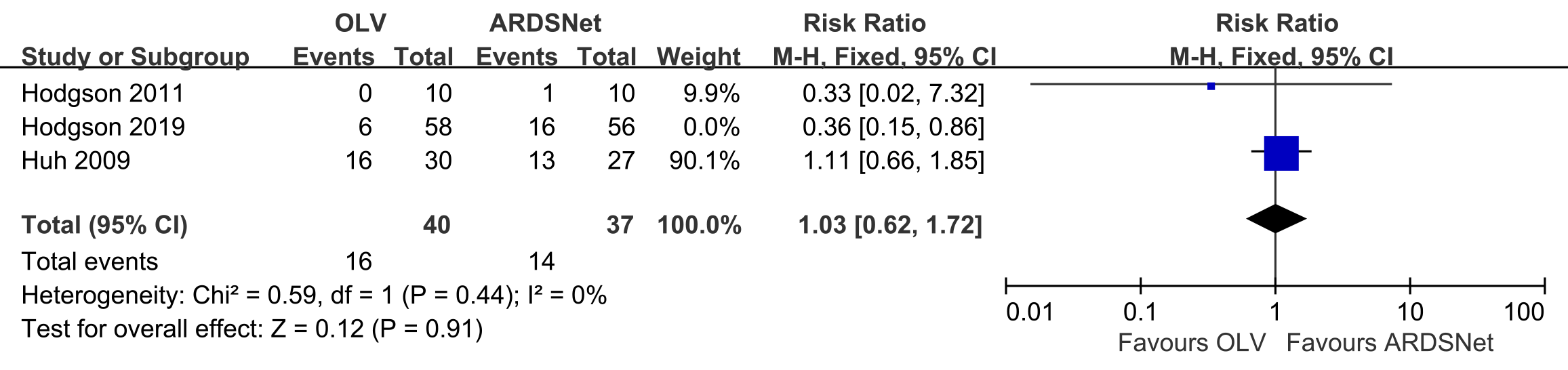

Supplement: Supplementary file 1 — Supplementary material [file mmc1.zip › Supplementary information/Supplementary information/Figure. S16c.tif]

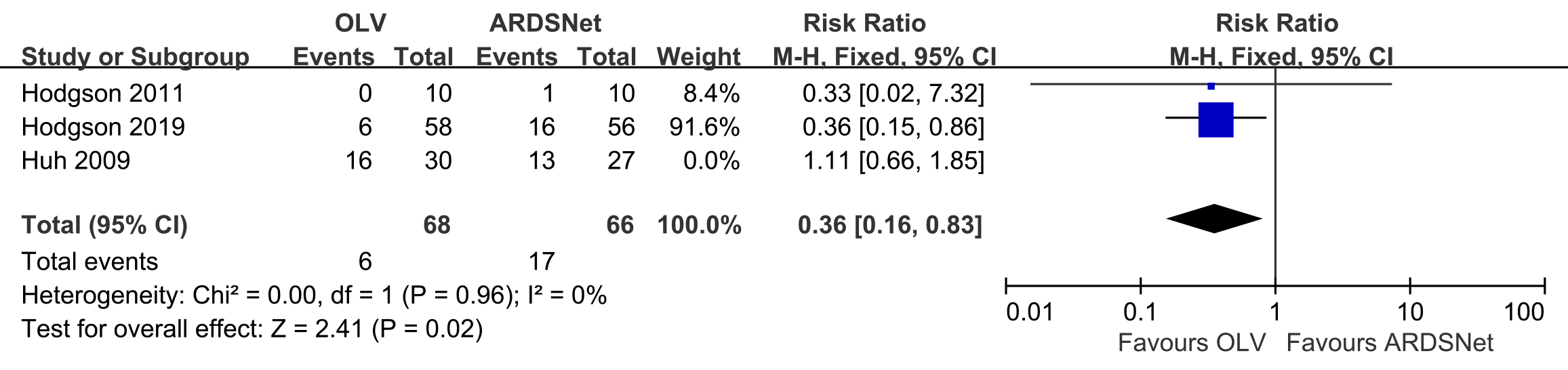

Supplement: Supplementary file 1 — Supplementary material [file mmc1.zip › Supplementary information/Supplementary information/Figure. S16d.tif]

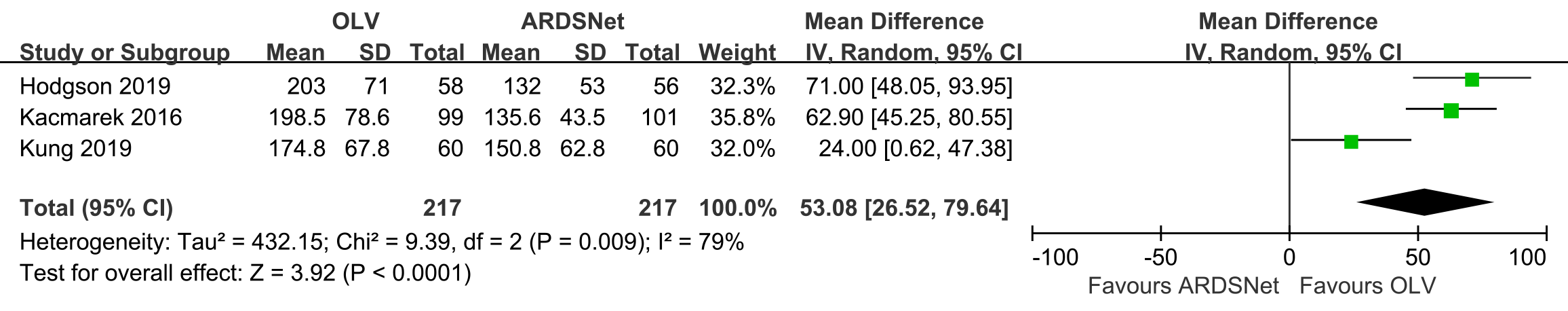

Supplement: Supplementary file 1 — Supplementary material [file mmc1.zip › Supplementary information/Supplementary information/Figure. S17a.tif]

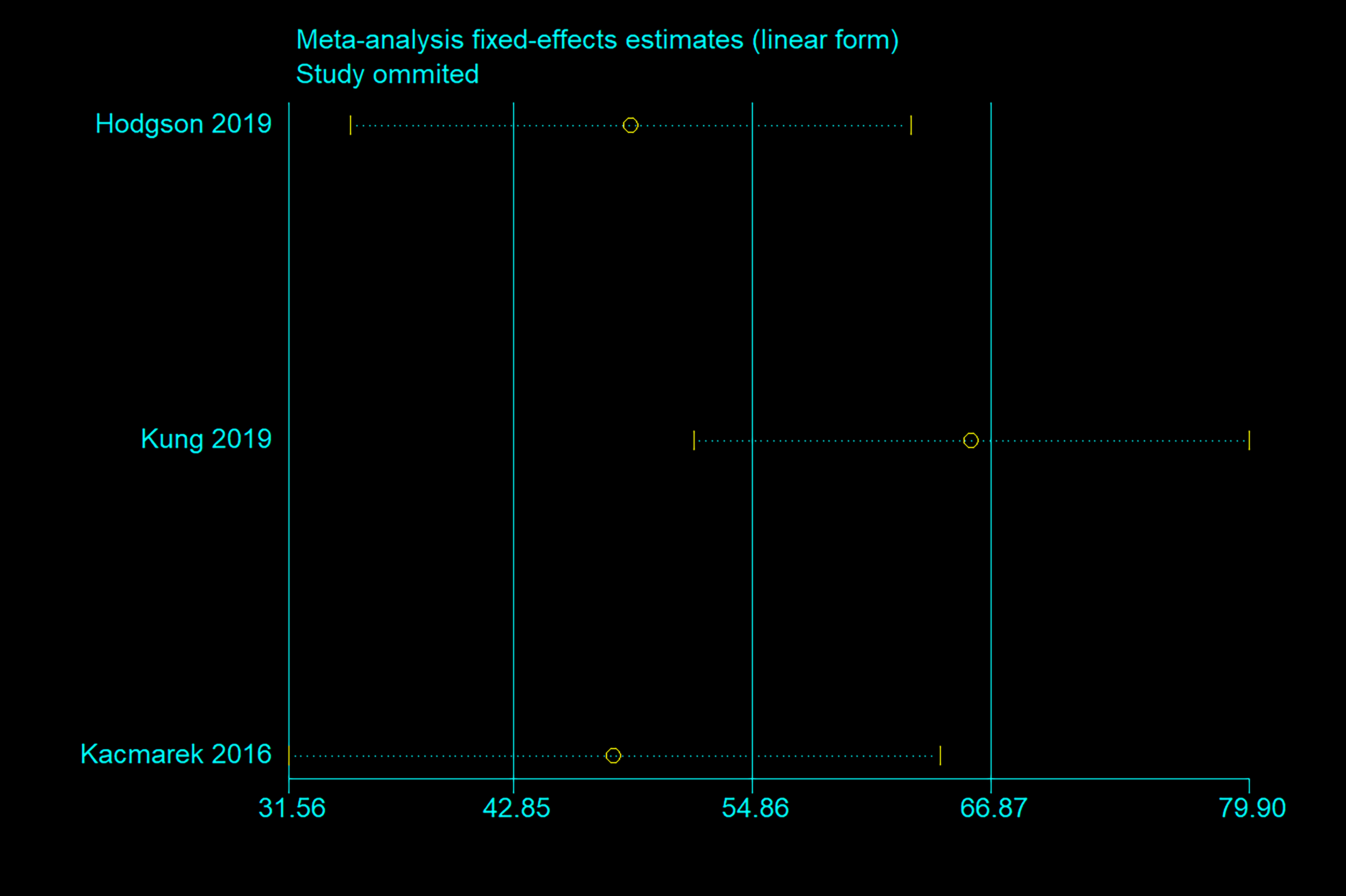

Supplement: Supplementary file 1 — Supplementary material [file mmc1.zip › Supplementary information/Supplementary information/Figure. S17b.tif]

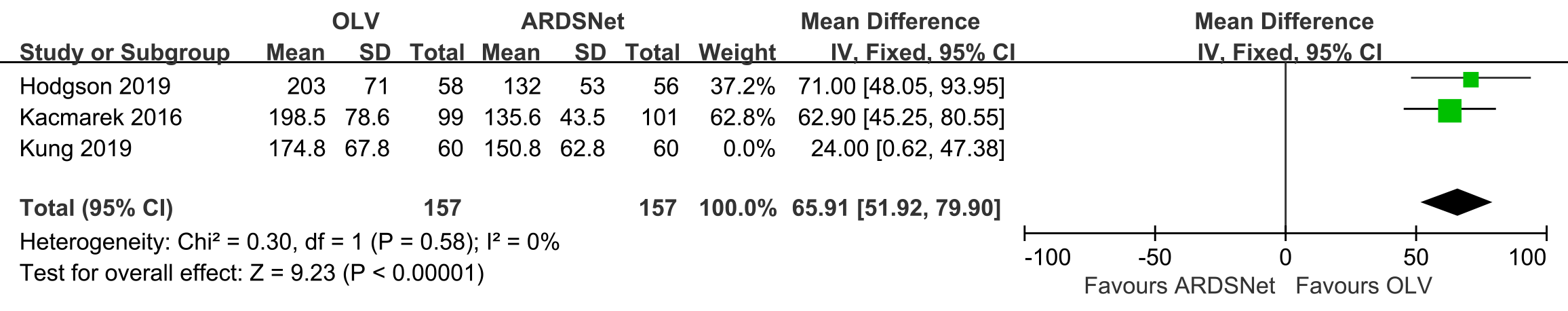

Supplement: Supplementary file 1 — Supplementary material [file mmc1.zip › Supplementary information/Supplementary information/Figure. S17c.tif]

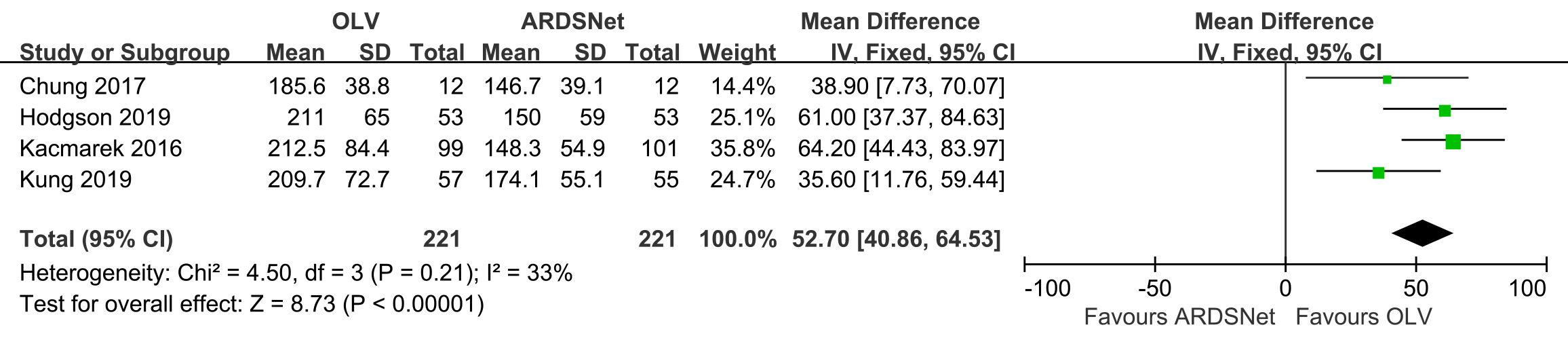

Supplement: Supplementary file 1 — Supplementary material [file mmc1.zip › Supplementary information/Supplementary information/Figure. S18a.tif]

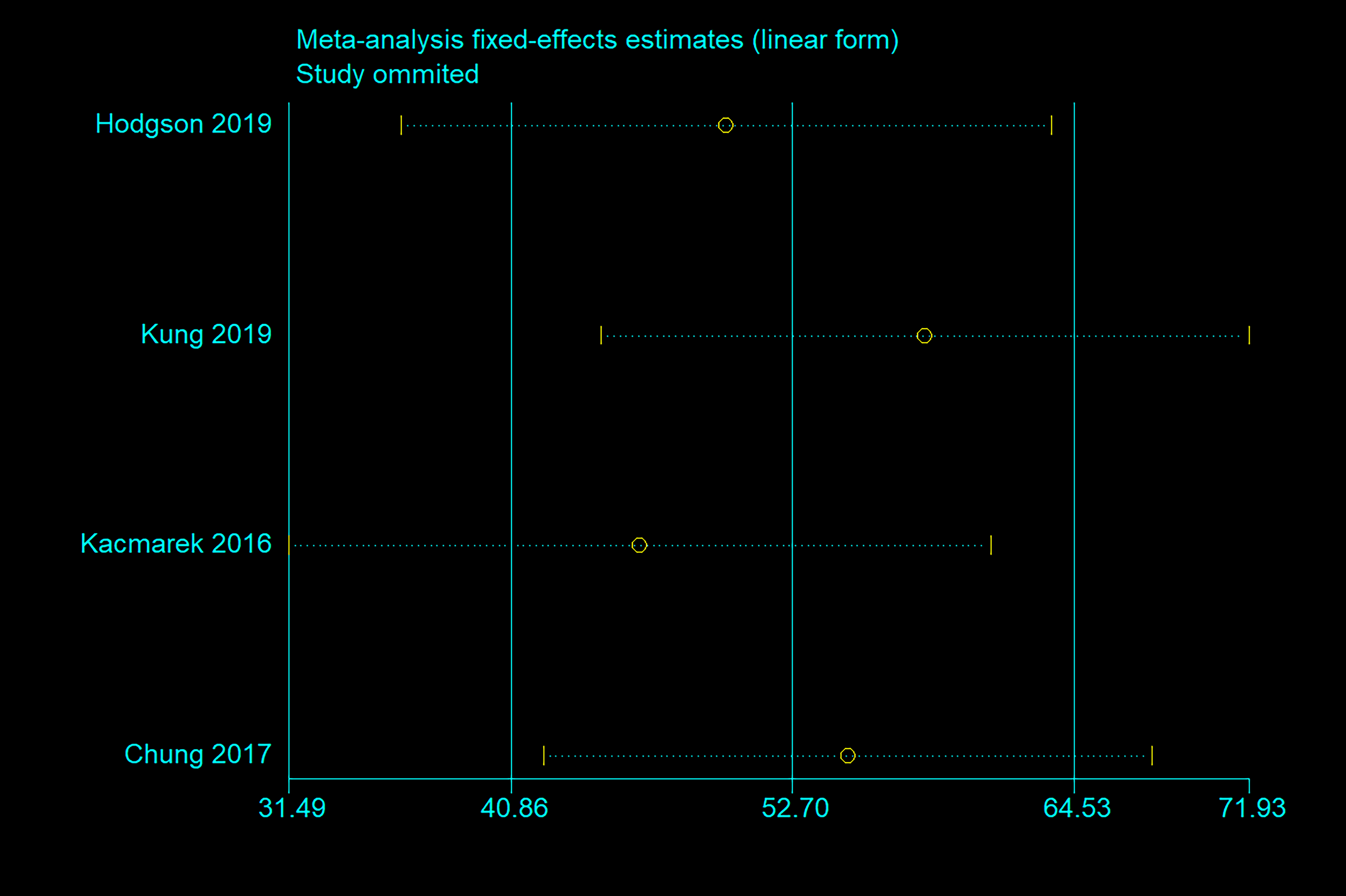

Supplement: Supplementary file 1 — Supplementary material [file mmc1.zip › Supplementary information/Supplementary information/Figure. S18b.tif]

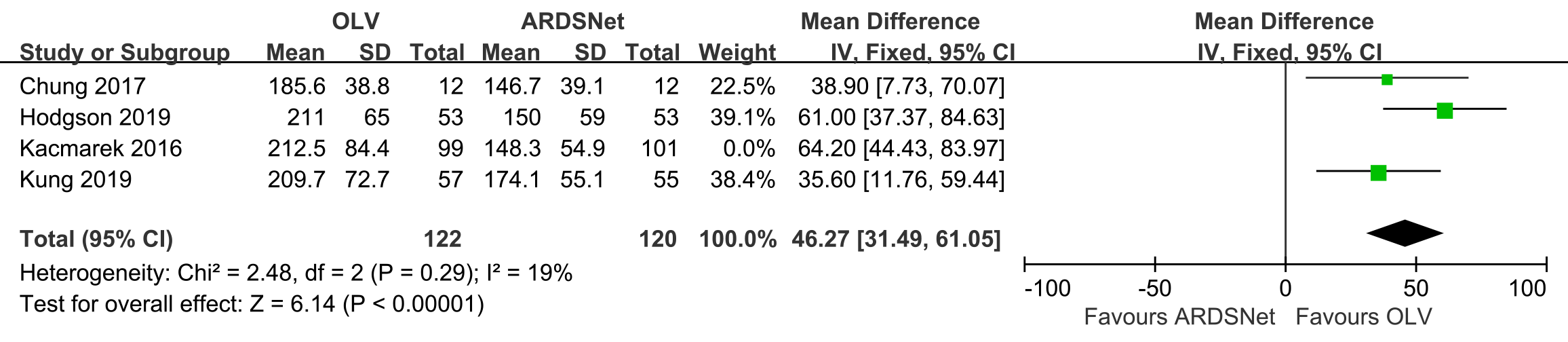

Supplement: Supplementary file 1 — Supplementary material [file mmc1.zip › Supplementary information/Supplementary information/Figure. S18c.tif]

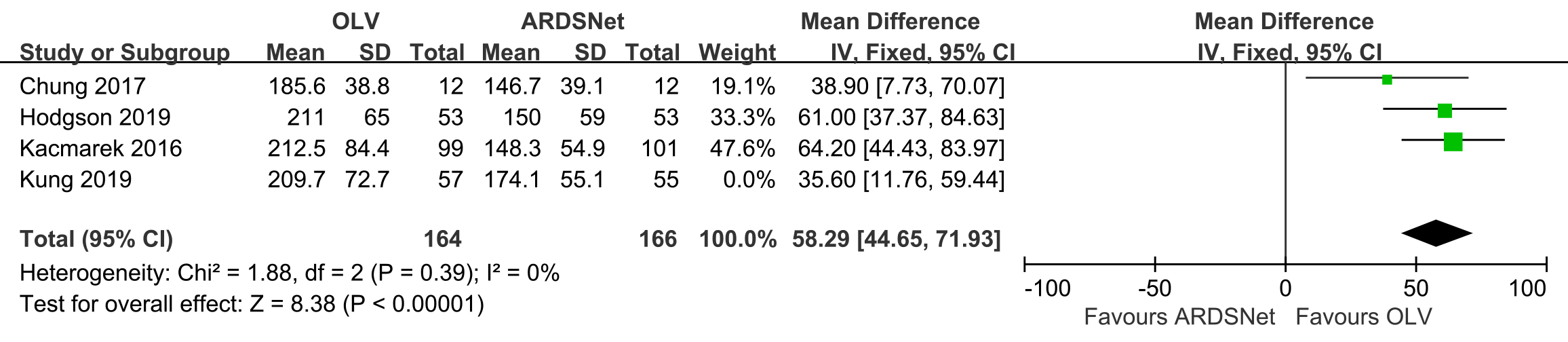

Supplement: Supplementary file 1 — Supplementary material [file mmc1.zip › Supplementary information/Supplementary information/Figure. S18d.tif]

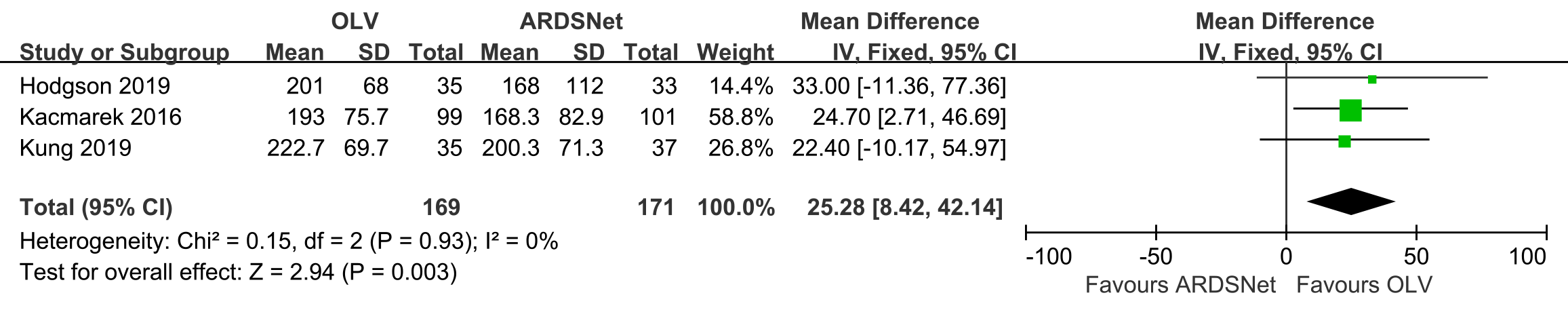

Supplement: Supplementary file 1 — Supplementary material [file mmc1.zip › Supplementary information/Supplementary information/Figure. S19a.tif]

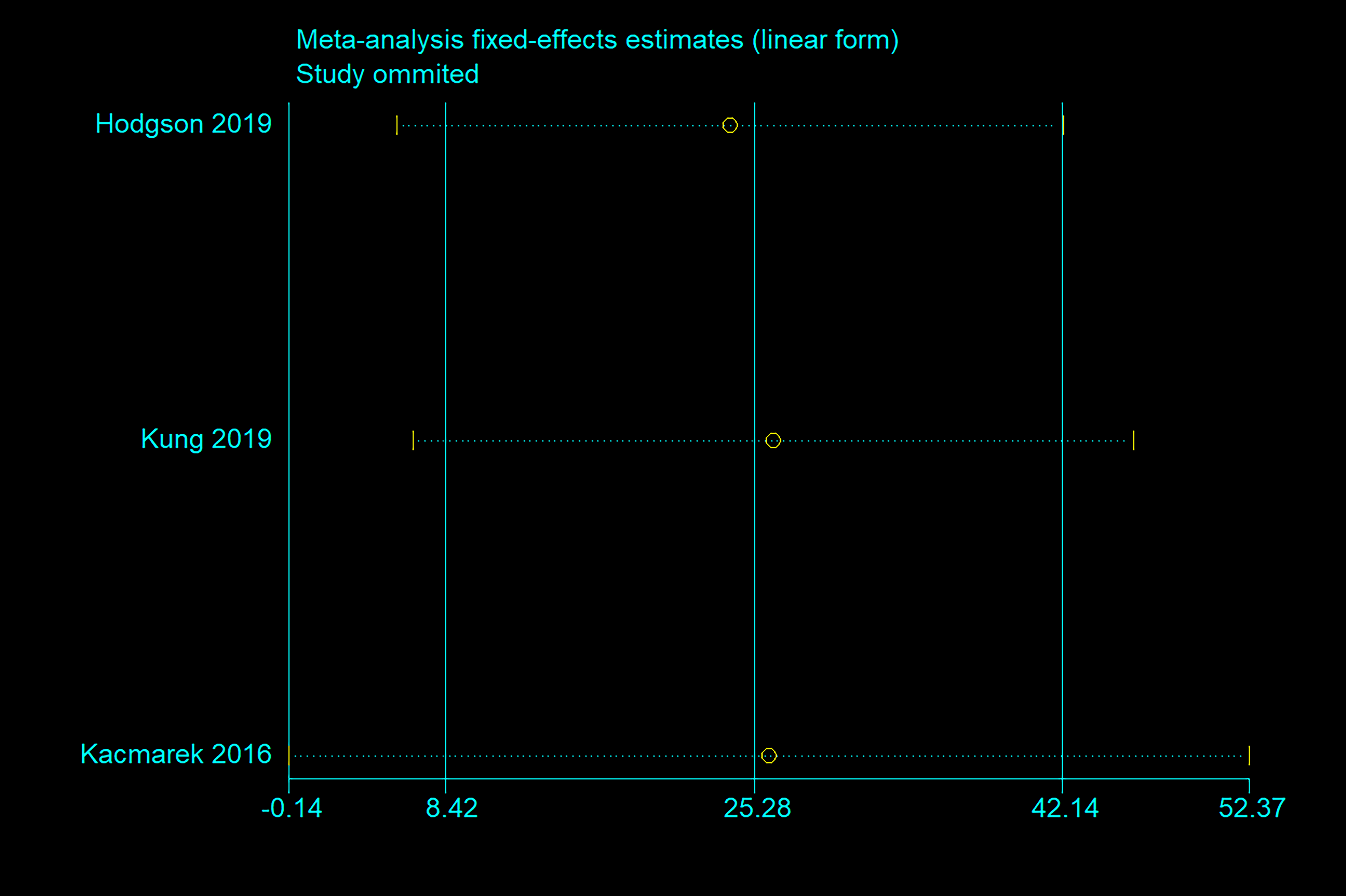

Supplement: Supplementary file 1 — Supplementary material [file mmc1.zip › Supplementary information/Supplementary information/Figure. S19b.tif]

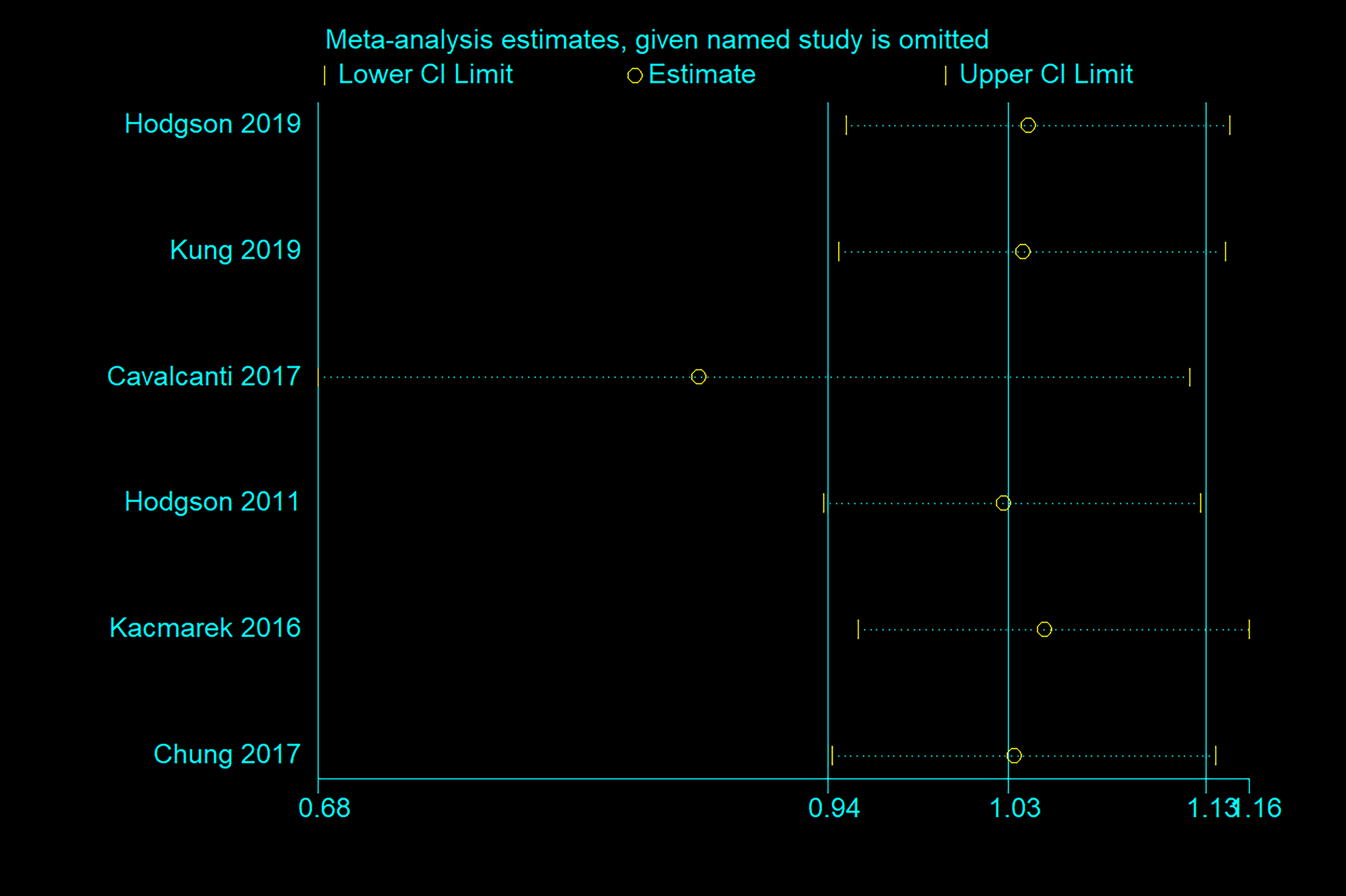

Supplement: Supplementary file 1 — Supplementary material [file mmc1.zip › Supplementary information/Supplementary information/Figure. S3a.tif]

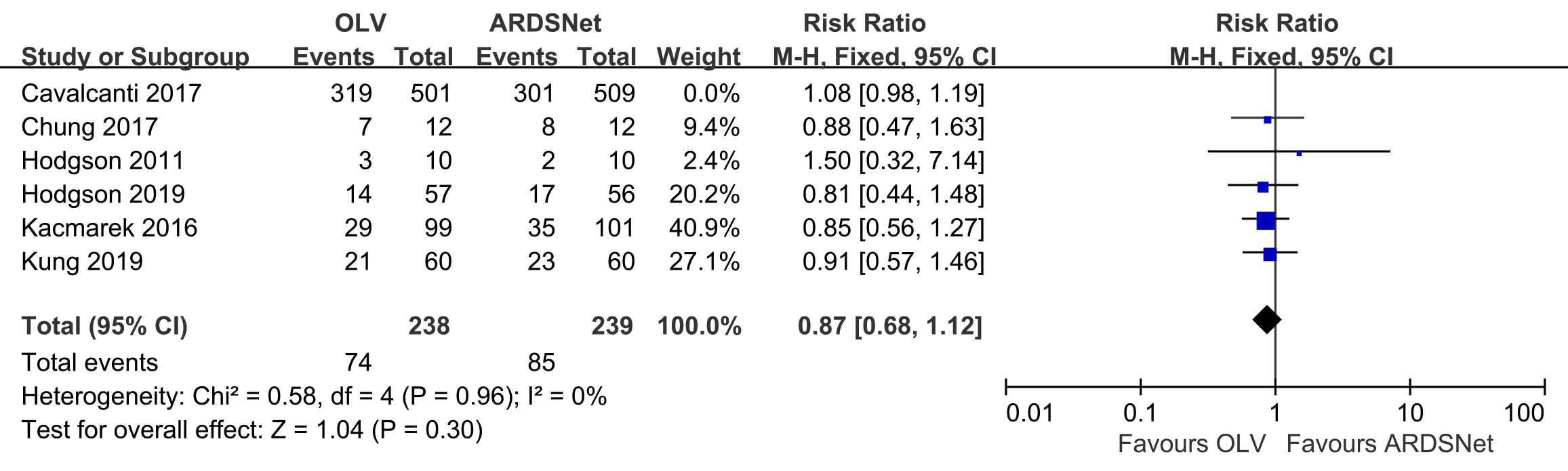

Supplement: Supplementary file 1 — Supplementary material [file mmc1.zip › Supplementary information/Supplementary information/Figure. S3b.tif]

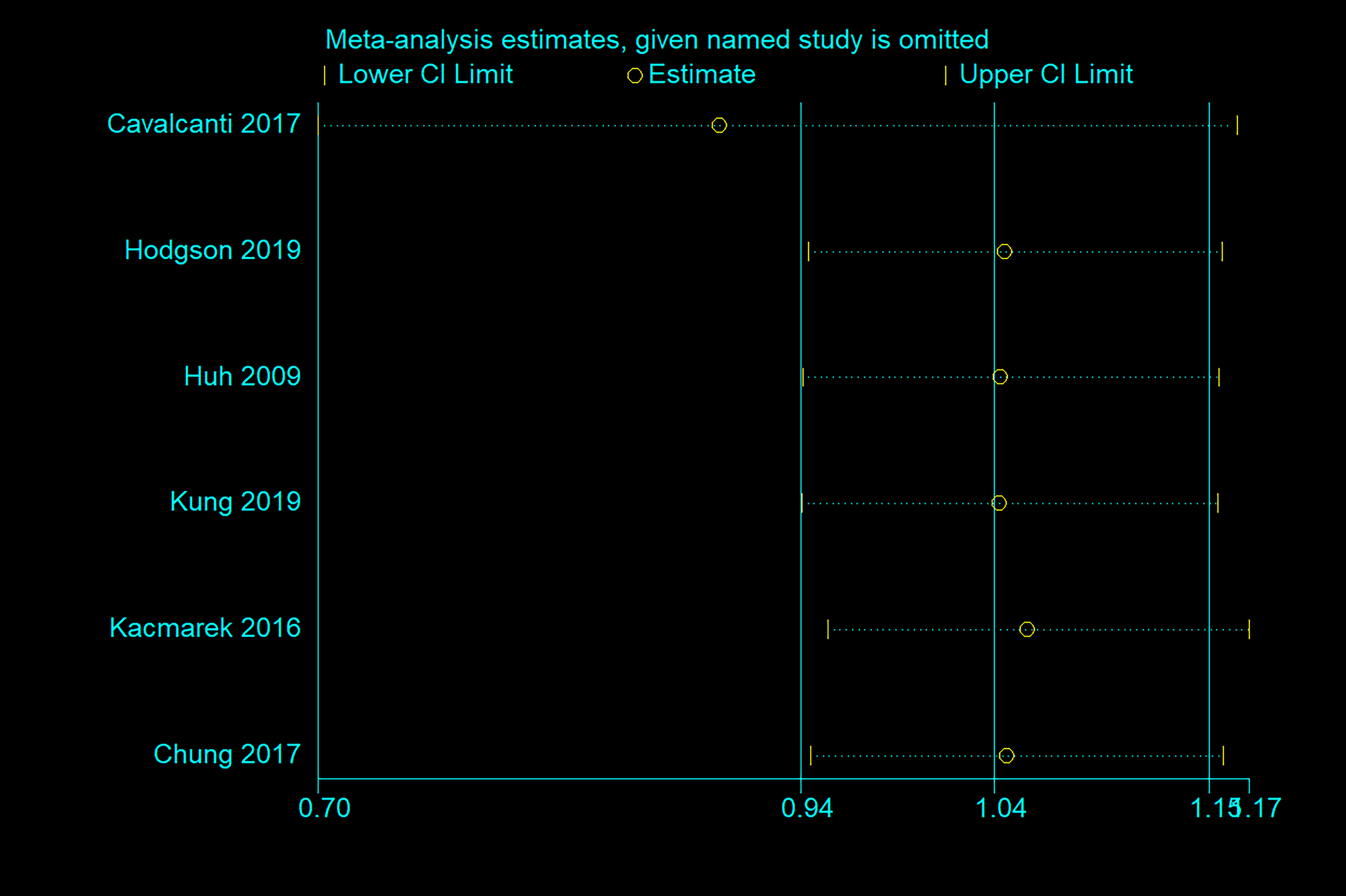

Supplement: Supplementary file 1 — Supplementary material [file mmc1.zip › Supplementary information/Supplementary information/Figure. S4a.tif]

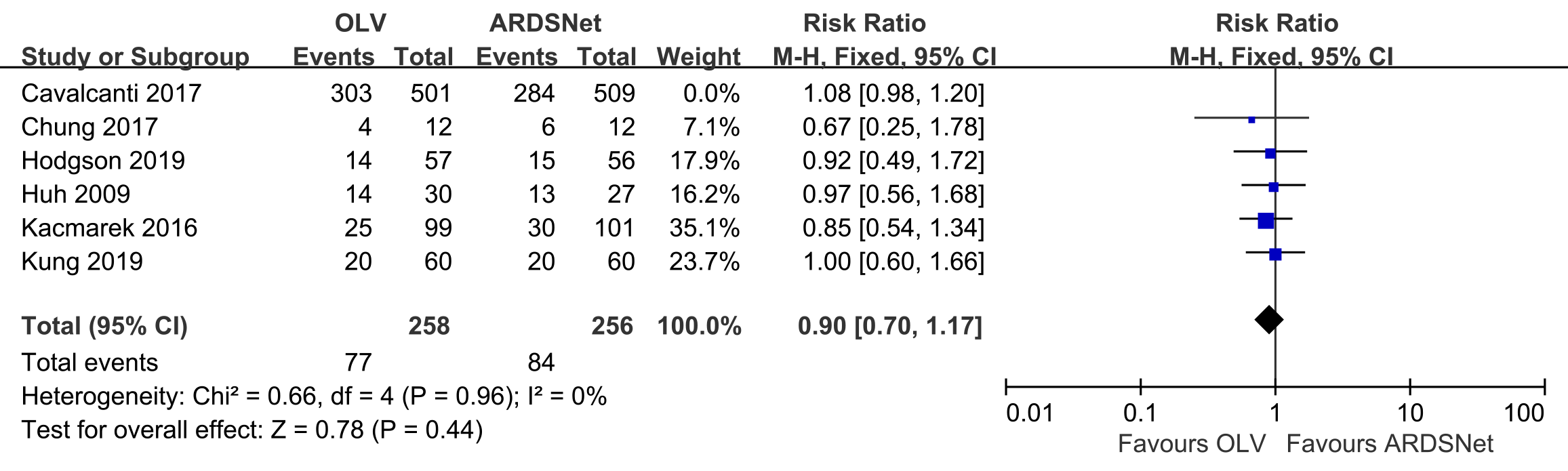

Supplement: Supplementary file 1 — Supplementary material [file mmc1.zip › Supplementary information/Supplementary information/Figure. S4b.tif]

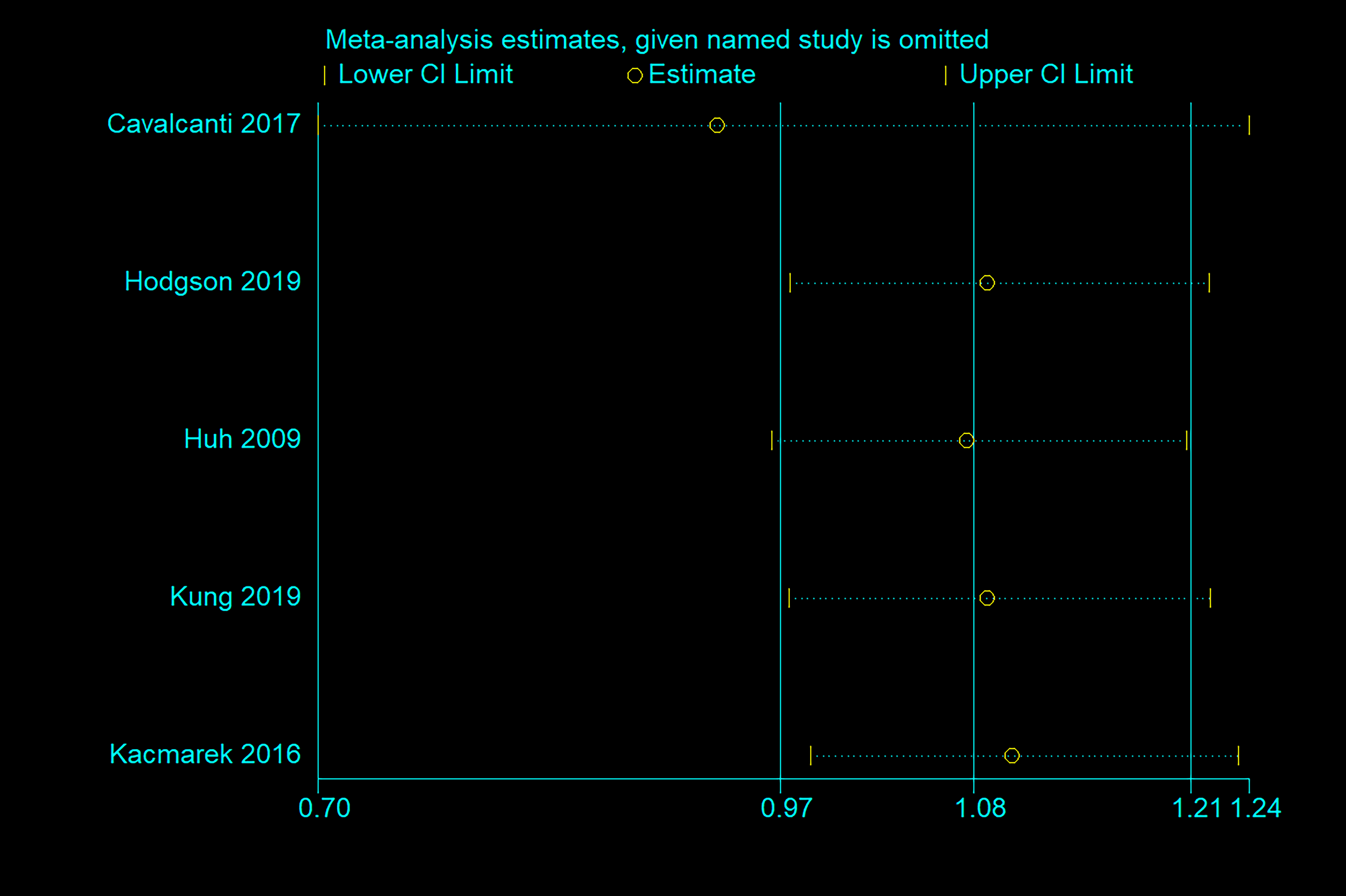

Supplement: Supplementary file 1 — Supplementary material [file mmc1.zip › Supplementary information/Supplementary information/Figure. S5a.tif]

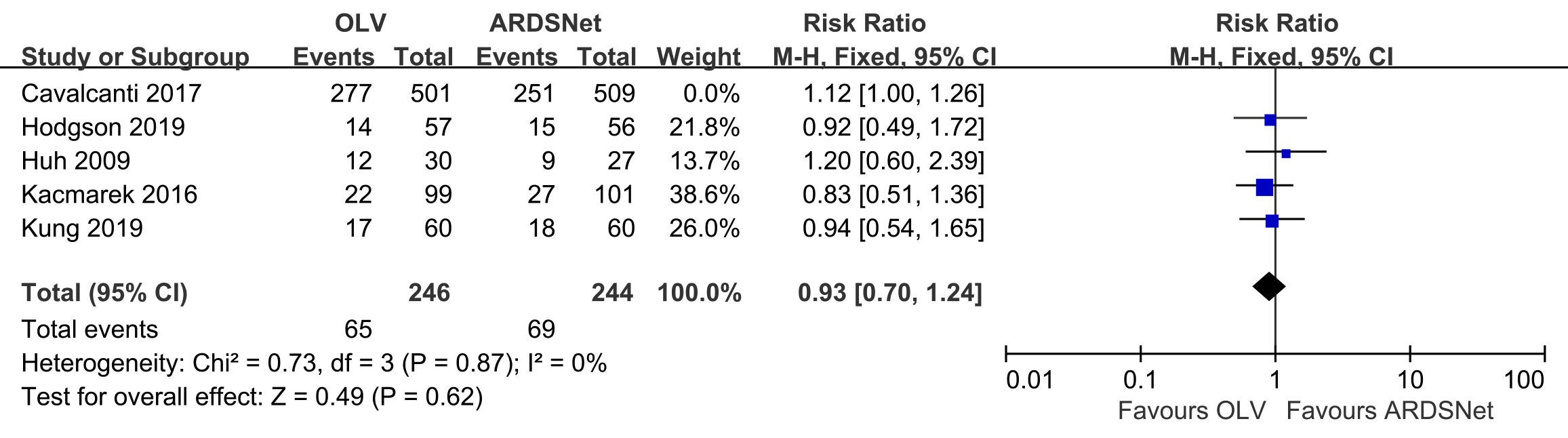

Supplement: Supplementary file 1 — Supplementary material [file mmc1.zip › Supplementary information/Supplementary information/Figure. S5b.tif]

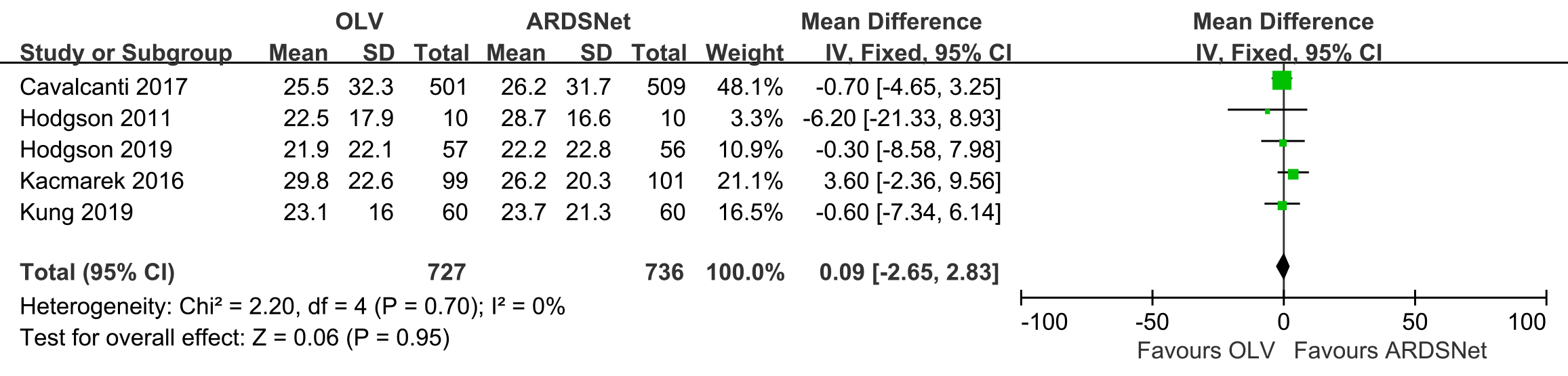

Supplement: Supplementary file 1 — Supplementary material [file mmc1.zip › Supplementary information/Supplementary information/Figure. S6a.tif]

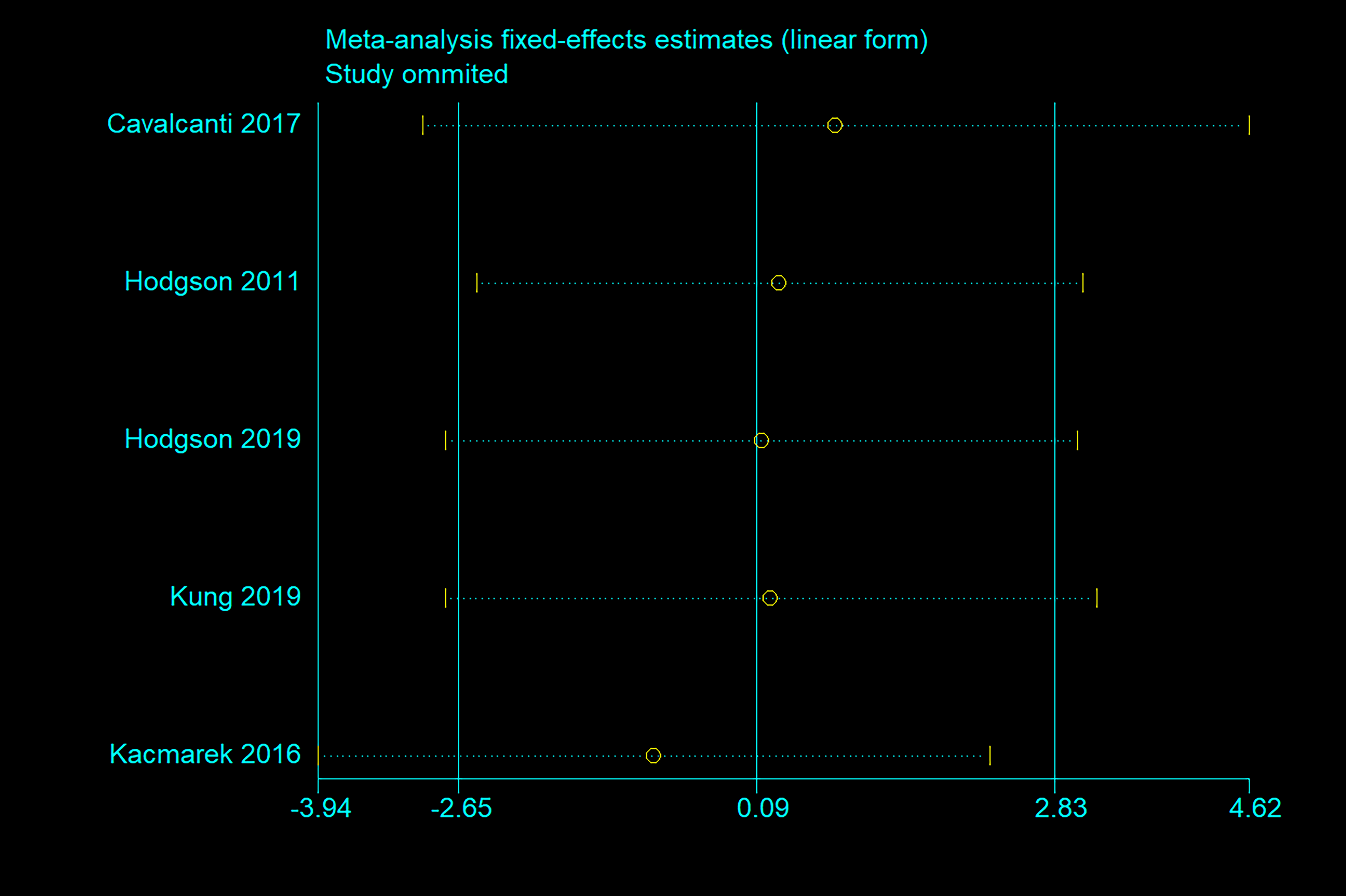

Supplement: Supplementary file 1 — Supplementary material [file mmc1.zip › Supplementary information/Supplementary information/Figure. S6b.tif]

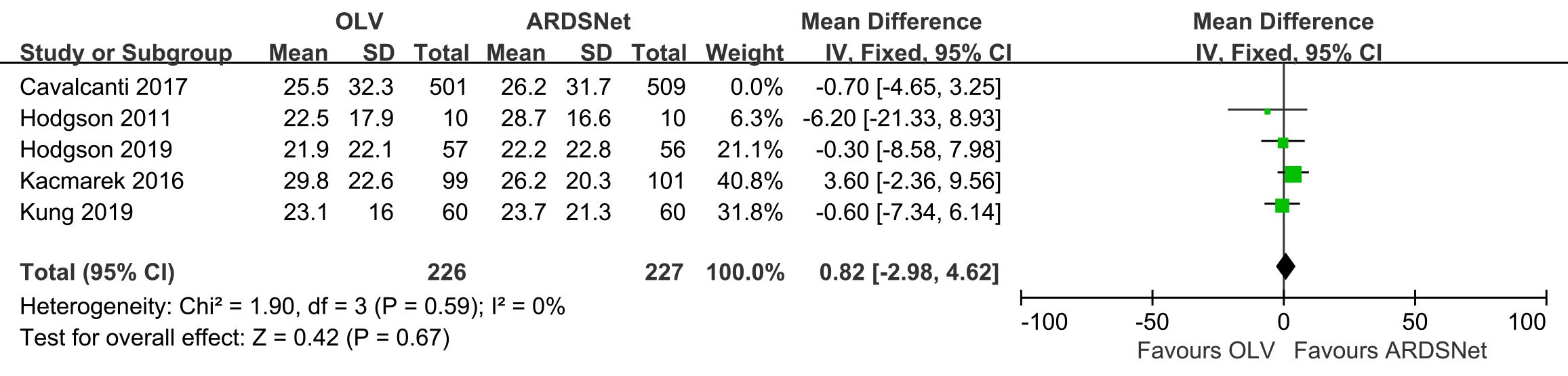

Supplement: Supplementary file 1 — Supplementary material [file mmc1.zip › Supplementary information/Supplementary information/Figure. S6c.tif]

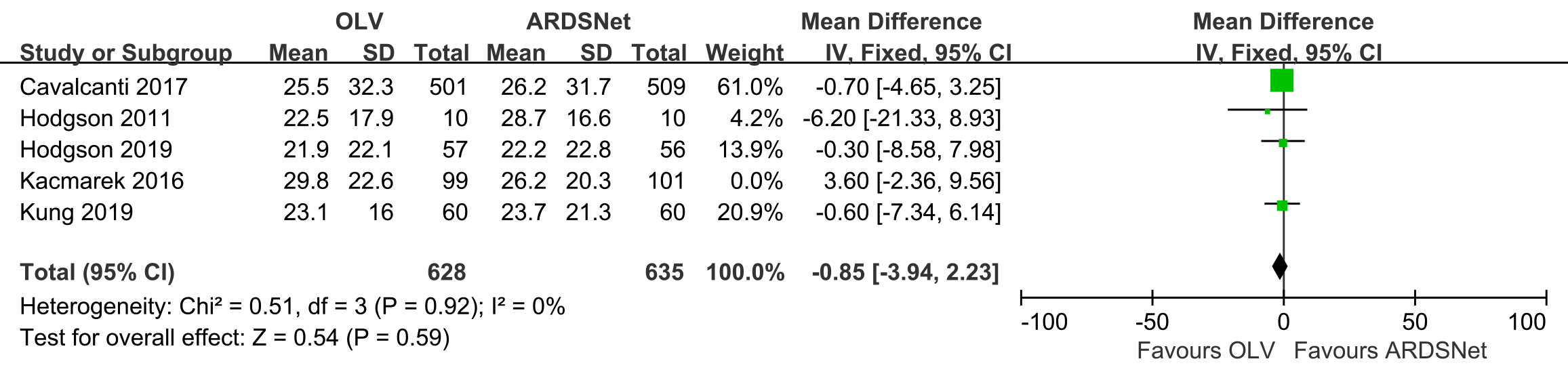

Supplement: Supplementary file 1 — Supplementary material [file mmc1.zip › Supplementary information/Supplementary information/Figure. S6d.tif]

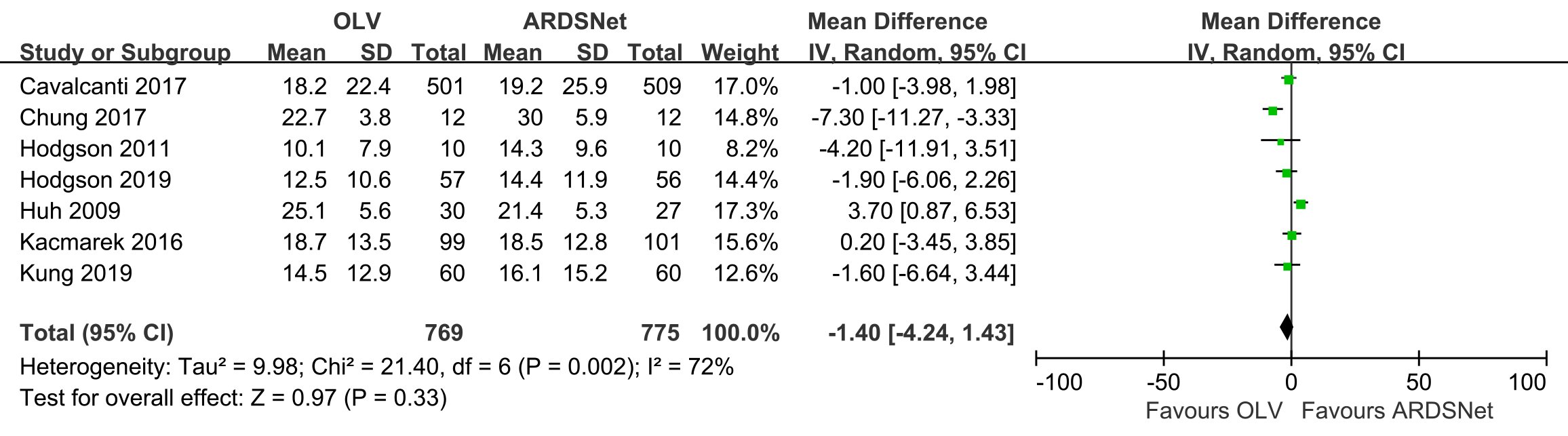

Supplement: Supplementary file 1 — Supplementary material [file mmc1.zip › Supplementary information/Supplementary information/Figure. S7a.tif]

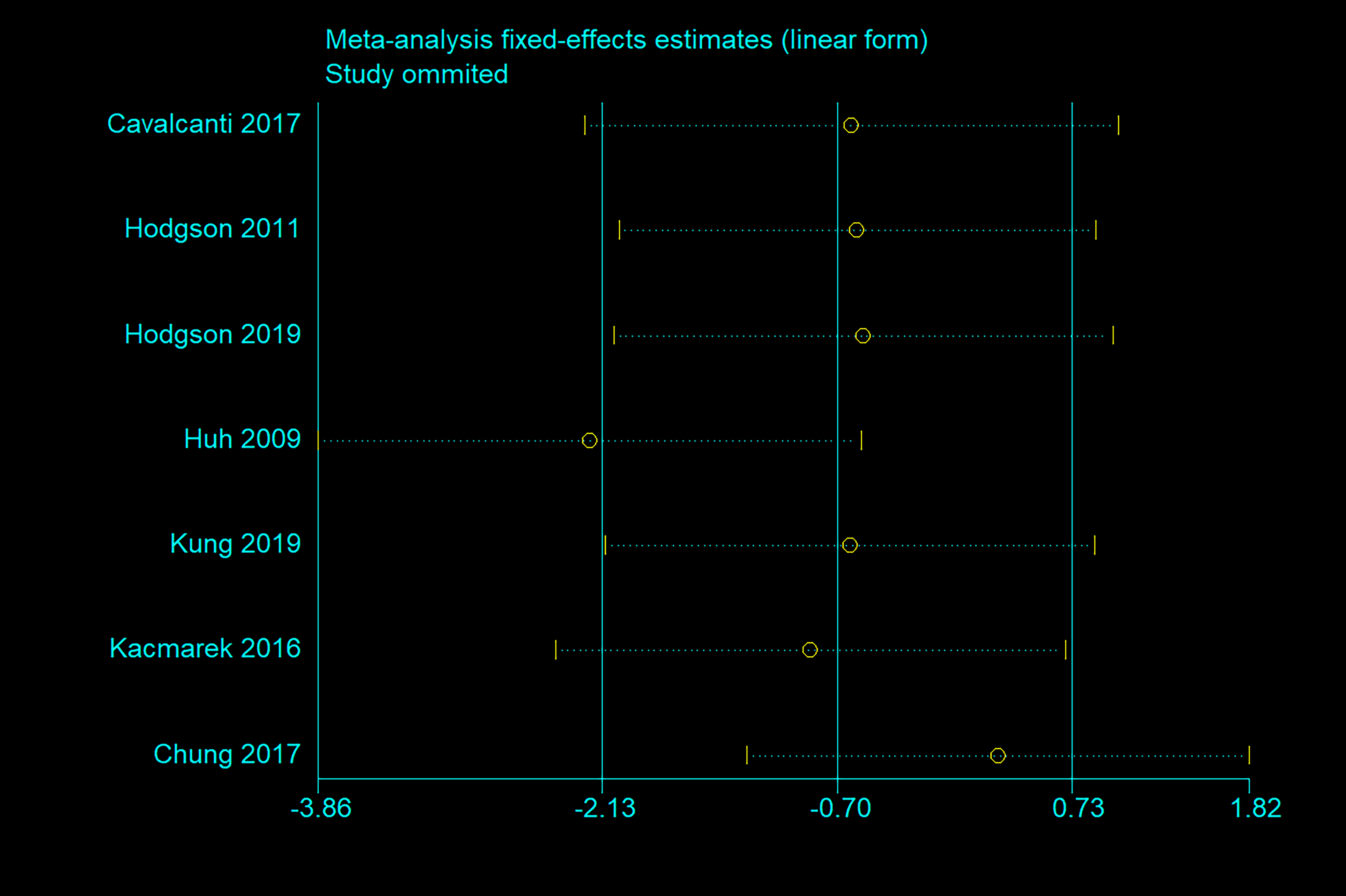

Supplement: Supplementary file 1 — Supplementary material [file mmc1.zip › Supplementary information/Supplementary information/Figure. S7b.tif]

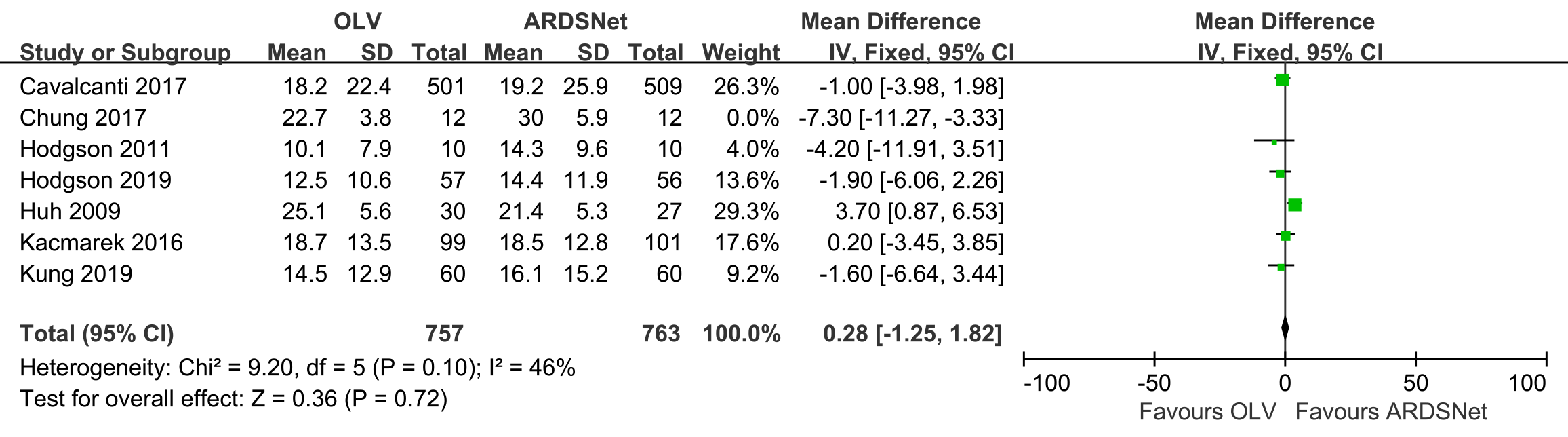

Supplement: Supplementary file 1 — Supplementary material [file mmc1.zip › Supplementary information/Supplementary information/Figure. S7c.tif]

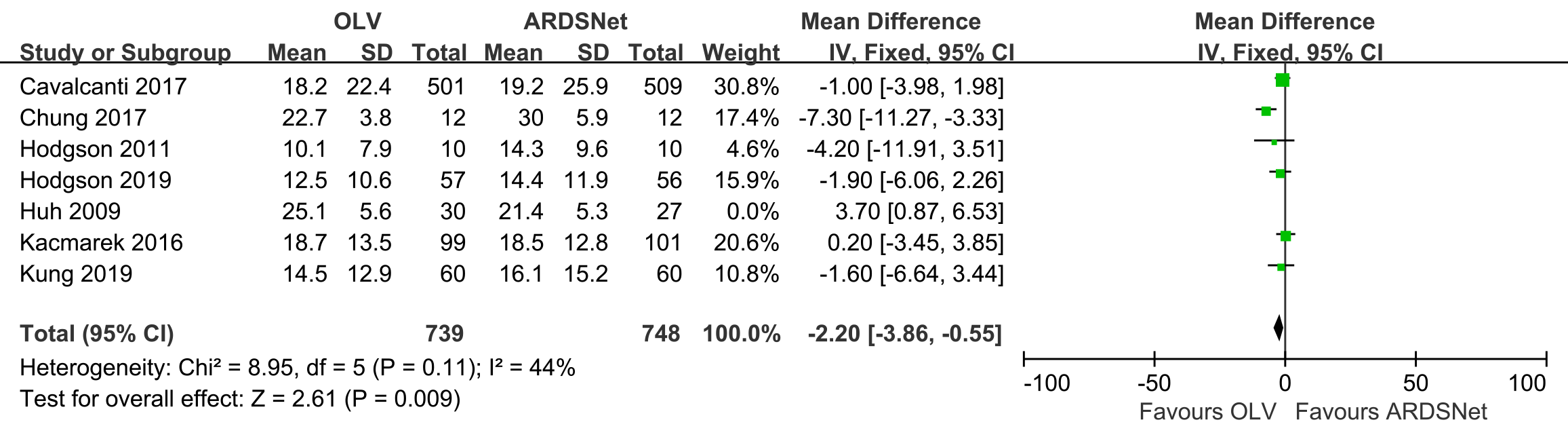

Supplement: Supplementary file 1 — Supplementary material [file mmc1.zip › Supplementary information/Supplementary information/Figure. S7d.tif]

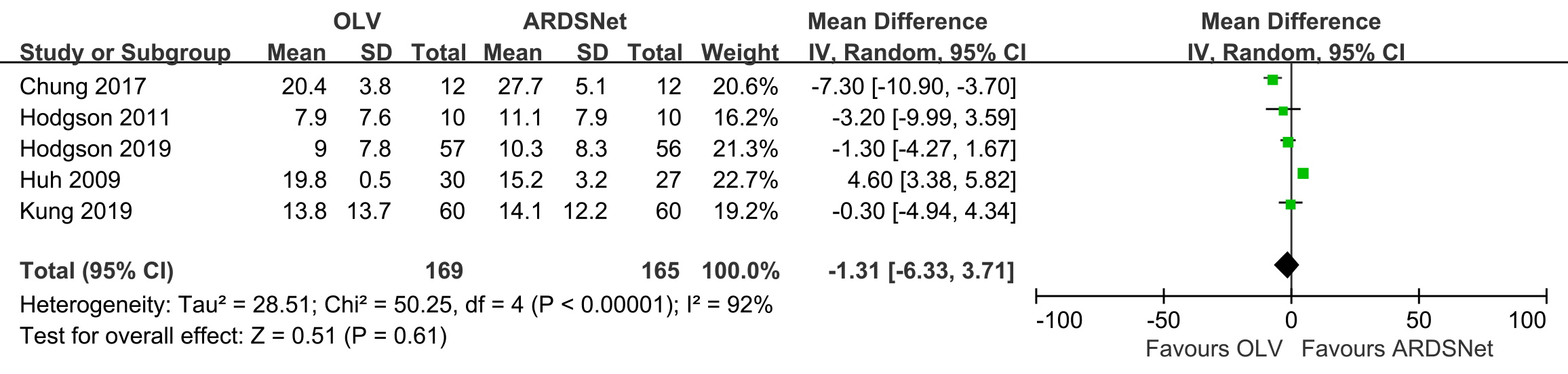

Supplement: Supplementary file 1 — Supplementary material [file mmc1.zip › Supplementary information/Supplementary information/Figure. S8a.tif]

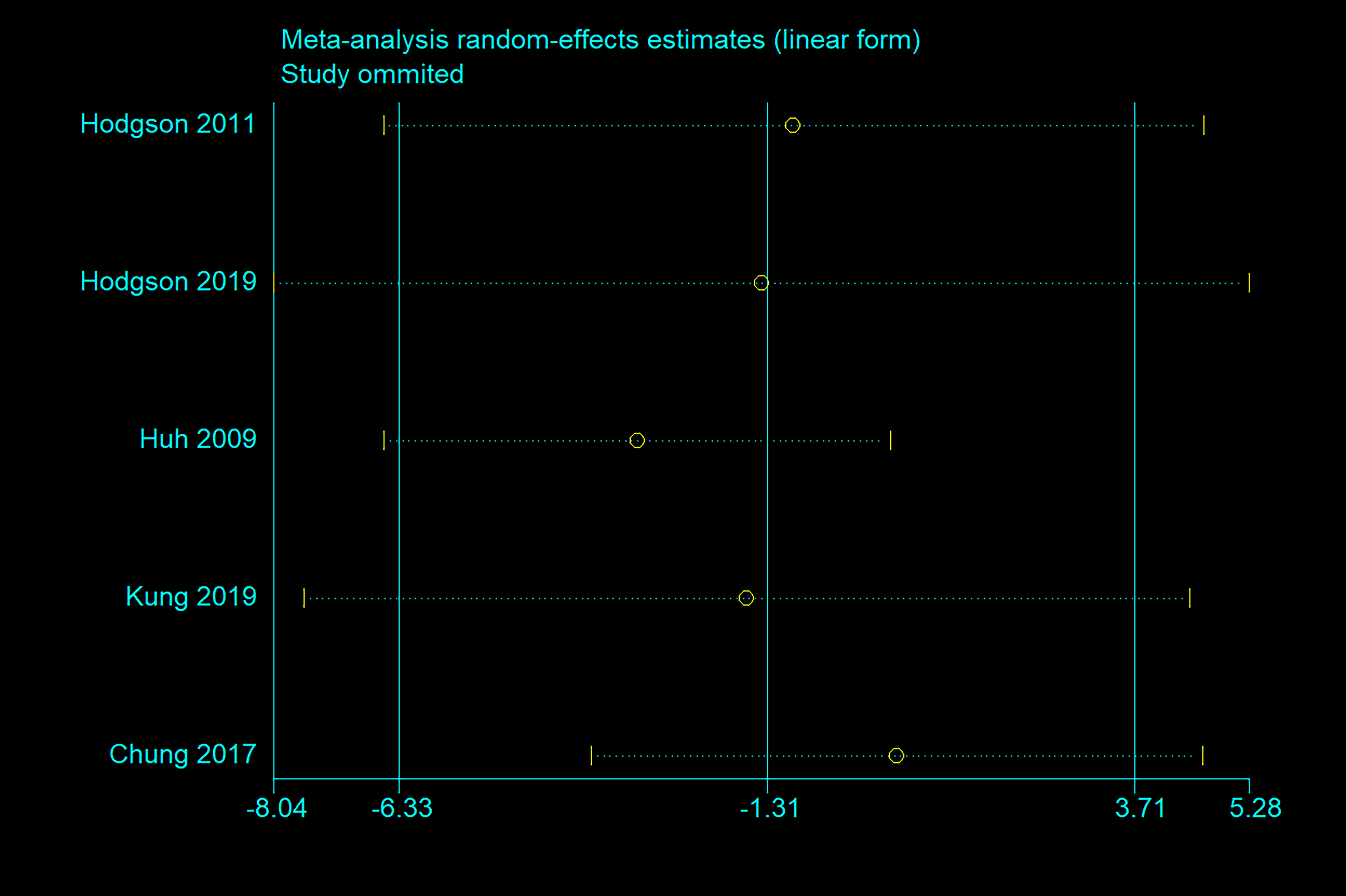

Supplement: Supplementary file 1 — Supplementary material [file mmc1.zip › Supplementary information/Supplementary information/Figure. S8b.tif]

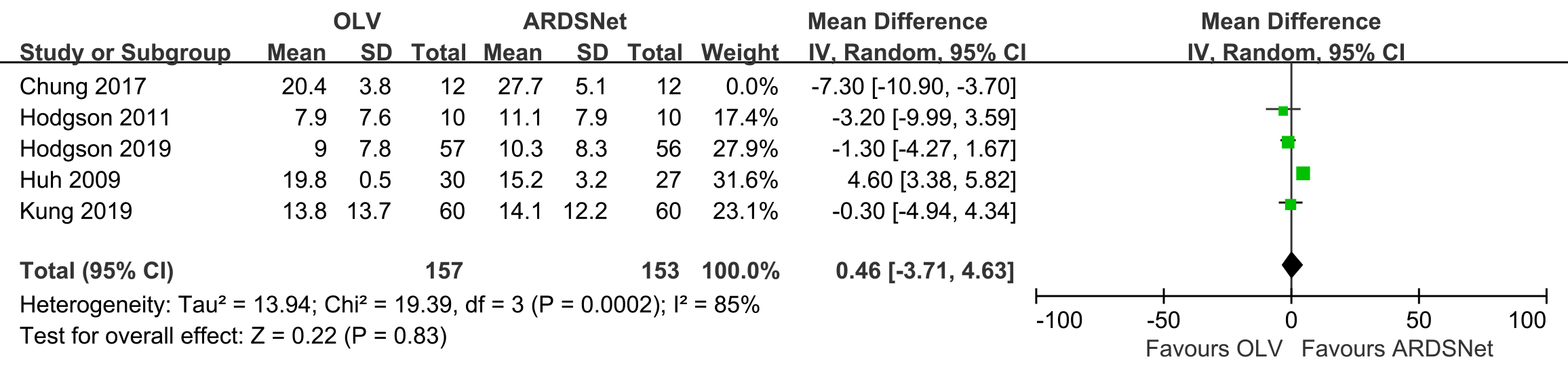

Supplement: Supplementary file 1 — Supplementary material [file mmc1.zip › Supplementary information/Supplementary information/Figure. S8c.tif]

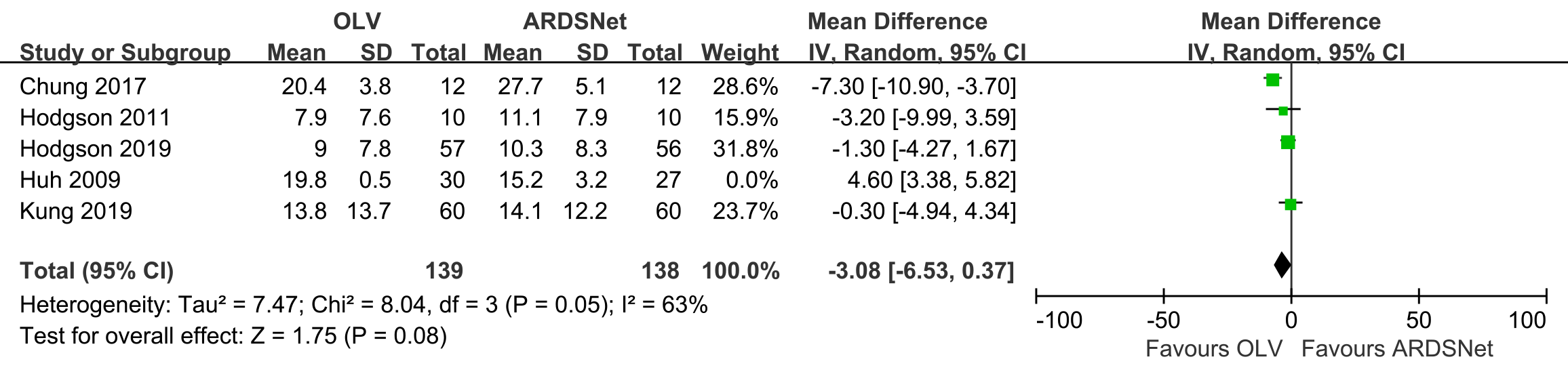

Supplement: Supplementary file 1 — Supplementary material [file mmc1.zip › Supplementary information/Supplementary information/Figure. S8d.tif]

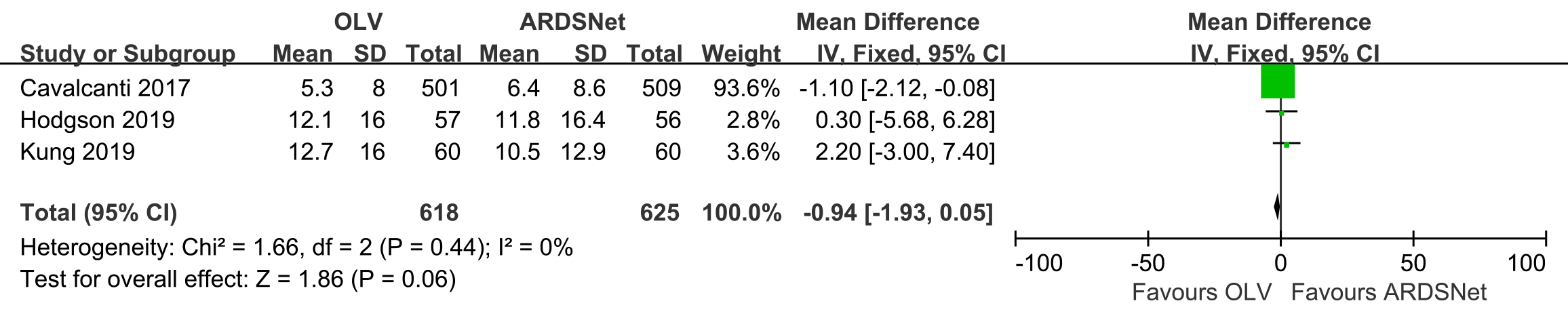

Supplement: Supplementary file 1 — Supplementary material [file mmc1.zip › Supplementary information/Supplementary information/Figure. S9a.tif]

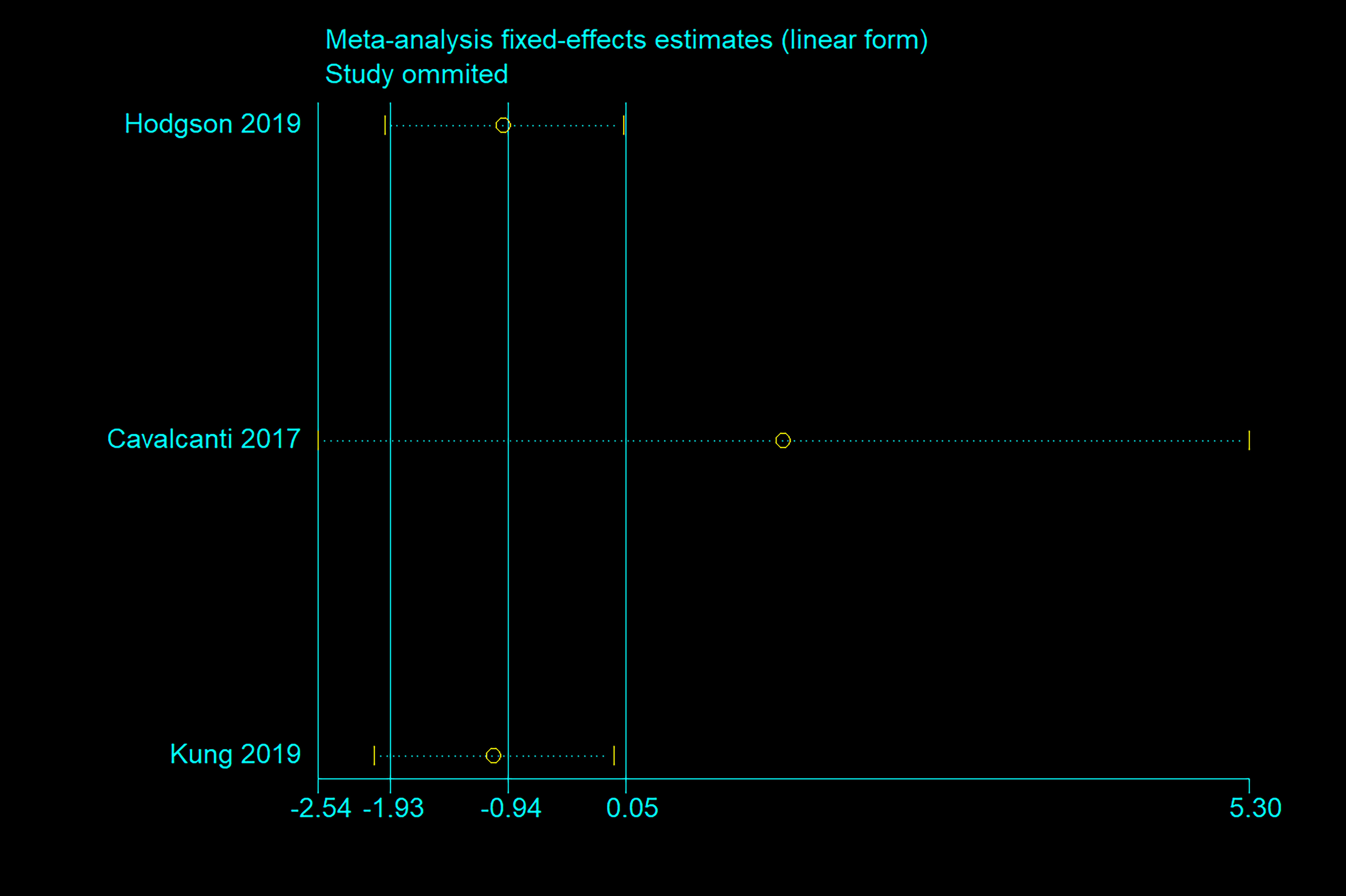

Supplement: Supplementary file 1 — Supplementary material [file mmc1.zip › Supplementary information/Supplementary information/Figure. S9b.tif]

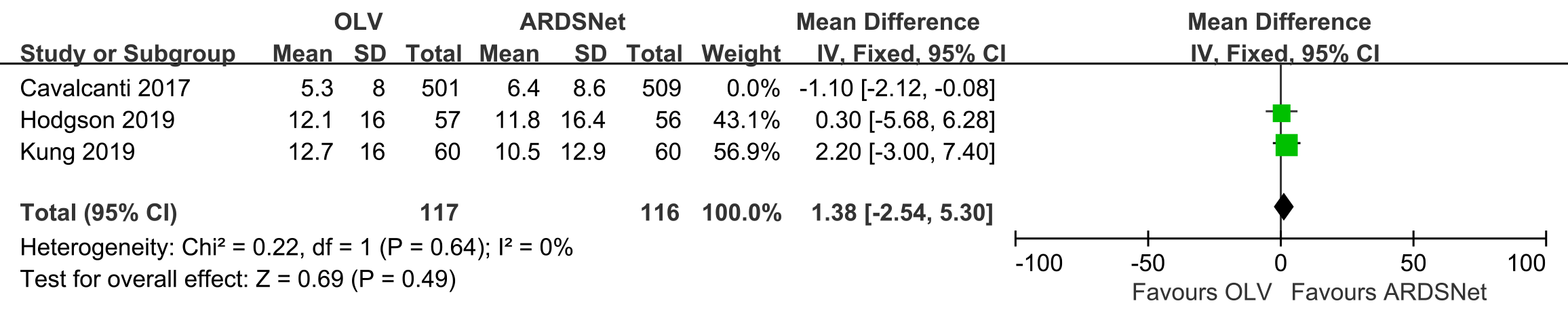

Supplement: Supplementary file 1 — Supplementary material [file mmc1.zip › Supplementary information/Supplementary information/Figure. S9c.tif]
