# Supplementary material for: Open lung ventilation with low tidal volumes, staircase recruitment maneuvers, high PEEP and decremental PEEP titration vs ARDSNet in ARDS: A systematic review and meta-analysis
Source: J Anesth Transl Med. 2025 Sep 17;4(3):148–60. doi: 10.1016/j.jatmed.2025.08.001 (PMC13001771; doi:10.1016/j.jatmed.2025.08.001)
Supplement: Supplementary file 1 — Supplementary material [file mmc1.zip › Supplementary information/Supplementary information/Supplementary Material 1. Search strategy.doc]

**Retrieval strategy**

**Disease type**

**Subject Word:**

respiratory distress syndrome

**Random word:**

respiratory distress syndromes

shock lung

acute respiratory distress syndrome

ARDS

human ARDS

adult respiratory distress syndrom

acute lung injury

ALI

**Intervention**

**Subject Word 1:**

staircase recruitment manoeuvres

**Random word 1:**

stepwise recruitment manoeuvres

maximal lung recruitment

recruitment manoeuvres

lung recruitment manoeuvres

lung recruitment

alveolar recruitment

alveolar recruitment manoeuvres

**incremental positive end expiratory pressure**

**incremental PEEP**

**Subject Word 2:**

high positive end-expiratory pressure

**Random word 2:**

high PEEP

positive end-expiratory pressure

PEEP

**Subject Word 3:**

decremental positive end-expiratory pressure titration

**Random word 3:**

positive end-expiratory pressure titration

decremental PEEP titration

decremental PEEP

PEEP titration

titrated positive end expiratory pressure

titrated PEEP

titration

titrated

**Control**

**Subject Word**

ARDSNet

**Random word**

ARDS network

acute respiratory distress syndrome network

lung protective ventilation

protective ventilation

low tidal volume

low peep

**Research method**

randomized controlled trial[Publication Type] OR randomised controlled trial[Publication Type] OR randomized[Title/Abstract] OR randomised[Title/Abstract] OR random[Title/Abstract]

1 Pubmed

****(((((((((((((respiratory distress syndrome[Title/Abstract]) OR (respiratory distress syndromes[Title/Abstract])) OR (shock lung[Title/Abstract])) OR (acute respiratory distress syndrome[Title/Abstract])) OR (ARDS[Title/Abstract])) OR (human ARDS[Title/Abstract])) OR (adult respiratory distress syndrom[Title/Abstract])) OR (acute lung injury[Title/Abstract])) OR (ALI[Title/Abstract])) AND ((((((((((staircase recruitment manoeuvres) OR (stepwise recruitment manoeuvres)) OR (maximal lung recruitment)) OR (recruitment manoeuvres)) OR (lung recruitment manoeuvres)) OR (lung recruitment)) OR (alveolar recruitment)) OR (alveolar recruitment manoeuvres)) OR (incremental positive end expiratory pressure)) OR (incremental PEEP))) AND ((((high positive end-expiratory pressure) OR (high PEEP)) OR (positive end-expiratory pressure)) OR (PEEP))) AND (((((((((decremental positive end-expiratory pressure titration) OR (positive end-expiratory pressure titration)) OR (decremental PEEP titration)) OR (decremental PEEP)) OR (PEEP titration)) OR (titrated positive end expiratory pressure)) OR (titrated PEEP)) OR (titration)) OR (titrated))) AND (((((((ARDSNet) OR (ARDS network)) OR (acute respiratory distress syndrome network)) OR (lung protective ventilation)) OR (protective ventilation)) OR (low tidal volume)) OR (low peep))) AND (randomized controlled trial[Publication Type] OR randomised controlled trial[Publication Type] OR randomized[Title/Abstract] OR randomised[Title/Abstract] OR random[Title/Abstract])****

**Outcome:**

Search number Query Sort By Filters Search Details Results Time

1 (((((((((((((respiratory distress syndrome[Title/Abstract]) OR (respiratory distress syndromes[Title/Abstract])) OR (shock lung[Title/Abstract])) OR (acute respiratory distress syndrome[Title/Abstract])) OR (ARDS[Title/Abstract])) OR (human ARDS[Title/Abstract])) OR (adult respiratory distress syndrom[Title/Abstract])) OR (acute lung injury[Title/Abstract])) OR (ALI[Title/Abstract])) AND ((((((((((staircase recruitment manoeuvres) OR (stepwise recruitment manoeuvres)) OR (maximal lung recruitment)) OR (recruitment manoeuvres)) OR (lung recruitment manoeuvres)) OR (lung recruitment)) OR (alveolar recruitment)) OR (alveolar recruitment manoeuvres)) OR (incremental positive end expiratory pressure)) OR (incremental PEEP))) AND ((((high positive end-expiratory pressure) OR (high PEEP)) OR (positive end-expiratory pressure)) OR (PEEP))) AND (((((((((decremental positive end-expiratory pressure titration) OR (positive end-expiratory pressure titration)) OR (decremental PEEP titration)) OR (decremental PEEP)) OR (PEEP titration)) OR (titrated positive end expiratory pressure)) OR (titrated PEEP)) OR (titration)) OR (titrated))) AND (((((((ARDSNet) OR (ARDS network)) OR (acute respiratory distress syndrome network)) OR (lung protective ventilation)) OR (protective ventilation)) OR (low tidal volume)) OR (low peep))) AND (randomized controlled trial[Publication Type] OR randomised controlled trial[Publication Type] OR randomized[Title/Abstract] OR randomised[Title/Abstract] OR random[Title/Abstract]) Most Recent ("respiratory distress syndrome"[Title/Abstract] OR "respiratory distress syndromes"[Title/Abstract] OR "shock lung"[Title/Abstract] OR "acute respiratory distress syndrome"[Title/Abstract] OR "ARDS"[Title/Abstract] OR "human ards"[Title/Abstract] OR "adult respiratory distress syndrom"[Title/Abstract] OR "acute lung injury"[Title/Abstract] OR "ALI"[Title/Abstract]) AND ((("staircase"[All Fields] OR "staircases"[All Fields]) AND ("recruit"[All Fields] OR "recruited"[All Fields] OR "recruiter"[All Fields] OR "recruiters"[All Fields] OR "recruiting"[All Fields] OR "recruitment"[All Fields] OR "recruitments"[All Fields] OR "recruits"[All Fields]) AND ("maneuver"[All Fields] OR "maneuvered"[All Fields] OR "maneuvering"[All Fields] OR "maneuverings"[All Fields] OR "maneuvers"[All Fields] OR "manoeuvrability"[All Fields] OR "manoeuvrable"[All Fields] OR "manoeuvre"[All Fields] OR "manoeuvred"[All Fields] OR "manoeuvres"[All Fields] OR "manoeuvring"[All Fields])) OR ("stepwise"[All Fields] AND ("recruit"[All Fields] OR "recruited"[All Fields] OR "recruiter"[All Fields] OR "recruiters"[All Fields] OR "recruiting"[All Fields] OR "recruitment"[All Fields] OR "recruitments"[All Fields] OR "recruits"[All Fields]) AND ("maneuver"[All Fields] OR "maneuvered"[All Fields] OR "maneuvering"[All Fields] OR "maneuverings"[All Fields] OR "maneuvers"[All Fields] OR "manoeuvrability"[All Fields] OR "manoeuvrable"[All Fields] OR "manoeuvre"[All Fields] OR "manoeuvred"[All Fields] OR "manoeuvres"[All Fields] OR "manoeuvring"[All Fields])) OR (("maximal"[All Fields] OR "maximally"[All Fields] OR "maximization"[All Fields] OR "maximize"[All Fields] OR "maximized"[All Fields] OR "maximizer"[All Fields] OR "maximizers"[All Fields] OR "maximizes"[All Fields] OR "maximizing"[All Fields]) AND ("lung"[MeSH Terms] OR "lung"[All Fields]) AND ("recruit"[All Fields] OR "recruited"[All Fields] OR "recruiter"[All Fields] OR "recruiters"[All Fields] OR "recruiting"[All Fields] OR "recruitment"[All Fields] OR "recruitments"[All Fields] OR "recruits"[All Fields])) OR (("recruit"[All Fields] OR "recruited"[All Fields] OR "recruiter"[All Fields] OR "recruiters"[All Fields] OR "recruiting"[All Fields] OR "recruitment"[All Fields] OR "recruitments"[All Fields] OR "recruits"[All Fields]) AND ("maneuver"[All Fields] OR "maneuvered"[All Fields] OR "maneuvering"[All Fields] OR "maneuverings"[All Fields] OR "maneuvers"[All Fields] OR "manoeuvrability"[All Fields] OR "manoeuvrable"[All Fields] OR "manoeuvre"[All Fields] OR "manoeuvred"[All Fields] OR "manoeuvres"[All Fields] OR "manoeuvring"[All Fields])) OR (("lung"[MeSH Terms] OR "lung"[All Fields]) AND ("recruit"[All Fields] OR "recruited"[All Fields] OR "recruiter"[All Fields] OR "recruiters"[All Fields] OR "recruiting"[All Fields] OR "recruitment"[All Fields] OR "recruitments"[All Fields] OR "recruits"[All Fields]) AND ("maneuver"[All Fields] OR "maneuvered"[All Fields] OR "maneuvering"[All Fields] OR "maneuverings"[All Fields] OR "maneuvers"[All Fields] OR "manoeuvrability"[All Fields] OR "manoeuvrable"[All Fields] OR "manoeuvre"[All Fields] OR "manoeuvred"[All Fields] OR "manoeuvres"[All Fields] OR "manoeuvring"[All Fields])) OR (("lung"[MeSH Terms] OR "lung"[All Fields]) AND ("recruit"[All Fields] OR "recruited"[All Fields] OR "recruiter"[All Fields] OR "recruiters"[All Fields] OR "recruiting"[All Fields] OR "recruitment"[All Fields] OR "recruitments"[All Fields] OR "recruits"[All Fields])) OR (("alveolar"[All Fields] OR "alveolarization"[All Fields] OR "alveolars"[All Fields]) AND ("recruit"[All Fields] OR "recruited"[All Fields] OR "recruiter"[All Fields] OR "recruiters"[All Fields] OR "recruiting"[All Fields] OR "recruitment"[All Fields] OR "recruitments"[All Fields] OR "recruits"[All Fields])) OR (("alveolar"[All Fields] OR "alveolarization"[All Fields] OR "alveolars"[All Fields]) AND ("recruit"[All Fields] OR "recruited"[All Fields] OR "recruiter"[All Fields] OR "recruiters"[All Fields] OR "recruiting"[All Fields] OR "recruitment"[All Fields] OR "recruitments"[All Fields] OR "recruits"[All Fields]) AND ("maneuver"[All Fields] OR "maneuvered"[All Fields] OR "maneuvering"[All Fields] OR "maneuverings"[All Fields] OR "maneuvers"[All Fields] OR "manoeuvrability"[All Fields] OR "manoeuvrable"[All Fields] OR "manoeuvre"[All Fields] OR "manoeuvred"[All Fields] OR "manoeuvres"[All Fields] OR "manoeuvring"[All Fields])) OR (("incremental"[All Fields] OR "incrementally"[All Fields]) AND ("positive pressure respiration"[MeSH Terms] OR ("positive pressure"[All Fields] AND "respiration"[All Fields]) OR "positive pressure respiration"[All Fields] OR ("positive"[All Fields] AND "end"[All Fields] AND "expiratory"[All Fields] AND "pressure"[All Fields]) OR "positive end expiratory pressure"[All Fields])) OR (("incremental"[All Fields] OR "incrementally"[All Fields]) AND ("positive pressure respiration"[MeSH Terms] OR ("positive pressure"[All Fields] AND "respiration"[All Fields]) OR "positive pressure respiration"[All Fields] OR "peep"[All Fields]))) AND (("high"[All Fields] AND ("positive pressure respiration"[MeSH Terms] OR ("positive pressure"[All Fields] AND "respiration"[All Fields]) OR "positive pressure respiration"[All Fields] OR ("positive"[All Fields] AND "end"[All Fields] AND "expiratory"[All Fields] AND "pressure"[All Fields]) OR "positive end expiratory pressure"[All Fields])) OR ("high"[All Fields] AND ("positive pressure respiration"[MeSH Terms] OR ("positive pressure"[All Fields] AND "respiration"[All Fields]) OR "positive pressure respiration"[All Fields] OR "peep"[All Fields])) OR ("positive pressure respiration"[MeSH Terms] OR ("positive pressure"[All Fields] AND "respiration"[All Fields]) OR "positive pressure respiration"[All Fields] OR ("positive"[All Fields] AND "end"[All Fields] AND "expiratory"[All Fields] AND "pressure"[All Fields]) OR "positive end expiratory pressure"[All Fields]) OR ("positive pressure respiration"[MeSH Terms] OR ("positive pressure"[All Fields] AND "respiration"[All Fields]) OR "positive pressure respiration"[All Fields] OR "peep"[All Fields])) AND ((("decremental"[All Fields] OR "decrementally"[All Fields]) AND ("positive pressure respiration"[MeSH Terms] OR ("positive pressure"[All Fields] AND "respiration"[All Fields]) OR "positive pressure respiration"[All Fields] OR ("positive"[All Fields] AND "end"[All Fields] AND "expiratory"[All Fields] AND "pressure"[All Fields]) OR "positive end expiratory pressure"[All Fields]) AND ("titrate"[All Fields] OR "titrated"[All Fields] OR "titrates"[All Fields] OR "titrating"[All Fields] OR "titrator"[All Fields] OR "titrators"[All Fields] OR "titrimetry"[MeSH Terms] OR "titrimetry"[All Fields] OR "titration"[All Fields] OR "titrations"[All Fields])) OR (("positive pressure respiration"[MeSH Terms] OR ("positive pressure"[All Fields] AND "respiration"[All Fields]) OR "positive pressure respiration"[All Fields] OR ("positive"[All Fields] AND "end"[All Fields] AND "expiratory"[All Fields] AND "pressure"[All Fields]) OR "positive end expiratory pressure"[All Fields]) AND ("titrate"[All Fields] OR "titrated"[All Fields] OR "titrates"[All Fields] OR "titrating"[All Fields] OR "titrator"[All Fields] OR "titrators"[All Fields] OR "titrimetry"[MeSH Terms] OR "titrimetry"[All Fields] OR "titration"[All Fields] OR "titrations"[All Fields])) OR (("decremental"[All Fields] OR "decrementally"[All Fields]) AND ("positive pressure respiration"[MeSH Terms] OR ("positive pressure"[All Fields] AND "respiration"[All Fields]) OR "positive pressure respiration"[All Fields] OR "peep"[All Fields]) AND ("titrate"[All Fields] OR "titrated"[All Fields] OR "titrates"[All Fields] OR "titrating"[All Fields] OR "titrator"[All Fields] OR "titrators"[All Fields] OR "titrimetry"[MeSH Terms] OR "titrimetry"[All Fields] OR "titration"[All Fields] OR "titrations"[All Fields])) OR (("decremental"[All Fields] OR "decrementally"[All Fields]) AND ("positive pressure respiration"[MeSH Terms] OR ("positive pressure"[All Fields] AND "respiration"[All Fields]) OR "positive pressure respiration"[All Fields] OR "peep"[All Fields])) OR (("positive pressure respiration"[MeSH Terms] OR ("positive pressure"[All Fields] AND "respiration"[All Fields]) OR "positive pressure respiration"[All Fields] OR "peep"[All Fields]) AND ("titrate"[All Fields] OR "titrated"[All Fields] OR "titrates"[All Fields] OR "titrating"[All Fields] OR "titrator"[All Fields] OR "titrators"[All Fields] OR "titrimetry"[MeSH Terms] OR "titrimetry"[All Fields] OR "titration"[All Fields] OR "titrations"[All Fields])) OR (("titrate"[All Fields] OR "titrated"[All Fields] OR "titrates"[All Fields] OR "titrating"[All Fields] OR "titrator"[All Fields] OR "titrators"[All Fields] OR "titrimetry"[MeSH Terms] OR "titrimetry"[All Fields] OR "titration"[All Fields] OR "titrations"[All Fields]) AND ("positive pressure respiration"[MeSH Terms] OR ("positive pressure"[All Fields] AND "respiration"[All Fields]) OR "positive pressure respiration"[All Fields] OR ("positive"[All Fields] AND "end"[All Fields] AND "expiratory"[All Fields] AND "pressure"[All Fields]) OR "positive end expiratory pressure"[All Fields])) OR (("titrate"[All Fields] OR "titrated"[All Fields] OR "titrates"[All Fields] OR "titrating"[All Fields] OR "titrator"[All Fields] OR "titrators"[All Fields] OR "titrimetry"[MeSH Terms] OR "titrimetry"[All Fields] OR "titration"[All Fields] OR "titrations"[All Fields]) AND ("positive pressure respiration"[MeSH Terms] OR ("positive pressure"[All Fields] AND "respiration"[All Fields]) OR "positive pressure respiration"[All Fields] OR "peep"[All Fields])) OR ("titrate"[All Fields] OR "titrated"[All Fields] OR "titrates"[All Fields] OR "titrating"[All Fields] OR "titrator"[All Fields] OR "titrators"[All Fields] OR "titrimetry"[MeSH Terms] OR "titrimetry"[All Fields] OR "titration"[All Fields] OR "titrations"[All Fields]) OR ("titrate"[All Fields] OR "titrated"[All Fields] OR "titrates"[All Fields] OR "titrating"[All Fields] OR "titrator"[All Fields] OR "titrators"[All Fields] OR "titrimetry"[MeSH Terms] OR "titrimetry"[All Fields] OR "titration"[All Fields] OR "titrations"[All Fields])) AND ("ARDSNet"[All Fields] OR (("respiratory distress syndrome"[MeSH Terms] OR ("respiratory"[All Fields] AND "distress"[All Fields] AND "syndrome"[All Fields]) OR "respiratory distress syndrome"[All Fields] OR "ARDS"[All Fields]) AND ("network"[All Fields] OR "network s"[All Fields] OR "networked"[All Fields] OR "networker"[All Fields] OR "networkers"[All Fields] OR "networking"[All Fields] OR "networks"[All Fields])) OR (("respiratory distress syndrome"[MeSH Terms] OR ("respiratory"[All Fields] AND "distress"[All Fields] AND "syndrome"[All Fields]) OR "respiratory distress syndrome"[All Fields] OR ("acute"[All Fields] AND "respiratory"[All Fields] AND "distress"[All Fields] AND "syndrome"[All Fields]) OR "acute respiratory distress syndrome"[All Fields]) AND ("network"[All Fields] OR "network s"[All Fields] OR "networked"[All Fields] OR "networker"[All Fields] OR "networkers"[All Fields] OR "networking"[All Fields] OR "networks"[All Fields])) OR (("lung"[MeSH Terms] OR "lung"[All Fields]) AND ("protect"[All Fields] OR "protected"[All Fields] OR "protecting"[All Fields] OR "protection"[All Fields] OR "protections"[All Fields] OR "protective agents"[Pharmacological Action] OR "protective agents"[MeSH Terms] OR ("protective"[All Fields] AND "agents"[All Fields]) OR "protective agents"[All Fields] OR "protectant"[All Fields] OR "protectants"[All Fields] OR "protective"[All Fields] OR "protectively"[All Fields] OR "protectiveness"[All Fields] OR "protectives"[All Fields] OR "protects"[All Fields]) AND ("ventilated"[All Fields] OR "ventilates"[All Fields] OR "ventilating"[All Fields] OR "ventilation"[MeSH Terms] OR "ventilation"[All Fields] OR "ventilate"[All Fields] OR "ventilations"[All Fields] OR "ventilation s"[All Fields] OR "ventilator s"[All Fields] OR "ventilators, mechanical"[MeSH Terms] OR ("ventilators"[All Fields] AND "mechanical"[All Fields]) OR "mechanical ventilators"[All Fields] OR "ventilator"[All Fields] OR "ventilators"[All Fields] OR "ventillation"[All Fields])) OR (("protect"[All Fields] OR "protected"[All Fields] OR "protecting"[All Fields] OR "protection"[All Fields] OR "protections"[All Fields] OR "protective agents"[Pharmacological Action] OR "protective agents"[MeSH Terms] OR ("protective"[All Fields] AND "agents"[All Fields]) OR "protective agents"[All Fields] OR "protectant"[All Fields] OR "protectants"[All Fields] OR "protective"[All Fields] OR "protectively"[All Fields] OR "protectiveness"[All Fields] OR "protectives"[All Fields] OR "protects"[All Fields]) AND ("ventilated"[All Fields] OR "ventilates"[All Fields] OR "ventilating"[All Fields] OR "ventilation"[MeSH Terms] OR "ventilation"[All Fields] OR "ventilate"[All Fields] OR "ventilations"[All Fields] OR "ventilation s"[All Fields] OR "ventilator s"[All Fields] OR "ventilators, mechanical"[MeSH Terms] OR ("ventilators"[All Fields] AND "mechanical"[All Fields]) OR "mechanical ventilators"[All Fields] OR "ventilator"[All Fields] OR "ventilators"[All Fields] OR "ventillation"[All Fields])) OR ("low"[All Fields] AND ("tidal volume"[MeSH Terms] OR ("tidal"[All Fields] AND "volume"[All Fields]) OR "tidal volume"[All Fields])) OR ("low"[All Fields] AND ("positive pressure respiration"[MeSH Terms] OR ("positive pressure"[All Fields] AND "respiration"[All Fields]) OR "positive pressure respiration"[All Fields] OR "peep"[All Fields]))) AND ("randomized controlled trial"[Publication Type] OR "randomized"[Title/Abstract] OR "randomised"[Title/Abstract] OR "random"[Title/Abstract]) 33 23:44:27

2 Embase

'Respiratory Distress Syndrome':ab,ti OR 'respiratory distress syndromes':ab,ti OR 'shock lung':ab,ti OR 'acute respiratory distress syndrome':ab,ti OR 'ARDS':ab,ti OR 'human ARDS':ab,ti OR 'adult respiratory distress syndrom':ab,ti OR 'acute lung injury':ab,ti OR 'ALI':ab,ti

'staircase recruitment manoeuvres' OR 'stepwise recruitment manoeuvres' OR 'maximal lung recruitment' OR 'recruitment manoeuvres' OR 'lung recruitment manoeuvres' OR 'lung recruitment' OR 'alveolar recruitment' OR 'alveolar recruitment manoeuvres' OR 'incremental positive end expiratory pressure' OR 'incremental PEEP'

'high positive end-expiratory pressure' OR 'high PEEP' OR 'positive end-expiratory pressure' OR 'PEEP'

'decremental positive end-expiratory pressure titration' OR 'positive end-expiratory pressure titration' OR 'decremental PEEP titration' OR 'decremental PEEP' OR 'PEEP titration' OR 'titrated positive end expiratory pressure' OR 'titrated PEEP' OR 'titration' OR 'titrated'

'ARDSNet' OR 'ARDS network' OR 'acute respiratory distress syndrome network' OR 'lung protective ventilation' OR 'protective ventilation' OR 'low tidal volume' OR 'low PEEP'

'randomized controlled trial':ab,ti OR 'randomised controlled trial':ab,ti OR 'randomized':ab,ti OR 'randomised':ab,ti OR 'random':ab,ti

**Outcome:**

Embase

Session Results

.......................................................

No. Query Results	Results Date

#7. #1 AND #2 AND #3 AND #4 AND #5 AND #6	24 3 Jan 2025

#6. 'randomized controlled trial':ab,ti OR	1,677,628 3 Jan 2025

'randomised controlled trial':ab,ti OR

'randomized':ab,ti OR 'randomised':ab,ti OR

'random':ab,ti

#5. 'ardsnet' OR 'ards network' OR 'acute respiratory	6,242 3 Jan 2025

distress syndrome network' OR 'lung protective

ventilation' OR 'protective ventilation' OR 'low

tidal volume' OR 'low peep'

#4. 'decremental positive end-expiratory pressure	129,487 3 Jan 2025

titration' OR 'positive end-expiratory pressure

titration' OR 'decremental peep titration' OR

'decremental peep' OR 'peep titration' OR

'titrated positive end expiratory pressure' OR

'titrated peep' OR 'titration' OR 'titrated'

#3. 'high positive end-expiratory pressure' OR 'high	68,619 3 Jan 2025

peep' OR 'positive end-expiratory pressure' OR

'peep'

#2. 'staircase recruitment manoeuvres' OR 'stepwise	2,684 3 Jan 2025

recruitment manoeuvres' OR 'maximal lung

recruitment' OR 'recruitment manoeuvres' OR 'lung

recruitment manoeuvres' OR 'lung recruitment' OR

'alveolar recruitment' OR 'alveolar recruitment

manoeuvres' OR 'incremental positive end

expiratory pressure' OR 'incremental peep'

#1. 'respiratory distress syndrome':ab,ti OR	93,499 3 Jan 2025

'respiratory distress syndromes':ab,ti OR 'shock

lung':ab,ti OR 'acute respiratory distress

syndrome':ab,ti OR 'ards':ab,ti OR 'human

ards':ab,ti OR 'adult respiratory distress

syndrom':ab,ti OR 'acute lung injury':ab,ti OR

'ali':ab,ti

.......................................................

3 Cochrane

(respiratory distress syndrome):ti,ab,kw or (respiratory distress syndromes):ti,ab,kw or (shock lung):ti,ab,kw or (acute respiratory distress syndrome):ti,ab,kw or (ARDS):ti,ab,kw or (human ARDS):ti,ab,kw or (adult respiratory distress syndrom):ti,ab,kw or (acute lung injury):ti,ab,kw or (ALI):ti,ab,kw

(staircase recruitment manoeuvres) or (stepwise recruitment manoeuvres) or (maximal lung recruitment) or (recruitment manoeuvres) or (lung recruitment manoeuvres) or (lung recruitment) or (alveolar recruitment) or (alveolar recruitment manoeuvres ) or (incremental positive end expiratory pressure) or (incremental PEEP)

(high positive end-expiratory pressure) or (high PEEP) or (positive end-expiratory pressure) or (PEEP)

(decremental positive end-expiratory pressure titration) or (positive end-expiratory pressure titration) or (decremental PEEP titration) or (decremental PEEP) or (PEEP titration) or (titrated positive end expiratory pressure) or (titrated PEEP) or (titration) or (titrated)

(ARDSNet) or (ARDS network) or (acute respiratory distress syndrome network) or (lung protective ventilation) or (protective ventilation) or (low tidal volume) or (low peep)

(randomized controlled trial):ab or (randomised controlled trial):ab or (randomized):ab or (randomised):ab or (random):ab

**Outcome:**

Search Name:

Date Run:	03/01/2025 13:04:32

Comment:

ID	Search	Hits

#1	(respiratory distress syndrome):ti,ab,kw or (respiratory distress syndromes):ti,ab,kw or (shock lung):ti,ab,kw or (acute respiratory distress syndrome):ti,ab,kw or (ARDS):ti,ab,kw or (human ARDS):ti,ab,kw or (adult respiratory distress syndrom):ti,ab,kw or (acute lung injury):ti,ab,kw or (ALI):ti,ab,kw	10862

#2	(staircase recruitment manoeuvres) or (stepwise recruitment manoeuvres) or (maximal lung recruitment) or (recruitment manoeuvres) or (lung recruitment manoeuvres) or (lung recruitment) or (alveolar recruitment) or (alveolar recruitment manoeuvres ) or (incremental positive end expiratory pressure) or (incremental PEEP)	4333

#3	(high positive end-expiratory pressure) or (high PEEP) or (positive end-expiratory pressure) or (PEEP)	6183

#4	(decremental positive end-expiratory pressure titration) or (positive end-expiratory pressure titration) or (decremental PEEP titration) or (decremental PEEP) or (PEEP titration) or (titrated positive end expiratory pressure) or (titrated PEEP) or (titration) or (titrated)	19742

#5	(ARDSNet) or (ARDS network) or (acute respiratory distress syndrome network) or (lung protective ventilation) or (protective ventilation) or (low tidal volume) or (low peep)	3567

#6	(randomized controlled trial):ab or (randomised controlled trial):ab or (randomized):ab or (randomised):ab or (random):ab	938242

#7	#1 and #2 and #3 and #4 and #5 and #6	37

4 Web of Sciense

respiratory distress syndrome or respiratory distress syndromes or shock lung or acute respiratory distress syndrome or ARDS or human ARDS or adult respiratory distress syndrom or acute lung injury or ALI

staircase recruitment manoeuvres or stepwise recruitment manoeuvres or maximal lung recruitment or recruitment manoeuvres or lung recruitment manoeuvres or lung recruitment or alveolar recruitment or alveolar recruitment manoeuvres or incremental positive end expiratory pressure or incremental PEEP

high positive end-expiratory pressure or high PEEP or positive end-expiratory pressure or PEEP

decremental positive end-expiratory pressure titration or positive end-expiratory pressure titration or decremental PEEP titration or decremental PEEP or PEEP titration or titrated positive end expiratory pressure or titrated PEEP or titration or titrated

ARDSNet or ARDS network or acute respiratory distress syndrome network or lung protective ventilation or protective ventilation or low tidal volume or low PEEP

randomized controlled trial or randomised controlled trial or randomized or randomised or random

**Outcome:**

\# Web of Science Search Strategy (v0.1)

\# Database: All Databases

\# Entitlements:

- WOS: 1982 to 2024

- CSCD: 1989 to 2024

- DIIDW: 1966 to 2024

- GRANTS: 1953 to 2024

- INSPEC: 1969 to 2024

- KJD: 1980 to 2024

- MEDLINE: 1950 to 2024

- PPRN: 1991 to 2024

- PQDT: 1637 to 2024

- SCIELO: 2002 to 2024

\# Searches:

1: respiratory distress syndrome or respiratory distress syndromes or shock lung or acute respiratory distress syndrome or ARDS or human ARDS or adult respiratory distress syndrom or acute lung injury or ALI (Topic) AND staircase recruitment manoeuvres or stepwise recruitment manoeuvres or maximal lung recruitment or recruitment manoeuvres or lung recruitment manoeuvres or lung recruitment or alveolar recruitment or alveolar recruitment manoeuvres or incremental positive end expiratory pressure or incremental PEEP (Topic) AND high positive end-expiratory pressure or high PEEP or positive end-expiratory pressure or PEEP (Topic) AND decremental positive end-expiratory pressure titration or positive end-expiratory pressure titration or decremental PEEP titration or decremental PEEP or PEEP titration or titrated positive end expiratory pressure or titrated PEEP or titration or titrated (Topic) AND ARDSNet or ARDS network or acute respiratory distress syndrome network or lung protective ventilation or protective ventilation or low tidal volume or low PEEP (Topic) AND randomized controlled trial or randomised controlled trial or randomized or randomised or random (Topic) and Preprint Citation Index (Exclude – Database)	Date Run: Fri Jan 03 2025 12:54:10 GMT+0800 (GMT+08:00)	Results: 77

5 Scopus

"respiratory distress syndrome" OR "respiratory distress syndromes" OR "shock lung" OR "acute respiratory distress syndrome" OR "ARDS" OR "human ARDS" OR "adult respiratory distress syndrom" OR "acute lung injury" OR "ALI"

"staircase recruitment manoeuvres" OR "stepwise recruitment manoeuvres" OR "maximal lung recruitment" OR "recruitment manoeuvres" OR "lung recruitment manoeuvres" OR "lung recruitment" OR "alveolar recruitment" OR "alveolar recruitment manoeuvres " OR "incremental positive end expiratory pressure" OR "incremental PEEP"

"high positive end-expiratory pressure" OR "high PEEP" OR "positive end-expiratory pressure" OR "PEEP"

"decremental positive end-expiratory pressure titration" OR "positive end-expiratory pressure titration" OR "decremental PEEP titration" OR "decremental PEEP" OR "PEEP titration" OR "titrated positive end expiratory pressure" OR "titrated PEEP" OR "titration" OR "titrated"

"ARDSNet" OR "ARDS network" OR "acute respiratory distress syndrome network" OR "lung protective ventilation" OR "protective ventilation" OR "low tidal volume" OR "low PEEP"

"randomized controlled trial" OR "randomised controlled trial" OR "randomized" OR "randomised" OR "random"

**Outcome:**

( TITLE-ABS-KEY ( "respiratory distress syndrome" OR "respiratory distress syndromes" OR "shock lung" OR "acute respiratory distress syndrome" OR "ARDS" OR "human ARDS" OR "adult respiratory distress syndrom" OR "acute lung injury" OR "ALI" ) AND TITLE-ABS-KEY ( "open lung strategy" OR "staircase recruitment manoeuvres" OR "high positive end-expiratory pressure" OR "decremental positive end-expiratory pressure" OR "positive end-expiratory pressure titration" OR "permissive hypercapnia" OR "open lung approach" OR "open lung ventilation" OR "open lung" OR "opening the lung" OR "OLS" OR "maximal lung recruitment" OR "recruitment manoeuvres" OR "lung recruitment manoeuvres" OR "lung recruitment" OR "alveolar recruitment" OR "alveolar recruitment manoeuvres " OR "SRM" OR "ARM" OR "RM" OR "high PEEP" OR "decremental PEEP" OR "decremental PEEP titration" OR "PEEP titration" OR "titrated positive end expiratory pressure" OR "titrated PEEP" ) AND TITLE-ABS-KEY ( "lung protective ventilation" OR "ARDSnet" OR "conventional levels of positive end-expiratory pressure" OR "low tidal volume" OR "protective ventilation" OR "ARDS net" OR "ARDS network" OR "Acute Respiratory Distress Syndrome network" OR "conventional levels of PEEP" ) AND ABS ( "randomized controlled trial" OR "randomised controlled trial" OR "randomized" OR "randomised" OR "random" ) )
